# MicroRNA-Based Risk Score for Predicting Tumor Progression Following Radioactive Iodine Ablation in Well-Differentiated Thyroid Cancer Patients: A Propensity-Score Matched Analysis

**DOI:** 10.3390/cancers13184649

**Published:** 2021-09-16

**Authors:** Eman A. Toraih, Manal S. Fawzy, Mohammad H. Hussein, Mohamad M. El-Labban, Emmanuelle M. L. Ruiz, Abdallah A. Attia, Shams Halat, Krzysztof Moroz, Youssef Errami, Mourad Zerfaoui, Emad Kandil

**Affiliations:** 1 Department of Surgery, Tulane University School of Medicine, New Orleans, LA 70112, USA; mhussein1@tulane.edu (M.H.H.); remmanuelle@tulane.edu (E.M.L.R.); aattia@tulane.edu (A.A.A.); yerrami@tulane.edu (Y.E.); mzerfaoui@tulane.edu (M.Z.); ekandil@tulane.edu (E.K.); 2 Genetics Unit, Department of Histology and Cell Biology, Suez Canal University, Ismailia 41522, Egypt; 3 Department of Medical Biochemistry and Molecular Biology, Faculty of Medicine, Suez Canal University, Ismailia 41522, Egypt; manal2_khashana@ymail.com; 4 Department of Biochemistry, Faculty of Medicine, Northern Border University, Arar 1321, Saudi Arabia; 5 Department of Pathology, Faculty of Medicine, Port Said University, Port Said 42526, Egypt; dr.labban@gmail.com; 6 Department of Pathology & Laboratory Medicine, Tulane University School of Medicine, New Orleans, LA 70112, USA; shalat@tulane.edu (S.H.); kmoroz@tulane.edu (K.M.)

**Keywords:** thyroid cancer, RAI, risk score, microRNAs, miR-221, miR-222, miR-204, nomogram, progress, survival

## Abstract

**Simple Summary:**

The three-tiered American Thyroid Association (ATA) risk stratification helps clinicians tailor decisions regarding follow-up modalities and the need for postoperative radioactive iodine (RAI) ablation and radiotherapy. However, a significant number of well-differentiated thyroid cancers (DTC) progress after treatment. Current follow-up modalities have also been proposed to detect disease relapse and recurrence but have failed to be sufficiently sensitive or specific to detect, monitor, or determine progression. Therefore, we assessed the predictive accuracy of the microRNA-based risk score in DTC with and without postoperative RAI. We confirm the prognostic role of triad biomarkers (miR-2f04, miR-221, and miR-222) with higher sensitivity and specificity for predicting disease progression than the ATA risk score. Compared to indolent tumors, a higher risk score was found in progressive samples and was associated with shorter survival. Consequently, our prognostic microRNA signature and nomogram provide a clinically practical and reliable ancillary measure to determine the prognosis of DTC patients.

**Abstract:**

To identify molecular markers that can accurately predict aggressive tumor behavior at the time of surgery, a propensity-matching score analysis of archived specimens yielded two similar datasets of DTC patients (with and without RAI). Bioinformatically selected microRNAs were quantified by qRT-PCR. The risk score was generated using Cox regression and assessed using ROC, C-statistic, and Brier-score. A predictive Bayesian nomogram was established. External validation was performed, and causal network analysis was generated. Within the eight-year follow-up period, progression was reported in 51.5% of cases; of these, 48.6% had the T1a/b stage. Analysis showed upregulation of miR-221-3p and miR-222-3p and downregulation of miR-204-5p in 68 paired cancer tissues (*p* < 0.001). These three miRNAs were not differentially expressed in RAI and non-RAI groups. The ATA risk score showed poor discriminative ability (AUC = 0.518, *p* = 0.80). In contrast, the microRNA-based risk score showed high accuracy in predicting tumor progression in the whole cohorts (median = 1.87 vs. 0.39, AUC = 0.944) and RAI group (2.23 vs. 0.37, AUC = 0.979) at the cutoff >0.86 (92.6% accuracy, 88.6% sensitivity, 97% specificity) in the whole cohorts (C-statistics = 0.943/Brier = 0.083) and RAI subgroup (C-statistic = 0.978/Brier = 0.049). The high-score group had a three-fold increased progression risk (hazard ratio = 2.71, 95%CI = 1.86–3.96, *p* < 0.001) and shorter survival times (17.3 vs. 70.79 months, *p* < 0.001). Our prognostic microRNA signature and nomogram showed excellent predictive accuracy for progression-free survival in DTC.

## 1. Introduction

The incidence of thyroid cancer (TC) in the United States (US) was estimated to be over 52,890 cases (12,720 men and 40,170 women) in 2020 and is projected to be the fourth most common cancer by 2030 [1]. The annual incidence has increased by 211% over the last two decades, mostly due to increased detection of papillary thyroid microcarcinoma (PTMC) [2,3,4]. Papillary thyroid cancer (PTC) and follicular thyroid cancer (FTC) account for most thyroid cancers [5]. These well-differentiated thyroid cancers (DTC) are generally treated with surgical resections followed by adjuvant radioactive iodine (RAI) therapy to ablate the remnant or residual thyroid tissue [6,7]. Following surgery, patients suffer from a significant risk of complications, need for hormone replacement therapy, and lengthy postoperative surveillance with unnecessarily higher health care costs and diminished quality of life.

Based on the revised 2015 American Thyroid Association (ATA) guidelines, DTC patients were categorized into low, intermediate, and high-risk groups according to the estimated risk of recurrence and cancer relapse [8]. This three-tiered risk stratification system helps to tailor decisions regarding the need for postoperative thyrotropin suppression, radioactive iodine ablation, or radiotherapy, as well as the frequency and modality of follow-up studies required [9]. Despite the good prognosis of DTC, up to 30% of patients experience recurrences in the thyroid bed or neck lymph nodes after initial treatment [10]. The modalities currently used for TC assessment, including ultrasound and fine-needle aspiration biopsy, offer only a snapshot of the disease in a single point of time and do not describe tumor behavior over time. The sonographic appearance and cytopathology findings may sometimes be non-informative and inconclusive [11]. Several clinical parameters have also been proposed but have failed to be sufficiently sensitive or specific to detect, monitor, or determine progression. Serum thyroglobulin (sTg) has been reported as a predictor for treatment efficacy during ablative radioiodine treatment [12]; however, some patients still exhibit elevated sTg levels even after receiving adjuvant RAI therapy [13,14]. As reported by Yim et al. [15], 11% of PTC patients who underwent bilateral thyroidectomy followed by RAI remnant ablation developed recurrence, with only 36.1% of these showing high sTg levels.

Similarly, in a study by Hirsch et al., 47% had a persistent disease despite re-treatment with RAI [16]. To date, there are no molecular markers that can predict tumor recurrence or persistence. Hence, there is a critical need to discover new biomarkers to biologically define which cancers have an aggressive form and optimize the selection criteria of management plans. In the absence of such a prognostic panel, prediction for progression will continue to be a challenging practice that will impede well-being and make patients and clinicians reluctant to choose active surveillance instead of surgical intervention. 

As central regulators of gene expression, microRNAs (miRNAs) are attracting increasing attention because of their association with tumor development and progression. MiRNAs are short non-coding RNAs of around 18–23 nucleotides that regulate virtually all biological functions via post-transcriptional gene silencing [17]. MiRNAs can act as either oncogenes or tumor suppressor genes in thyroid cancer [18]. Altered expression levels of miRNAs influence apoptosis, migration and proliferation, angiogenesis, and local immune response. Distinct miRNA expression profiles are also associated with well-defined clinicopathological features of thyroid cancer [19] and prognosis/disease progression [20], as depicted in in vitro and in vivo studies. 

There is increasing interest in the association of miRNA expression with chemo- and radiosensitivity for predicting or modulating resistance [21]. For example, upregulation of the miRNA-221/-222 and miRNA-17-92 cluster significantly increases the radioresistance of cancer cells through the downregulation of phosphatase and tension homolog (PTEN) and pAkt activity [22,23], while miR-145 treatment effectively increases the sensitivity of cells to radiation [24]. Furthermore, emerging evidence also demonstrates miRNAs as promising therapeutic targets in thyroid cancer. Upregulation of the miR-17-92 cluster could provide a promising therapeutic modality to counteract ATC progression [25,26]. Similarly, miR-204-5p upregulation plays a protective role by inhibiting PTC cell proliferation through regulating IGFBP5 expression [27,28]. In contrast, inhibiting oncomiRs inducing metastasis such as miR-146a and miR-146b in PTC cells through targeting IRAK1 or Wnt/β-catenin pathways might provide ancillary therapeutic strategies in conjunction with the current status quo regimens [29,30,31,32]. Therefore, reversing the altered miRNA signature may pave the road toward a cure. 

No specific miRNA panel has overcome the hurdle of predicting recurrence and response to therapy, especially in the absence of lymph node metastasis and extrathyroidal extension. Therefore, we aimed to identify and validate a microRNome signature to predict recurrence at the time of surgery in well-differentiated thyroid cancer patients following radioactive iodine ablation. Analysis of TC datasets in The Cancer Genome Atlas (TCGA) database revealed several deregulated miRNAs in patients who developed recurrent/persistent disease postoperatively. Of these, miR-221, miR-222, and miR-204 consistently predicted recurrence before and after radioactive remnant ablation treatment. We validated our findings in genome-wide miRNA expression profiling studies and patient samples. Our results demonstrate the putative role of the triad biomarker as an effective prognostic signature that accurately predicts recurrence following RAI treatment in well-differentiated thyroid cancer patients.

## 2. Materials and Methods

### 2.1. Bioinformatic Selection of MiRNAs

Transcriptomic signatures of 495 thyroid cancer patients were retrieved from the Genomic Data Commons (GDC) data portal for the Cancer Genome Atlas thyroid cancer dataset (TCGA-THCA) (https://www.cancer.gov/about-nci/organization/ccg/research/structural-genomics/tcga) (accessed on 15 March 2021), and 1035 miRNAs from miRNA-seq were included. Clinical, pathological, and molecular information was obtained from cBioPortal for Cancer Genomics (https://www.cbioportal.org) (accessed on 15 March 2021) and FireBrowse (http://www.firebrowse.org/) (accessed on 15 March 2021). Outcomes of interest were disease recurrence and/or progression. Patients with incomplete recurrence data or unmatched miRNA samples were excluded. Ultimately, 448 non-recurrent and 47 recurrent cancer patients were included. We classified TCGA-THCA cohorts according to the updated 2015 ATA risk stratification for structural disease recurrence into low (≤5%), intermediate (5–20%), or high-risk (≥20%) groups and reported the percentage of the expected risk of recurrence as a quantitative score [8]. A systematic search was performed in the Gene Expression Omnibus (GEO) database (www.ncbi.nlm.nih.gov/geo/) (accessed on 15 March 2021), an online public functional genomics data repository for high-throughput datasets.

Next, we identified all the predicted and experimentally validated miRNAs significantly targeting the thyroid cancer KEGG pathway (KEGG ID: hsa05216) using the DIANA-miRPath v.3.0 (http://www.microrna.gr/miRPathv3) (accessed on 15 March 2021), an miRNA pathway analysis online server [33]. We used the reverse search module at *p*-value < 0.05. Meta-profiling of thyroid cancer miRNAs collected from high-throughput experiments were retrieved from dbDEMC (https://www.biosino.org/dbDEMC/index) (accessed on 15 March 2021), a database of differentially expressed miRNAs in human cancers [34]. Different types of experiments were included, namely comparisons between cancer vs. normal, cancer subtypes, cancer outcome, and blood samples. VENNY v2.1 (https://bioinfogp.cnb.csic.es/tools/venny/) (accessed on 15 March 2021) was utilized to identify the common and exclusive miRNAs for each group. Pathway enrichment analysis of commonly deregulated miRNAs (DEmiR) was performed in DIANA-miRPath v.3.0 using hypergeometric distribution using Fisher’s exact test and a *p*-value threshold = 0.00010.

### 2.2. Study Population and Propensity Score-Matched Groups

**Figure 1:**
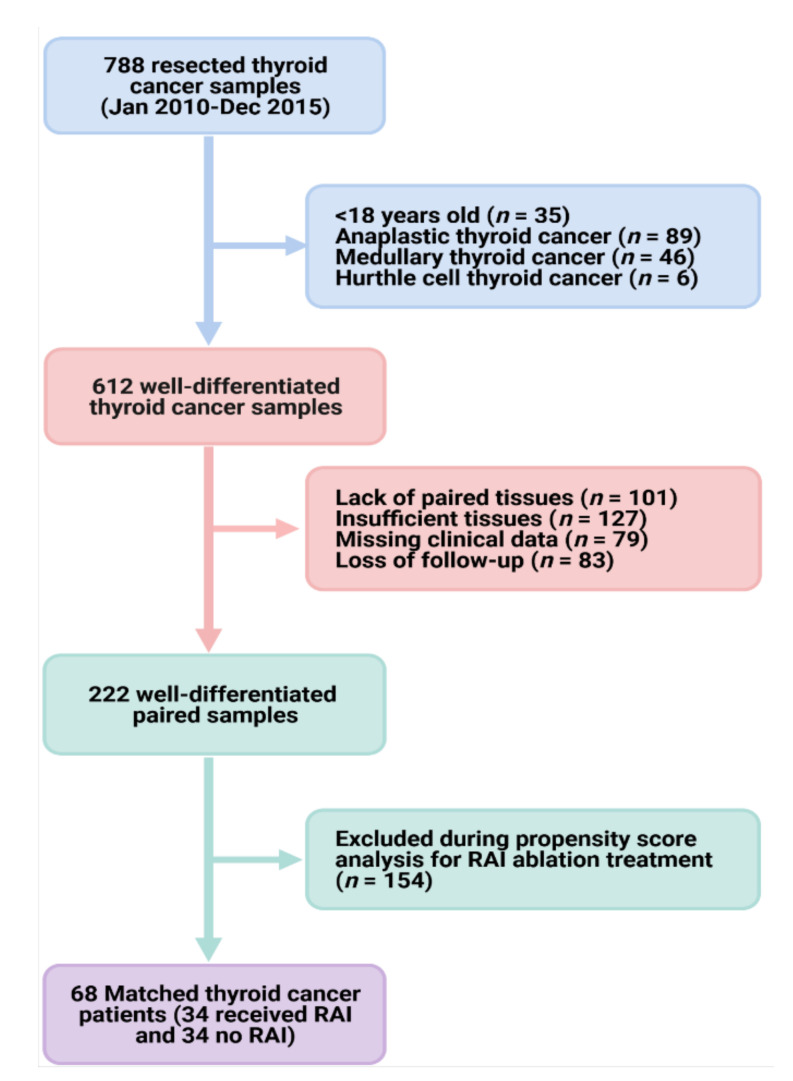
Workflow for patient selection. ATA: American Thyroid Association. We reviewed 788 thyroid cancer samples for patients who underwent subtotal/total thyroidectomy for papillary or follicular thyroid carcinoma. Histopathological analysis was performed, and samples with insufficient tissue available for molecular work or lack of paired non-cancer tissues were excluded. After accounting for selection bias through propensity score analysis, two groups were established with similar general baseline features: (1) 34 paired cancer and non-cancer tissues for patients who underwent thyroidectomy and (2) another 34 paired tissues for those who received RAI following tumor resection.

We retrospectively reviewed 788 patients recruited between January 2010 and December 2015 from Elbayan Pathology Laboratory, Port-Said, Egypt. Samples were collected during thyroidectomy due to thyroid cancer diagnosis. The study population comprised adult cohorts (aged 18 years or older) diagnosed with well-differentiated thyroid cancer (PTC or FTC) according to the International Classification of Oncological Diseases, 4th edition. Patients did not receive any treatment before operative resection. Exclusion criteria included Hürthle cell thyroid carcinoma, poorly differentiated thyroid carcinoma, anaplastic (undifferentiated) carcinoma, medullary thyroid cancer, thyroid lymphoma, thyroid cancer arising from a thyroglossal duct cyst, and thyroid cancer in malignant struma ovarii. Patients with incomplete follow-up or missing data were also excluded. As depicted in the selection process of samples in Figure 1, a total of 222 paired cancer and non-cancer adjacent tissues were eligible. Since the presence of confounders may favor the use of RAI ablation and lead to biased analysis, a confounder-elimination process was conducted using a 1:1 propensity score analysis. Matching yielded two similar datasets of 68 paired samples (cancer and non-cancer tissues) of cohorts who underwent surgical resection of tumor vs. those who had RAI treatment after thyroidectomy. 

### 2.3. Study Variables and Clinical Assessment

From patient records, we obtained demographic and clinicopathological characteristics, treatment strategies, response to therapy, recurrence, and mortality. Demographic variables included age at diagnosis, sex, and year of diagnosis. Disease characteristics included histologic subtype (PTC or FTC), TNM stage (8th edition), presence or absence of multifocal disease, and minor or gross extrathyroidal extension. Patients were classified according to surgery into none, lobectomy, subtotal/near-total thyroidectomy, or total thyroidectomy. 

Data regarding treatment consisted of the extent of surgery, use of radioactive iodine, and use of other treatment modalities (e.g., external beam radiotherapy). Adjuvant RAI therapy was defined as empirical RAI treatment performed after the first reoperation in patients with locoregionally recurrent PTC who initially underwent total thyroidectomy and remnant ablation. Clinical recurrence was defined as the reappearance of pathologically proven malignant tissue and/or the appearance of metastatic lesions such as lung, bone, and/or brain metastases. No clinical evidence of disease (NCED) was defined as the absence of disease, based on physical examination, neck ultrasonography or neck computed tomography (CT) scan, and any other imaging performed during clinical evaluation at the end of follow-up, regardless of serum thyroglobulin concentration.

Disease-free survival (or relapse-free survival) measures the number of people expected to be free from cancer for a particular amount of time to the time of death. Progression-free survival (PFS) was defined as the period from initial treatment to new neoplasm, imaging evidence of disease or disease recurrence, or death. Overall survival refers to the time beginning at the start of treatment and up to the time of death and includes those who survive without any evidence of cancer and those who survive but still have cancer present in their bodies. For individuals who have no tumor relapse, we use the last follow-up time without a recurrence event. 

### 2.4. Tissue Sample Preparation and Histopathological Assessment

Achieved formalin-fixed paraffin-embedded (FFPE) tissues of 68 thyroid cancer and 68 paired non-tumor thyroid samples. Histopathological diagnosis of well-differentiated thyroid cancer was confirmed, and samples were assessed for subtype variant, grading, and staging by two independent pathologists. Laser microdissection (Leica PBI Laser Microdissection model 7) was employed to identify regions of cancer and control tissues in FFPE specimens. Tissues were cut into 4 µm serial sections and stored at 4 °C until use. A 4 µm thick section was used for hematoxylin and Eosin (H&E) staining, and 3–4 sections in Eppendorf tubes were utilized for downstream qRT-PCR experiments. 

### 2.5. RNA Extraction and MicroRNA Quantification

Total RNA, including small RNAs, was purified from FFPE samples by xylene deparaffinization and Qiagen miRNeasy FFPE Isolation kit (Qiagen, Hilden, Germany; Catalog number 217504) following the manufacturer’s protocol. Nucleic acid concentration and purity were determined using the Nanodrop ND-1000 spectrophotometer (NanoDrop Tech. Inc., Wilmington, DE, USA) using the wavelength-dependent extinction coefficient of 33 [35,36]. Samples were stored in aliquots at −80 °C until analysis. RNA (10 ng) was converted to complementary DNA (cDNA) using TaqMan MiRNA Reverse Transcription (RT) kit (P/N 4366596); Thermo Fisher, CA, USA), and the 5× of specific stem-loop primers or endogenous control primers for normalization were used separately. The three miRNAs TaqMan assays are depicted in Table S1. Reverse transcription (RT) was carried out in a T-Professional Basic, Biometra PCR system (Biometra, Goettingen, Germany). Each studied miRNA was specifically converted to complementary DNA using the “TaqMan MiRNA RT kit (P/N 4366596; Applied Biosystems, Foster City, CA, USA)” with 5× miRNA-specific stem-loop primers at the following amplification conditions: 16 °C for 30 min, 42 °C for 30 min, and 85 °C for 5 min, then held at 4 °C. Successful removal of DNA contaminants was confirmed using no-reverse transcription controls of representative samples. qRT-PCR reactions were conducted following the “Minimum Information for publication of quantitative real-time PCR experiments (MIQE)” guidelines [37]. For each specified miRNA quantification, the final volume reaction of 20 µL included 1.33 µL RT product for the specified miR, 2× TaqMan Universal PCR master mix with UNG (Applied Biosystems, P/N 4440043) and 1 µL 20× TaqMan small RNA assay or small nuclear RNA U6 (RNU6B) (assay ID 001093). Three other endogenous control assays (i.e., RNU48, let-7a, and miR-16) were tested for data normalization based on the recent recommendation related to qRT-PCR for miRNAs and endogenous control assessment in PTC archived specimens [38]. Given the consistency and stability of RNU6B across samples, it was applied for data normalization. The PCR was performed in StepOne Real-time PCR system (Applied Biosystems) and incubated as follows: 95 °C for 10 min, followed by 45 cycles of 92 °C for 15 s and 60 °C for 1 min. Reactions were run in triplicate, and standard deviation >2.0 was set as an outlier. Appropriate controls were included in each run [39].

### 2.6. Statistical Analysis

The estimated power of the present study is 96% for a total of 68 matched paired TC samples, medium effect size = 0.5, and alpha error probability = 0.05, using G*Power version 3.1.9.2. With threshold cycle values acquired from the ABI 7900 HT SDS version 2.0.1 software (Applied Biosystems; adjusted settings at automatic baseline and threshold at 0.15), the relative miRNA expression levels were determined by the LIVAK method (2^-ddCq^), where ddC_q_ (delta delta quantitative cycle) = (C_q microRNA of interest_ − C_q endogenous control_)_cancer tissue_ − (C_q microRNA of interest_ − C_q endogenous control_)_non-cancer tissue_ [40]. Wilcoxon matched-pairs signed-rank test was used for comparison of miRNA expression between cancer vs. normal tissues. Data are reported as the median and interquartile range (IQR) and plotted in box plots. Co-expression was estimated through Spearman’s correlation test and plotted in a correlation matrix.

To test the prognostic value of miRNAs, overall and subgroup analysis of all patients (35 progressed vs. 33 indolent courses) and patients who received RAI treatment (20 progressed vs. 14 indolent courses) were performed. Mann–Whitney U or Kruskal–Wallis tests were performed for the comparison between progressed and indolent groups. Receiver operating characteristic (ROC) analysis was performed to test the predictive accuracy of the miRNAs, and the Youden statistic was used to select the best cutoff in discriminating patients’ progression following RAI using the pROC R package [41]. MiRNAs with area under the curve (AUC) greater than 0.75 and *p* < 0.05 were set to be significant. DeLong test was applied using MedCalc (www.medcalc.org/) (accessed on 10 May 2021) to compare the AUC of multiple markers [42]. Accuracy measures including sensitivity, specificity, positive and negative predictive value, and likelihood ratios were calculated, and pooled weighted estimates of the significant miRNAs were determined using Meta-DiSc v.1.4 [43] software for meta-analysis of test accuracy data. 

Data exploration by principal component analysis was performed using psych, factoextra, and FactoMineR R packages. Univariate and multivariate Cox regression revealed that the multi-miRNAs signature plays a functional role independent of clinicopathological characteristics. Hazard ratio and 95% confidence interval (CI) were estimated. Patients were then divided into low- and high-risk groups by the median expression of a specific risk score formula for predicting tumor progression. The risk score for each patient was calculated based on a linear combination of the miRNA expression level weighted by the regression coefficient derived from the multivariate Cox regression, as follows:

$$Risk\;score=\overset{n}{\underset{i-1}{\sum }}COei\times EV_{i}$$

In this formula, *n* represents the number of miRNAs, *Coie* indicates the coefficient of every miRNA in the result of multivariate Cox regression analysis, and *EV_i_* represents the expression level of the miRNA [44,45].

The ability of the miRNA risk score to accurately predict progression events was assessed using Harrell’s concordance index (C-statistic) and Brier score [45]. The Wilcoxon rank-sum test compared the c-statistic and Brier score for each of the miRNAs. A C-statistic of 1.0 represents ideal discrimination, indicating the model is ideal for predicting with a greater probability a patient experiencing an event compared with a patient who does not. Alternatively, a C-statistic of 0.5 indicates that the model is no better at classifying outcomes than random chance. As a priori, we set a C-statistic > 0.8 to indicate excellent discrimination, between 0.7 and 0.8 to indicate reasonable or good discrimination, and between 0.5 and 0.7 to indicate poor or weak discrimination. Brier score measures the accuracy of probabilistic predictions to a set of mutually exclusive discrete outcomes. Across all items i ∈ 1... N in a set of N predictions, the Brier score (BS) measures the mean squared difference between (a) the predicted probability assigned to the possible outcomes for item i and (b) the actual outcome Oi. The score ranges from zero to one and represents the square of the largest possible difference between a predicted probability and the actual outcome. Therefore, the lower the Brier score is for a set of predictions, the better the predictions are calibrated. Next, the Brier skilled score (BSS) was calculated to compare the performance of forecasts with that of a reference probability, which is the ATA risk score. Values closer to 1.0 indicate a perfect forecast, while values closer to 0 indicate unreliable forecast probabilities. We set a score of <0.1 to indicate predictive precision >90% [46]. Calculations of C-statistic and BS were performed using the DescTools R package with Cstat, Brier score functions, and 1000 Bootstrap replicates; then, BSS was calculated manually using the following calculations:

$$BS=\frac{1}{N}\sum_{t+1}^{N}{\left(f_{t}-O_{t}\right)}^{2}\;and\;BSS=1.0-\frac{{\bar{BS}}^{f}}{{\bar{BS}}^{ref}}$$

*f_t_* is the forecast probability, *O_t_ is* the actual outcome of the event at instance *t* (0 if not happened, 1 if happened), and *N* is the number of forecasting instances or the number of items for which the Brier score is being calculated). *Ref* is the BS of reference gold standard test.

Fagan’s Bayesian nomogram was constructed to plot post-test probability (PP) and likelihood ratios (LR) [47]. It is a graphical tool that allows clinicians to use the results of a diagnostic test to estimate a patient’s probability of having the disease. The calculation formula is as follows: Prior probability = probability/(1–probability). Positive likelihood ratio = sensitivity/(1–specificity). Negative likelihood ratio = (1–sensitivity)/specificity. Posterior probability = prior odds * likelihood ratio. We considered likelihood ratio for a positive test (LR+) of more than 10 to significantly increase the probability of disease (“rule in” disease), and for patients who have a negative result, a very low LR—below 0.1—virtually rules out the chance that a person has the disease.

Patients were categorized into high-risk and low-risk groups based on the cutoff value of miRNA risk score at 0.86. Kaplan–Meier plots were applied to illustrate the relationship between high-risk and low-risk groups and survival using the Survminer R package. Log-Rank (Mantel-Cox), Gehan–Breslow–Wilcoxon, and Tarone–Ware tests were applied to investigate the difference in the two curves at different time points. Univariate and multivariate Cox regression models were employed, and a Cox nomogram was constructed using *regplot* and *survival* R packages. Two-sided *p*-values < 0.05 were regarded as significant. Statistical analysis was carried out using IBM SPSS Statistics for Windows, Version 27.0. (IBM Corp. Armonk, NY), GraphPad prism v9.1.1 software (GraphPad Software, San Diego, CA, USA), and R software version 3.4.2 (R Foundation).

### 2.7. Target Gene Prediction, Functional Enrichment Analysis, and External Validation

For the three significant miRNAs, Qiagen Ingenuity Pathway Analysis (IPA) software was used to construct causal networks using complex omics data retrieved from thousands of published articles and curated publicly available datasets within the context of biological systems. Meta-profiling of the miRNAs in 112 cancer experiments in the GEO database (GSE10259, GSE10694, GSE11016, GSE11163, GSE12105, GSE12933, GSE13030, GSE15008, GSE16025, GSE16456, GSE18392, GSE18509, GSE18546, GSE19387, GSE19945, GSE20077, GSE21036, GSE21362, GSE22058, GSE22216, GSE22420, GSE23022, GSE23739, GSE2399, GSE24390, GSE24508, GSE24996, GSE2564, GSE25820, GSE26245, GSE26323, GSE26595, GSE28090, GSE28700, GSE28955, GSE29135, GSE29491, GSE30454, GSE30656, GSE31277, GSE31377, GSE31568, GSE31629, GSE32232, GSE32678, GSE32957, GSE32960, GSE33232, GSE33332, GSE33568, GSE35412, GSE35834, GSE35982, GSE36681, GSE36682, GSE36802, GSE36915, GSE37053, GSE38167, GSE38389, GSE38419, GSE38781, GSE39486, GSE39678, GSE40345, GSE40355, GSE40525, GSE40744, GSE40807, GSE41032, GSE41321, GSE41369, GSE41655, GSE43796, GSE44124, GSE44899, GSE45238, GSE45264, GSE45604, GSE45666, GSE4589, GSE46188, GSE47582, GSE47841, GSE48137, GSE48267, GSE49246, GSE50505, GSE51853, GSE51908, GSE5244, GSE53992, GSE54397, GSE54492, GSE56183, GSE57370, GSE59856, GSE60978, GSE6188, GSE65071, GSE65819, GSE66274, GSE6636, GSE6857, GSE73487, GSE74190, GSE74562, GSE75630, GSE76260, GSE7828, GSE7842, GSE8126) and 20 TCGA cancer datasets (TCGA-ACC, TCGA-BLCA, TCGA-BRCA, TCGA-CESC, TCGA-CHOL, TCGA-COAD, TCGA-ESCA, TCGA-HNSC, TCGA-KICH, TCGA-KIRC, TCGA-KIRP, TCGA-LIHC, TCGA-LUAD, TCGA-LUSC, TCGA-PAAD, TCGA-PRAD, TCGA-SKCM, TCGA-STAD, TCGA-THCA, TCGA-UCEC) were performed to identify the direction trends of different types of cancer. 

### 2.8. Literature Review

Databases for miRNA-disease associations and miRNA-related interactions were screened in GeneCards (www.genecards.org) (accessed on 10 May 2021) and NCBI (https://www.ncbi.nlm.nih.gov/) (accessed on 10 May 2021). There were 199, 369, and 257 articles for miR-204, miR-221, and miR-222, respectively. Non-cancer publications were excluded, and those with corresponding mature miRNA forms were used for data abstraction.

## 3. Results

### 3.1. TCGA and Microarray Thyroid Cancer Cohorts

A total of 495 thyroid cancer patients (448 non-recurrent and 47 recurrent) in the TCGA were screened. Their mean age was 47.2 ± 15.7 years, 73.1% (*N* = 362) were women, and 66.9% (*N* = 331) were white. Patients in the recurrence group were more likely to be older (*p* = 0.040) and white (*p* = 0.018). Higher prevalence of recurrence was found in patients with tumor stage T3/T4 (*p* = 0.009), distant metastasis (*p* < 0.001), and TERT mutation (*p* = 0.011). The median overall survival was 31.0 months (IQR = 17.4–51.9). Upon screening oncologic and clinicopathologic data of TC patients, we found that none of the recurrent patients received radioactive iodine therapy. A systematic search in the GEO database (up to 14 May 2021) revealed a lack of transcriptomic data following radioactive iodine in thyroid cancer patients. Therefore, we could not identify miRNAs with the predictive role of recurrence following RAI treatment using either TCGA or GEO datasets.

### 3.2. Discovery of Candidate Markers Associated with Progression

DIANA-miRPath v.3.0 revealed 469 miRNAs significantly enriched in the thyroid cancer KEGG pathway (KEGG ID: hsa05216) (Table S2). The dbDEMC database, cancer vs. normal, metastasis vs. non-metastasis, and high-grade vs. low-grade tumors were compared (Table S3). The analysis yielded 367 significant deregulated miRNAs (193 upregulated and 174 downregulated) in cancer vs. normal comparison. Of these, 21 upregulated and 19 downregulated genes were differentially expressed at absolute fold change (FC) greater than 1. As prognostic biomarkers, 349 miRNAs (170 upregulated and 179 downregulated) were significant compared to the advanced tumor stage vs. lower stage. Filtration at FC > 1.0 showed 12 significant upregulated and eight downregulated miRNAs. Of these, six miRNAs were removed as they showed a paradoxical expression profile (Table S4). 

**Figure 2:**
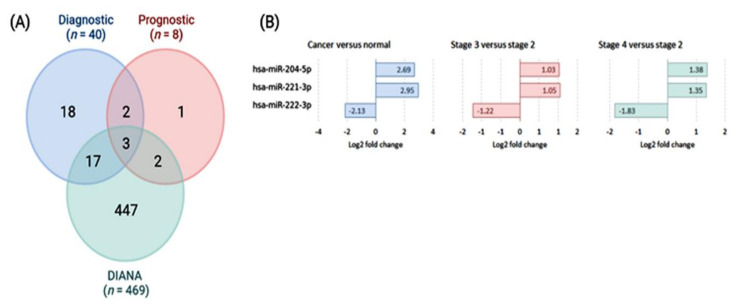
Schemes selection of candidates for poor prognostic miRNAs in thyroid cancer. (**A**) Venn diagram showing the intersection between putative diagnostic and prognostic miRNA biomarkers identified by meta-profiling of transcriptomic signature of high-throughput experiments [data source: dbDEMC (https://www.biosino.org/dbDEMC/index) (accessed on 20 May 2021) and miRNAs targeting genes in thyroid cancer KEGG pathway [data source: DIANA-miRPath v.3.0 (http://www.miRNA.gr/miRPathv2) (accessed on 20 May 2021)]. (**B**) Expression profile of significant miRNAs of thyroid cancer tissues in high-throughput experiments (TCGA and microarray).

The intersection between highly expressed miRNAs (FC > 1.0) with diagnostic and prognostic values and those identified in DIANA-miRPath v.3.0 depicted three common miRNAs (Figure 2A). Both miR-221-3p and miR-222-3p were upregulated in cancer compared to controls and in an advanced stage compared to lower disease stage (Figure 2B). They were highly enriched in cancer-related pathways as pathways in cancer, proteoglycans in cancer, Hippo signaling pathway, p53 signaling pathway, cell cycle, adherens junction, and cancer-specific KEGG pathways. 

### 3.3. Characteristics of Papillary Thyroid Cancer Patients

For the matched cohorts, the mean age at diagnosis was 38.5 years (range 18–80), and 72.1% (*N* = 49) were women. As demonstrated in Table 1, clinical and pathological characteristics of patients who received postoperative RAI matched those who did not. Around 30.9% (*N* = 21) presented with bilateral nodules and 50% (*N* = 34) had T1 tumor size stage. Of the study population, 82.4% (*N* = 56) underwent total/subtotal thyroidectomy and 77.9% (*N* = 53) had neck dissection. After an 8-year follow-up, tumor progression was reported in 51.5% of patients (*N* = 35), including 17 patients (48.6%) with T1a/b tumor stage at the time of diagnosis. Characteristics of thyroid cancer patients who received post-operative radioactive ablation and those who did not are shown in Table 2.

### 3.4. MicroRNA Expression Levels in Thyroid Cancer

**Figure 3:**
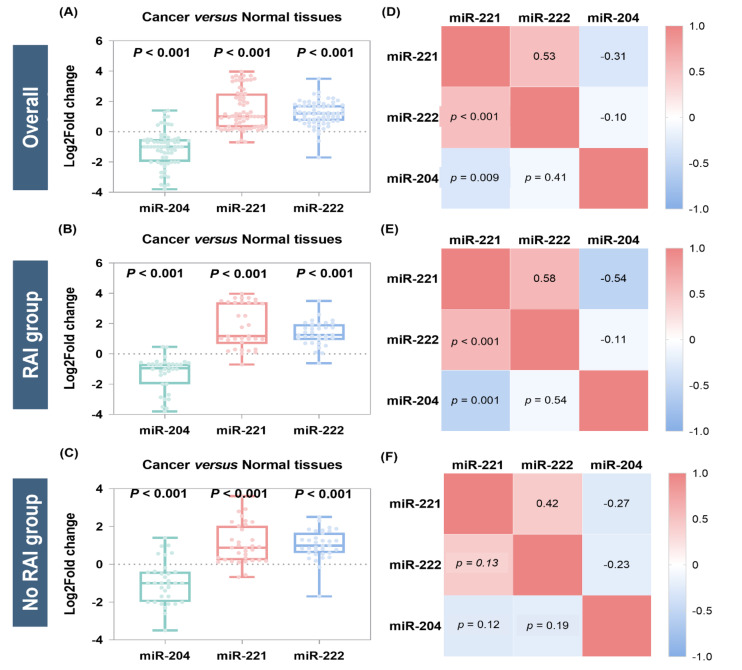
Relative expression of microRNAs in thyroid cancer tissues compared to paired counterparts. The plot represents overall analysis for the 68 patients and subgroup analysis for the 34 RAI and 34 non-RAI groups. (**A**–**C**) Box plots show the median and interquartile range in cancer. Log2 fold change below 0 indicates downregulation, while greater than 0 indicates overexpression compared to normal. Wilcoxon matched-pairs signed-rank test was used for comparison. Significance was set at *p*-value < 0.05. (**D**–**F**) Correlation matrix for gene co-expression. Spearman’s correlation analysis was performed. The correlation coefficient (−1 to 1) is presented in the top right of the matrix, and its equivalent *p*-value is in the bottom left.

Analysis of the whole cohort revealed significant upregulation of miR-221-3p (median = 1.01, IQR = 0.29 to 2.52, *p* < 0.001) and miR-222-3p (median = 1.25, IQR = 0.70 to 1.75, *p* < 0.001) and downregulation of miR-204-5p (median = −1.0, IQR = −2.0 to −0.5, *p* < 0.001) in cancer tissues compared to non-cancer adjacent tissues (Figure 3A). Subgroup analysis in the RAI group also revealed overexpression of miR-221-3p (median = 0.87, IQR = 0.21 to 2.04, *p* < 0.001) and miR-222-3p (median = 0.95, IQR = 0.58 to 1.75, *p* < 0.001), and under expression of miR-204-5p (median = −0.94, IQR = −2.0 to −0.66, *p* < 0.001) (Figure 3B). A similar expression trend was found in the non-RAI group; log fold changes were (median = 0.87, IQR = 0.21–2.04, *p* < 0.001) for miR-221-3p, (median = 0.99, IQR = 0.58–1.67, *p* < 0.001) for miR-222-3p, and (median = −1.0, IQR = −2.0 to −0.39, *p* < 0.001) for miR-204-5p. In comparison, correlation matrices show a positive correlation between miR-221-3p and miR-222-3p (*r* = 0.53, *p* < 0.001) and a negative correlation between miR-221-3p and miR-204-5p (*r* = −0.31, *p* = 0.009) for the overall analysis (Figure 3D). Similar trends were observed in subgroup analysis (Figure 3E,F).

### 3.5. Association of MicroRNAs with Clinical and Pathological Features

There was no significant difference of miRNA expression in thyroid cancer tissues with various demographic and clinical features. However, tissue miR-221-3p (*p* = 0.036) and miR-222-3p (*p* = 0.017) overexpression were associated with lymph node metastasis, and miR-204-5p (*p* = 0.037) downregulation was linked to multifocality in well-differentiated thyroid cancer patients. Notably, miRNAs were not differentially expressed in RAI and non-RAI groups (*p* = 0.50 for miR-204-5p, *p* = 0.33 for miR-221-3p, and *p* = 0.13 for miR-222-3p) (Table 3).

### 3.6. MicroRNA Predictive Performance for Progression Following RAI Treatment

**Figure 4:**
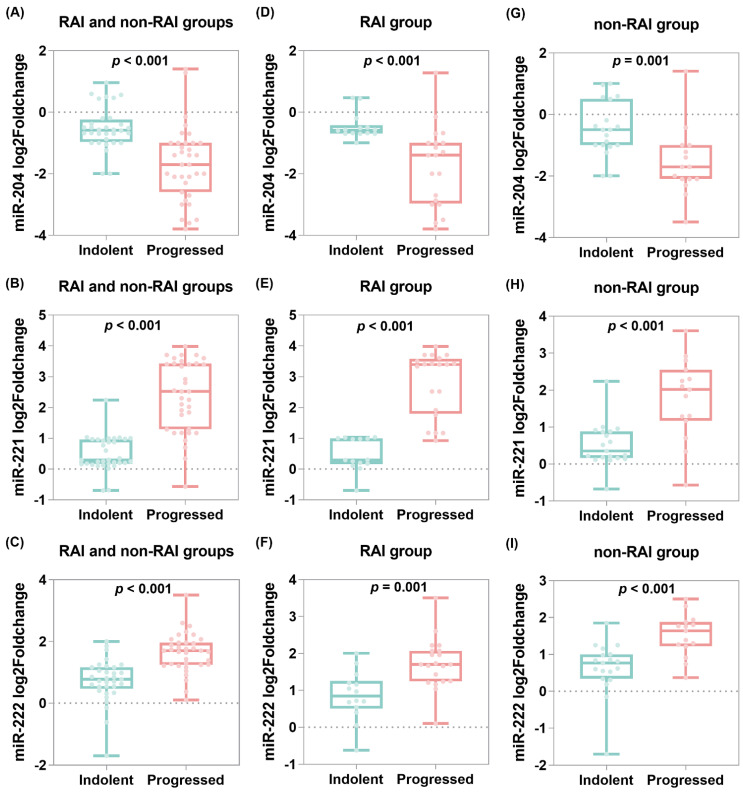
Tissue microRNA expression profile and tumor progression. Plots represent overall analysis for the 68 patients and subgroup analysis for the 34 patients in the RAI group. Mann–Whitney U test was used to compare the expression levels between indolent and progressive samples. Log2 fold change was estimated using the ddCT method. (**A**–**C**) Overall analysis, (**D**–**F**) RAI group, and (**G**–**I**) non-RAI group.

In comparison between indolent and progressive tumors, there was a remarkable downregulation of miR-204 in progressive cases (median = −1.7, IQR = −2.6 to −1.0) compared to indolent specimens (median = −0.58, IQR = −0.9 to −0.24, *p* < 0.001) and overexpression of both miR-221 (median = 2.52, IQR = 1.29–3.43 vs. median = 0.29, IQR = 0.16–0.99, *p* < 0.001) and miR-222 (median = 1.7, IQR = 1.24–1.96 vs. median = 0.77, IQR = 0.46–1.16, *p* < 0.001) (Figure 4A–C). Subgroup analysis of RAI cohorts revealed similar findings for miR-204 cases (median = −1.4, IQR = −2.97 to −0.10 vs. median = −0.58, IQR = −0.7 to −0.42, *p* <0.001), miR-221 (median = 3.39, IQR = 1.8–3.58 vs. median = 0.29, IQR = 0.16–0.99, *p* <0.001), and miR-222 (median = 1.7, IQR = 1.24–2.07 vs. median = 0.84, IQR = 0.50–1.26, *p* < 0.001) (Figure 4D–F). For the non-RAI group, there was also a significant difference between progressive and indolent tumor specimens for miR-204 (median = −1.7, IQR = −2.1 to −1.0 vs. median = −0.5, IQR = −1.0 to 5.0), miR-221 (median = 2.02, IQR = 1.17 to 2.55 vs. median = 0.35, IQR = 0.16 to 0.88), and miR-222 (median = 1.64, IQR = 1.22 to 1.88 vs. median = 0.77, IQR = 0.34 to 1.0) (Figure 4G–I). In contrast, the ATA risk score did not differ significantly between non-progressive and progressive groups (Table 2).

The three miRNAs showed good accuracy to predict tumor progression in overall and subgroup analysis. The AUC of miR-204 was 0.85 (95% CI = 0.75–0.93, *p* < 0.001) and 0.91 (95% CI = 0.75–0.98), miR-221 was 0.93 (95% CI = 0.82–0.97, *p* < 0.001) and 0.97 (95% CI = 0.87–1.0, *p* < 0.001), and miR-222 was 0.85 (95% CI = 0.74–0.92) and 0.83 (95% CI = 0.66–0.94, *p* < 0.001). In contrast, the ATA risk score did not show significant discriminative ability shown in the ROC curve (AUC = 0.613, 95% CI = 0.487–0.729, *p* = 0.06) with high error probability (cost = 0.412) (Table 4). Comparison between the AUC of the three miRNAs showed an insignificant difference between them, highlighting having similar high efficiency following RAI ablation therapy (pairwise comparison: miR-204~miR-221 = 0.24 and 0.31; miR-204~miR-222 = 0.97 and 0.40; and miR-221~miR-222 = 0.19 and 0.06). 

**Figure 5:**
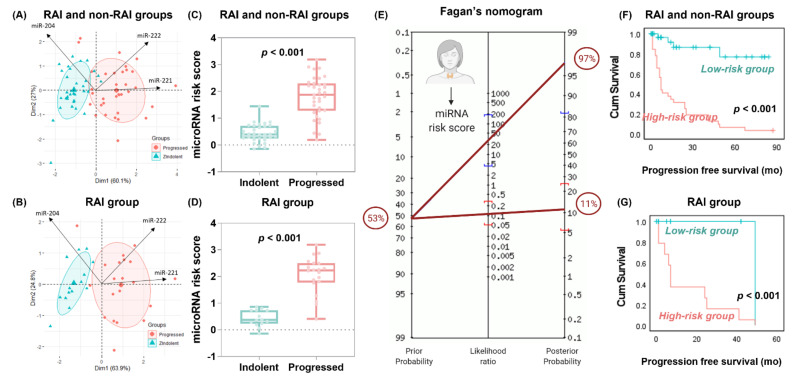
Predictive role of microRNA risk score. The risk score was calculated using the coefficient of the multivariate Cox regression analysis, using the following formula: (−0.260 × expression level of miR-204) + (0.523 × expression level of miR-221) + (0.75 × expression level of miR-222). (**A**,**B**) Principal component analysis for data exploration. Data are presented across X and Y axes. Arrows for each variable point in the direction of increasing values of that variable. There was a clear demarcation between the two groups (indolent and aggressive tumors), with higher levels (long arrows) of miR-221 and miR-222 in progressive tumors, while the miR-204 level was higher in the direction of non-progressive samples. The microRNA discrimination ability performed slightly better in patients following radioactive iodine. (**C**,**D**) Relative expression of microRNAs in progressed samples compared to indolent. Box plots show the median and interquartile range in cancer. Mann–Whitney U test was used. *p*-value was set significant at <0.05. (**E**) Fagan’s Bayesian nomogram for forecasting probabilities. In this nomogram, a straight line drawn from a patient’s pre-test probability of disease (left axis) through the likelihood ratio of the test (middle axis) will intersect with the post-test probability of disease (right axis). Prior probability (odds): 53 ± 1.1% and LR+: 28 (95% CI = 4.12–196), LR-: 0.11 (95% CI = 0.05–0.29) yielded a post-test probability (odds) of 97 ± 31.5% for positive test and 11 ± 0.1% for negative test. (**F**,**G**) Kaplan–Meier curves for survival analysis. Log-rank test was used to compare high-risk and low-risk groups categorized based on the microRNA risk score above and below 0.86.

In the PCA plot, exploratory data analysis showed clear discrimination between tumor specimens who remained indolent and those that progressed, with higher levels (long arrows) of miR-221 and miR-222 in progressive tumors, while the miR-204 level was higher in indolent samples. The miRNA discrimination ability performed slightly better in patients following radioactive iodine (Figure 5A,B). Univariate and multivariate Cox regression analyses showed miR-204, miR-221, and miR-222 as independent risk factors for tumor progression (Table 5).

### 3.7. Prognostic Value of MicroRNA Risk Score and Nomogram Construction

The miRNA risk score was generated as follows: (−0.260 × expression level of miR-204) + (0.523 × expression level of miR-221) + (0.75 × expression level of miR-222). The risk score was higher in cases that developed tumor progression postoperatively (median = 1.87, IQR = 1.28–2.3 vs. median = 0.39, IQR = 0.24–0.71, *p* < 0.001). Similarly, in the RAI group, risk score was significantly higher in progressive tumors (median = 2.23, IQR = 1.77–2.5, *p* < 0.001 vs. median = 0.37, IQR = 0.37, IQR = 0.22–0.73, *p* < 0.001) (Figure 5C–D). At the cutoff value of > 0.86, the AUC was 0.944 (95% CI = 0.85–0.98) for overall population and 0.979 (95% CI = 0.87–1.00) for the RAI group with 88.6% (95% bootstrap CI = 73.3–96.8%) sensitivity, 97% (95% CI = 84.2–99.9%) specificity, 96.9% (95% CI = 81.8–99.5%) positive predictive value, and 88.9% (95% CI = 76.0–95.3%) negative predictive value. Across the 68 patients, only four cases (5.9%) were false negative and one case (1.5%) false positive at the miRNA risk score of 0.86. Fagan’s Bayesian nomogram shows that posterior probabilities for tumor progression increased from 53% to 97% (95% CI = 82–100%) if the miRNA risk score exceeded 0.86 and decreased from 53% to 11% (95% CI = 5–25%) if the score was below 0.86 (Figure 5F).

Compared to the low-risk group, the high-risk group had a threefold increased progression risk (HR = 2.71, 95% CI = 1.86–3.96, *p* < 0.001). Kaplan–Meier survival curves showed shorter survival times in the high-risk group of patients. In the overall analysis, patients with risk score >0.86 had disease-free survival (17.3 months, 95% CI = 10.06–24.55) compared to those with lower risk score (70.79 months, 95% CI = 59.12–82.45, *p* < 0.001). Similarly, subgroup analysis of patients who received RAI ablation treatment showed lower survival in the high-risk group (14.7 months, 95% CI = 7.82–21.7) compared to the low-risk group (49.0 months, *p* < 0.001) (Figure 5D,E). The risk score performed best for predicting progression in the whole cohort (C-statistics = 0.943, Brier = 0.083) and RAI subgroup (C-statistic = 0.978, Brier = 0.049). However, the scores did not discriminate well for other pathological features and clinical outcomes (Table 6).

**Figure 6:**
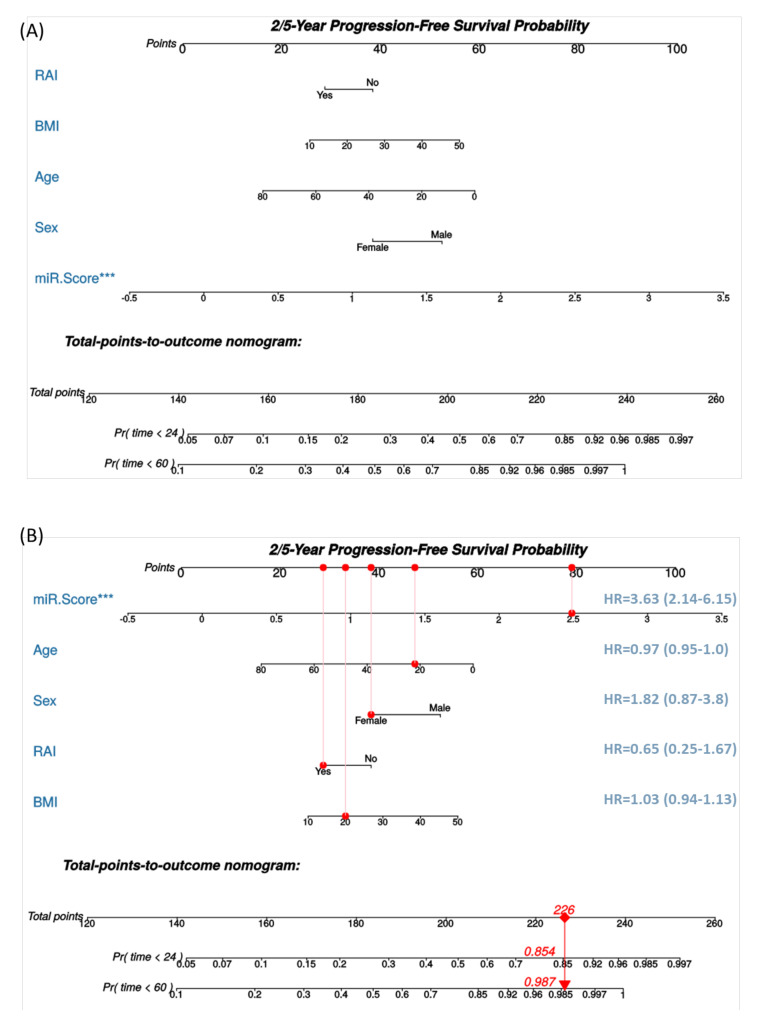
A nomogram for thyroid cancer prognosis. (**A**) Nomogram is predicting 2- and 5-year progression-free survival. The current nomogram is derived from well-differentiated thyroid cancer cohorts who underwent surgery at a single center. The outcome measured was a post-operative progression. Cox proportional hazard model was applied. (**B**) Example for using the nomogram. Assumed having a 20-year-old female patient with a body mass index (BMI) of 20 Kg/m^2^, whose tissue microRNA risk score was high at 2.5, and received radioactive iodine (RAI) ablation. Each variable will be scored on its points scale. The scores for all variables are then added to obtain the total score, and a vertical line is drawn from the total-points row to estimate the probability of survival rates within 2 years and 5 years. *** indicates *p* < 0.001.

For clinical implementation by physicians, we constructed a Cox nomogram to predict progression-free survival within two- and five-years following diagnosis using microRNA risk score, radioactive ablation treatment, and demographic features (Figure 6A). An example for the interpretation of nomogram and cox regression results is shown in Figure 6B. The concordance index was 0.805 ± 0.037, indicating that the model has good discrimination ability.

### 3.8. Meta-Profiling Signature of MicroRNAs in Cancer

Analysis of 132 high-throughput experiments demonstrated the deregulation of miR-204-5p, miR-221-3p, and miR-222-3p in various types of cancer. The expression level of miR-204-5p was the lowest in bladder cancer (GSE40355, FC = −8.42), renal cancer (GSE11016, FC = −7.59), breast cancer (GSE45666, FC = −4.75), cervical cancer (TCGA_CESC, FC = −4.39), and melanoma (GSE24996, FC = −4.03). In addition, downregulation of miR-204-5p was found in metastatic melanoma (GSE18509, FC = −3.9) and prostate cancer (GSE21036, FC = −2.16) (Tables S5 and S6). The miR-221-3p overexpression was mostly noted in hepatocellular carcinoma (GSE20077, FC = 4.85), ovarian cancer (GSE65819, FC = 4.3), renal cancer (TCGA_KICH, FC = 4.0), pancreatic cancer (GSE28955, FC = 3.18), glioblastoma (GSE13030, FC = 3.16), and thyroid cancer (TCGA_THCA, FC = 2.95). In addition, elevated levels of miR-221-3p were observed in the circulation of patients with melanoma (GSE31568, FC = 1.24) and cohorts with advanced tumor grade in thyroid cancer (TCGA_THCA, FC = 1.35) and breast cancer (GSE22216, FC = 0.3) (Table S7). The miR-222-3p elevated levels were found in lymphoma (GSE12933, FC = 5.44), kidney cancer (GSE11016, FC = 4.13), pancreatic carcinoma (GSE28955, FC = 3.82), biliary tract cancer (GSE53992, FC = 3.3), and thyroid cancer (TCGA_THCA, FC = 2.69). Circulatory upregulation was observed in sarcoma (GSE65071, FC = 1.57) and retinoblastoma (GSE41321, FC = 1.23). The expression profile was significantly higher in advanced disease stage in thyroid cancer (TCGA_THCA, FC = 1.38) and renal cancer (TCGA_KIRP, FC = 0.68) (Table S8). Literature screening results are depicted in Table 7.

### 3.9. Discovery of the Regulatory Network

**Figure 7:**
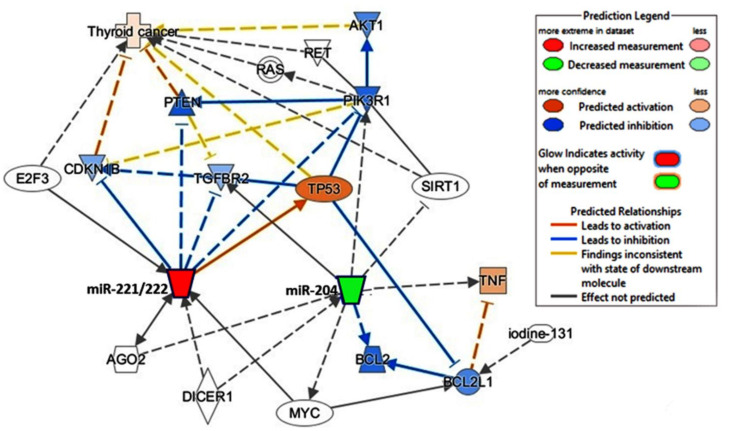
Causal network analysis for the predicted effect of microRNAs deregulation on thyroid cancer disease. Overexpressed miR-221 and miR-222 (red node) and downregulation of miR-204 (green node) are predicted to activate (orange) and inhibit (blue) genes, which lead to activation of thyroid cancer KEGG pathway. Data source: Knowledge base Ingenuity Pathway Analysis (IPA, Qiagen Inc., https://www.qiagenbioinformatics.com/products/ingenuity-pathway-analysis) (accessed on 21 May 2021).

The predicted regulatory network showed that upregulated miR-221 and miR-222 and downregulated miR-204 leads to a subsequent cascade promoting thyroid cancer pathway (Figure 7). A systematic review demonstrated multiple deregulated signaling pathways and mechanisms leading to tumor development (Table S9) [28,58,59,60,61,62,63,64,65,66,67,68,69,70,71,72,73,74,75,76,77,78,79,80,81,82,83,84,85,86,87,88,89,90,91,92,93,94,95,96,97,98,99,100].

## 4. Discussion

Despite evidence of the prominent diagnostic, prognostic, and predictive role of miRNAs in DTC, accurate prediction of progression is highly challenging in TC patients. Without a convenient marker to guide an informed decision between radical treatment and continued observation for PTC, there is an urgent need to identify the biological behavior of such tumors. This work serves as an initial proof-of-principle study that the triple miRNA biomarkers (miR-221, -222, and -204) can predict tumor progression following radioactive iodine ablation in well-differentiated TC patients.

The propensity score matching analysis approach was followed in the current study for specimen selection to allow data matching in general baseline factors and establish two similar datasets for investigating the expression of the selected miRNAs. There was no significant difference in microRNA expression in RAI and non-RAI groups, with consistent deregulation of our microRNA panel. Results showed miR-221 and miR-222 upregulation and miR-204 downregulation to exhibit good predictive accuracy for recurrence, even following RAI therapy. Radiation oncology-associated miRNAs were found to modulate cell death and proliferation after irradiation [101]. However, the molecular basis of gene regulation in cells exposed to radioactive iodine is not fully understood. Some molecular markers as the presence of BRAF^V600E^ and TERT promoter mutations strongly indicate loss of iodine uptake rate and impairment of the iodide-metabolizing machinery [102]. However, around half of PTC tumors not harboring these mutations are non-RAI avid, highlighting a complicated mechanism underlying tumor recurrence/persistence following RAI ablation.

Several miRNAs, such as miR-221/miR-222, form clusters and exert coordinated expression and function [103]. Overexpression of miR-221 and miR-222 were observed in classic PTC [104,105,106,107,108], the follicular variant of PTC [108], conventional type of FTC [106], poorly differentiated TC [106], anaplastic TC [106,109], and the FTC oncocytic type for miR-221 [106]. They showed high accuracy in TC prediction preoperatively using fine-needle aspiration biopsies from thyroid nodules and surgical samples [105,110,111,112]. miR-221 upregulation is a premalignant change in PTC, and its upregulation was strongly observed not only in tumor sections but also in 3/15 adjacent non-cancer tissues of cancer paired samples [104], confirming its pro-oncogenic role in TC. Indeed, upregulation of miR-221 was also significantly associated with an increased risk of recurrence [113].

Accumulating evidence shows that miR-221 and miR-222 in TC can downregulate tyrosine kinase (KIT) receptors [104] and cyclin-dependent kinase inhibitor 1B (CDKN1B; p27/Kip1) protein [109], among others (Figure 7). Furthermore, miR-221/miR-222 can respond to cellular stresses, such as radiation, by activating the transcriptional factor nuclear factor kappa B (NFκB) and activator protein-1 (AP-1) promoters [114]. High-mobility group box 1 protein (HMGB1)-dependent miR221/222 overexpression in PTC could interfere with the PTEN-dependent cell cycle regulation and hence is associated with an elevated malignancy score in terms of cell growth and motility [103].

The tumor-suppressive function of miR-204 has been reported in multiple cancers, including hepatocellular carcinoma [115], glioma [116], and clear cell renal cell carcinoma [117]. In addition, its downregulation in TC samples and cell lines has been identified previously [27,28] and found to target the high-mobility group AT-hook 2 (HMGA2) [27] and the insulin-like growth factor-binding protein 5 (IGFBP5) transcripts [28]. MiR-204 upregulation decreased cyclin D1/Ki67 expression and increased P21 expression with subsequent TC cell proliferation inhibition [27].

Using loss-of-function assays, Li et al. recently confirmed that the oncogenic long non-coding RNA LINC00514 could promote proliferation/migration and invasion and suppress apoptosis of PTC via miR-204-3p/CDC23 axis [118]. As part of the urothelial carcinoma-associated 1 (UCA1)/miR-204/bromodomain containing 4 (BRD4) axis, miR-204 plays an essential role in PTC cell proliferation/invasion and shows potential for therapeutic applications in PTC patients [119]. Interestingly, the latter investigators found that the long non-coding RNA UCA1 and miR-204 could negatively regulate each other, and the UCA1 may compete with BRD4 for miR-204 binding results in downregulating miR-204, promoting BRD4 expression and affecting PTC progression. Collectively, these findings support the prognostic value of miR-204 downregulation in the proposed model in our samples. 

Although the present study has a modest sample size due to strict inclusion/exclusion criteria for selecting well-differentiated thyroid cancer patients subjected to postoperative radioactive iodine ablation, it was large enough to achieve significance in the predictive power of each cohort. In addition, using FFPE samples may seem disadvantageous from the point of view of some investigators, but miRNA expression signatures can be obtained with relative ease and stability using quantitative RT-PCR in tumor biopsy tissue [110,120]. Thus, the proposed prognostic risk score can be calculated as a part of the pathology workup.

Our novel three miRNAs panel and nomogram could accurately identify tumors that are likely to acquire more progressive behavior in PTC patients so that improved management strategies can be developed, avoiding unnecessary tissue biopsy and surgical intervention. Assessment of the prognostic value of this panel in a large-scale multicenter prospective setting is recommended to support the clinical utility and validity of this model. Evaluation of the identified panel with other treatment modalities such as irradiation and a combination of neck dissection and postoperative radiotherapy is warranted. 

## 5. Conclusions

Our predictive panel/nomogram can define which cancers will have an aggressive phenotype, providing a new paradigm for managing patients diagnosed with localized low-risk DTC. Not only would this have an enormous positive impact on our ability to longitudinally monitor thyroid cancer for evidence of disease progression in a prospective clinical trial, but identifying the signature specific for tumor aggressiveness would unravel new pathophysiological mechanisms and open new horizons to tackle cancer with non-invasive diagnostics and innovative new miRNA-based therapeutics.

## Figures and Tables

**Table 1:** Baseline characteristics of propensity score-matched cohorts (*N* = 68).

Patient Characteristics	Levels	Total (*N* = 68)	No RAI (*N* = 34)	RAI (*N* = 34)	*p*-Value
Demographic data					
Age, years	Median (IQR)	27 (22–43)	29 (24–45)	23 (22–41.5)	0.09
	<55 y	55 (80.9)	25 (73.5)	30 (88.2)	0.21
	≥55 y	13 (19.1)	9 (26.5)	4 (11.8)	
Sex	Female	49 (72.1)	23 (67.6)	26 (76.5)	0.59
	Male	19 (27.9)	11 (32.4)	8 (23.5)	
BMI, Kg/m^2^	Mean ± SD	26.8 ± 5.3	25.5 ± 1.39	27.8 ± 6.9	0.06
Pathological assessment					
Laterality	Unilateral	47 (69.1)	27 (79.4)	20 (58.8)	0.11
	Bilateral	21 (30.9)	7 (20.6)	14 (41.2)	
Histological variant	Conventional	30 (44.1)	10 (29.4)	20 (58.8)	0.18
	Micropapillary	20 (29.4)	16 (47.1)	4 (11.8)	
	Follicular	7 (10.3)	3 (8.8)	4 (11.8)	
	Follicular + Oncocytic	9 (13.2)	3 (8.8)	6 (17.6)	
	Oncocytic	1 (1.5)	1 (2.9)	0 (0)	
	Tall cell	30 (44.1)	10 (29.4)	20 (58.8)	
Pathology Stage	Stage Ia	47 (69.1)	25 (73.5)	22 (64.7)	0.18
	Stage II	13 (19.1)	5 (14.7)	8 (23.5)	
	Stage III	3 (4.4)	3 (8.8)	0 (0)	
	Stage IVA	3 (4.4)	1 (2.9)	2 (5.9)	
	Stage IVB	2 (2.9)	0 (0)	2 (5.9)	
Max Tumor size, cm	Median (IQR)	2.5 (2.0–3.5)	2.5 (2.0–3.0)	2.7 (1.6–4.6)	0.71
T stage	T1a	11 (16.2)	5 (14.7)	6 (17.6)	0.22
	T1b	23 (33.8)	15 (44.1)	8 (23.5)	
	T2	16 (23.5)	6 (17.6)	10 (29.4)	
	T3a	10 (14.7)	6 (17.6)	4 (11.8)	
	T3b	8 (11.8)	2 (5.9)	6 (17.6)	
N stage	N0	36 (52.9)	20 (58.8)	16 (47.1)	0.46
	N1	32 (47.1)	14 (41.2)	18 (52.9)	
M stage	M0	54 (79.4)	30 (88.2)	24 (70.6)	0.13
	M1	14 (20.6)	4 (11.8)	10 (29.4)	
Focality	Unifocal	29 (42.6)	15 (44.1)	14 (41.2)	0.81
	Multifocal	39 (57.4)	19 (55.9)	20 (58.8)	
ETE	Negative	68 (100)	34 (100)	34 (100)	NA
LVI	Negative	68 (100)	34 (100)	34 (100)	NA
Perineural invasion	Negative	64 (94.1)	30 (88.2)	34 (100)	0.11
	Positive	4 (5.9)	4 (11.8)	0 (0)	
Extranodal extension	Negative	66 (97.1)	34 (100)	32 (94.1)	0.49
	Positive	2 (2.9)	0 (0)	2 (5.9)	
Intervention					
Thyroidectomy	Unilateral	12 (17.6)	8 (23.5)	4 (11.8)	0.34
	Total/subtotal	56 (82.4)	26 (76.5)	30 (88.2)	
Neck dissection	Negative	15 (22.1)	9 (26.5)	6 (17.6)	0.56
	Positive	53 (77.9)	25 (73.5)	28 (82.4)	
Follow-up					
Progression	Negative	33 (48.5)	19 (55.9)	14 (41.2)	0.33
	Positive	35 (51.5)	15 (44.1)	20 (58.8)	
Mortality	Survived	63 (92.6)	31 (91.2)	32 (94.1)	0.64
	Died	5 (7.4)	3 (8.8)	2 (5.9)	

Data are represented as frequency (percentage), mean ± standard deviation (SD), or median and interquartile range (IQR). BMI: body mass index; ETE: Extrathyroidal extension; LVI: Lymphovascular invasion; NA: Not applicable; progression: included recurrence, relapse, and distant metastasis. Two-sided Chi-square, Student’s *t*, and Mann–Whitney *U* tests were used. Statistical significance was set at *p*-value < 0.05.

**Table 2:** Comparison between thyroid cancer patients according to their progression, stratified by radioactive iodine treatment.

Patient Characteristics	Levels	Indolent (*N* = 19)	Progression (*N* = 15)	*p*-Value	Indolent (*N* = 14)	Progression (*N* = 20)	*p*-Value
Demographic data							
Age, years	<55 y	13 (68.4)	12 (80)	0.69	14 (100)	16 (80)	0.12
	≥55 y	6 (31.6)	3 (20)		0 (0)	4 (20)	
Sex	Female	15 (78.9)	8 (53.3)	0.45	12 (85.7)	14 (70)	0.42
	Male	4 (21.1)	7 (46.7)		2 (14.3)	6 (30)	
Pathological assessment							
Laterality	Unilateral	15 (78.9)	12 (80)	0.94	6 (42.9)	14 (70)	0.16
	Bilateral	4 (21.1)	3 (20)		8 (57.1)	6 (30)	
Pathology Stage	Stage I	16 (84.2)	9 (60)	**0.047**	10 (71.4)	12 (60)	0.35
	Stage II	0 (0)	5 (33.3)		4 (28.6)	4 (20)	
	Stage III	2 (10.5)	1 (6.7)		-	-	
	Stage IVA	1 (5.3)	0 (0)		0 (0)	2 (10)	
	Stage IVB	-	-		0 (0)	2 (10)	
Max Tumor size, cm	Median (IQR)	2 (2–3.5)	2.5 (2–3.0)	1.00	2.5 (1.8–5.0)	3.0 (1.5–3.5)	0.74
T stage	T1a	3 (15.8)	2 (13.3)	0.30	2 (14.3)	4 (20)	0.59
	T1b	10 (52.6)	5 (33.3)		2 (14.3)	6 (30)	
	T2	2 (10.5)	4 (26.7)		6 (42.9)	4 (20)	
	T3a	4 (21.1)	2 (13.3)		2 (14.3)	2 (10)	
	T3b	0 (0)	2 (13.3)		2 (14.3)	4 (20)	
N stage	N0	12 (63.2)	8 (53.3)	0.72	8 (57.1)	8 (40)	0.48
	N1	7 (36.8)	7 (46.7)		6 (42.9)	12 (60)	
M stage	M0	19 (100)	11 (73.3)	**0.029**	10 (71.4)	14 (70)	0.61
	M1	0 (0)	4 (26.7)		4 (28.6)	6 (30)	
Focality	Unifocal	12 (63.2)	3 (20)	**0.017**	4 (28.6)	10 (50)	0.29
	Multifocal	7 (36.8)	12 (80)		10 (71.4)	10 (50)	
ATA risk score	Median (IQR)	10 (10–10)	10 (10–40)	0.08	20 (1040)	10 (10–40)	0.90
Intervention							
Thyroidectomy	Unilateral	2 (9.1)	6 (50)	**0.044**	2 (14.3)	2 (10)	0.55
	Total/subtotal	17 (89.5)	9 (60)		12 (85.7)	18 (90)	
Neck dissection	Negative	6 (31.6)	3 (20)	0.69	2 (14.3)	4 (20)	0.66
	Positive	13 (68.4)	12 (80)		12 (85.7)	16 (80)	
Follow-up							
Mortality	Survived	19 (100)	12 (80)	0.07	14 (100)	18 (90)	0.50
	Died	0 (0)	3 (20)		0 (0)	2 (10)	
Metastasis-free survival, months	Median (IQR)	56 (40–81)	10 (1–64)	**0.013**	7.0 (0.75–40.7)	4.0 (0.01–7.0)	0.69
Progression-free survival, months	Median (IQR)	53 (18–80)	8 (4–16)	**<0.001**	6.0 (1.0–7.0)	1.0 (0.75–5.5)	0.12
Overall survival, months	Median (IQR)	68 (41.5–82.5)	64 (6–75)0.53	0.31	7.0 (1.0–40.7)	70 (3.0–48.0)	0.21

Data are represented as frequency (percentage), or median and interquartile range (IQR). BMI: body mass index. Two-sided Chi-square and Mann–Whitney *U* tests were used. Significant *p*-values < 0.05 are shown in the table in bold.

**Table 3:** Association of microRNA expression levels with demographic and clinicopathological characteristics (N = 68).

Characteristics	Levels	miR-204-5p	miR-221-3p	miR-222-3p
Demographic data				
Age, years	≥55 y vs. <55 y	0.57	0.67	0.77
Sex	Male vs. female	0.87	0.53	0.22
Pathological assessment				
Laterality	Bilateral vs. unilateral	0.92	0.93	0.55
Pathology Stage	Stage III/IV vs. I/II	0.73	0.82	0.44
T stage	T3 vs. T1/2	0.36	0.57	0.36
Lymph node metastasis	N1 vs N0	0.21	**0.036**	**0.017**
Distant metastasis	M1 vs. M0	0.71	0.80	0.32
Focality	Multi vs. unifocal	**0.037**	0.91	0.77
Intervention				
Thyroidectomy	Total/subtotal vs. lobectomy	0.21	0.46	0.99
Neck dissection	Positive vs. negative	0.53	0.65	0.35
Radioactive iodine	Positive vs. negative	0.50	0.33	0.13

Data on the *p*-values are shown for each microRNA. Mann–Whitney U test was used. Significant *p*-values < 0.05 are shown in the table in bold.

**Table 4:** Predictive accuracy of microRNAs in forecasting probabilities of tumor progression.

Group	AUC	*p*-Value	Cutoff	Sensitivity	Specificity	+LR	−LR	+PV	−PV	Cost
miR-204										
Overall	0.856 (0.74–0.93)	**<0.001**	≤−1.15	71.4 (53.7–85.4)	90.9 (75.7–98.1)	7.9	0.30	89.3	75	0.191
RAI	0.918 (0.75–0.98)	**<0.001**	≤−1	85 (62.1–96.8)	92.9 (66.1–99.8)	11.9	0.20	94.4	81.2	0.118
Non-RAI	0.821 (0.71–0.90)	**<0.001**	≤−1.4	66.7 (47.2–82.7)	89.5 (75.2–97.1)	6.33	0.37	83.3	77.3	0.206
miR-221										
Overall	0.930 (0.82–0.97)	**<0.001**	>1.03	88.6 (73.3–96.8)	97 (84.2–99.9)	29.2	0.10	96.9	88.9	0.074
RAI	0.979 (0.87–1.00)	**<0.001**	>1.03	95 (75.1–99.9)	100 (76.8–100.0)	NA	0.10	100	93.3	0.029
Non-RAI	0.856 (0.75–0.93)	**<0.001**	>1.0	80 (61.4–92.3)	94.7 (82.3–99.4)	15.2	0.21	92.3	85.7	0.118
miR-222										
Overall	0.854 (0.74–0.92)	**<0.001**	>1.2	82.9 (66.4–93.4)	81.8 (64.5–93.0)	4.6	0.2 0	82.9	81.8	0.176
RAI	0.838 (0.66–0.94)	**<0.001**	>1.2	85 (62.1–96.8)	78.6 (49.2–95.3)	4.0	0.20	85	78.6	0.176
Non-RAI	0.860 (0.75–0.93)	**<0.001**	>1.25	73.3 (54.1–87.7)	94.7 (82.3–99.4)	13.9	0.28	91.7	81.8	0.147
ATA score										
Overall	0.613 (0.49–0.73)	0.06	>8	97 (85.1–99.9)	18.2 (7.0–35.5)	1.19	0.16	55.7	85.7	0.412
RAI	0.514 (0.34–0.68)	0.88	≤10	60 (36.1–80.9)	57.1 (28.9–82.3)	1.4	0.70	66.7	50.0	0.412
Non-RAI	0.677 (0.49–0.82)	**0.012**	>8	93.3 (68.1–99.8)	21 (6.1–45.6)	1.18	0.30	48.3	80.0	0.471

Receiver operating characteristic curve. AUC: area under the curve, CI: confidence interval of 1000 bootstrap iteration, LR: likelihood ratio, PV: predictive value. The area under the curve (AUC) for each microRNA is estimated. The larger the AUC, the better the discrimination power of the marker. Diagnostic accuracy measures were estimated at the best cutoff value. Significant *p*-values < 0.05 are shown in the table in bold.

**Table 5:** Cox proportionate hazard regression analysis.

Risk Factor	Univariate Cox Regression			Multivariate Cox Regression		
	HR	95%CI	*p*-Value	HR	95%CI	*p*-Value
miR-204-3p	0.58	0.44–0.76	**<0.001**	0.77	0.57–0.99	**0.003**
miR-221-5p	1.95	1.48–2.56	**<0.001**	1.68	1.20–2.35	**0.002**
miR-222-5p	1.96	1.32–2.91	**0.001**	1.59	1.14–2.21	**0.027**

Multivariate analysis adjusted by demographic features yielded similar results for the microRNA. HR: hazard ratio; 95% CI: 95% bootstrap confidence interval. Significant *p*-values < 0.05 are shown in the table in bold.

**Table 6:** Prognostic value of microRNA risk score in thyroid cancer patients.

MicroRNA Risk Score	Not Have Event		Have Event		*p*-Value	C-Statistic	Brier Score
	*N* (%)	Median Score (IQR)	*N* (%)	Median Score (IQR)			
Overall analysis							
Advanced T stage	48 (70.6%)	0.83 (0.36–2.14)	20 (29.4%)	0.84 (0.46–1.71)	0.67	0.532	0.205
Lymph node metastasis	36 (52.9%)	0.79 (0.40–1.72)	32 (47.1%)	0.93 (0.32–2.15)	0.78	0.519	0.247
Distant metastasis	54 (79.4%)	0.73 (0.35–1.82)	14 (20.6%)	1.38 (0.77–2.25)	0.12	0.632	0.158
Multifocality	29 (42.6%)	0.71 (0.37–1.83)	39 (57.4%)	0.90 (0.40–2.15)	0.34	0.566	0.241
Bilateral lesion	47 (69.1%)	0.82 (0.39–1.94)	21 (30.9%)	0.85 (0.28–1.77)	0.58	0.542	0.212
Recurrence	48 (70.6%)	0.60 (0.29–1.35)	20 (29.4%)	2.0 (1.23–2.44)	**<0.001**	**0.820**	0.156
Progression	33 (48.5%)	0.39 (0.24–0.71)	35 (51.5%)	1.87 (1.27–2.29)	**<0.001**	**0.943**	**0.083**
Mortality	63 (92.6%)	0.77 (0.35–1.96)	5 (7.4%)	1.47 (1.02–1.75)	0.30	0.644	**0.068**
Subgroup analysis							
Advanced T stage	26 (76.5%)	1.51 (0.37–2.37)	8 (23.5%)	0.89 (0.56–1.92)	0.41	0.601	0.177
Lymph node metastasis	16 (47.1%)	0.83 (0.56–2.22)	18 (52.9%)	1.83 (0.34–2.34)	0.59	0.555	0.244
Distant metastasis	24 (70.6%)	1.12 (0.38–2.24)	10 (29.4%)	1.61 (0.39–2.36)	0.75	0.537	0.207
Multifocality	14 (41.2%)	1.51 (0.37–2.02)	20 (58.8%)	0.96 (0.56–2.32)	0.74	0.535	0.241
Bilateral lesion	20 (58.8%)	1.78 (0.48–2.28)	14 (41.2%)	0.79 (0.26–2.26)	0.37	0.592	0.235
Recurrence	26 (76.5%)	0.77 (0.34–1.89)	8 (23.5%)	2.39 (2.22–2.75)	**0.001**	**0.870**	0.126
Progression	14 (41.2%)	0.37 (0.2–0.73)	20 (58.8%)	2.22 (1.76–2.49)	**<0.001**	**0.978**	**0.049**
Mortality	32 (94.1%)	1.01 (0.38–2.28)	2 (5.9%)	1.57 (1.27–1.87)	0.80	0.562	**0.055**

IQR: interquartile range. A C-statistic of 1.0 represents ideal discrimination, indicating the model is ideal for predicting with a greater probability a patient experiencing an event compared with a patient who does not. Alternatively, a C-statistic of 0.5 indicates that the model is no better at classifying outcomes than random chance. As a priori, we set a C-statistic >0.8 to indicate excellent discrimination, between 0.7 and 0.8 indicates reasonable or good discrimination, and between 0.5 and 0.7 indicates poor or weak discrimination. The Brier score ranges from zero to one and represents the square of the largest possible difference between a predicted probability and the actual outcome. Therefore, the lower the Brier score for a set of predictions, the better the predictions are calibrated. Significant *p*-values < 0.05, Brier score, and/or C-Statistic results are shown in the table in bold.

**Table 7:** Literature review of deregulated study microRNAs in cancer.

Cancer Type	Tumor Subtype or Cell Line	Design	Expression Status	Ref.
miR-204-5p				
Melanoma	Cutaneous malignant melanoma	cancer vs. normal	down	[48]
Melanoma	Malme-3M, SKMEL-28, and SKMEL-11	metastatic	down	[49]
Gastric cancer	Subtype1: Helicobacter pylori-positive cancer, Subtype2: Helicobacter pylori-negative cancer	subtype1 vs. subtype2	down	[50]
miR-221-3p				
Head and neck cancer	N/A	cancer vs. normal	up	[51]
Brain cancer	Schwannomas	cancer vs. normal	up	[52]
Hepatocellular carcinoma	PHHC-3	cancer vs. normal	up	[53]
Colon cancer	N/A	cancer vs. normal	up	[54]
Cholangiocarcinoma	N/A	cancer vs. normal	up	[55]
Lymphoma	Multiple myeloma (TC5)	subtype1 vs. subtype2	up	[56]
Lymphoma	Nodal marginal zone lymphoma/lymphoid hyperplasia	subtype1 vs. subtype2	up	[57]
miR-222-3p				
Cholangiocarcinoma	N/A	cancer vs. normal	up	[55]
Lymphoma	Multiple myeloma (TC4)	subtype1 vs. subtype2	up	[56]

Experiments were performed in qRT-PCR.

## Data Availability

Data are available from the corresponding author upon reasonable request and obtaining the approval of the “Office of Technology Transfer and Intellectual Property Development, Tulane University, USA”.

## Supplementary Materials

The following are available online at https://www.mdpi.com/article/10.3390/cancers13184649/s1, Table S1: Tested microRNAs in the study cohorts, Table S2: Enriched microRNAs in thyroid cancer KEGG pathway (hsa05216), Table S3: MiRNA-seq experiments of thyroid carcinoma with different comparisons, Table S4: Meta-profiling of thyroid cancer diagnostic and prognostic microRNAs, Table S5: Pathway enrichment analysis of 104 deregulated microRNAs in thyroid cancer, Table S6: Downregulated miR-204-5p in various cancer types, Table S7: Upregulated miR-221-3p in various cancer types, Table S8: Upregulated miR-222-3p in various cancer types, Table S9: Summary of molecular pathways regulating the study microRNAs in cancer.
