# Supplementary material for: MicroRNA-Based Risk Score for Predicting Tumor Progression Following Radioactive Iodine Ablation in Well-Differentiated Thyroid Cancer Patients: A Propensity-Score Matched Analysis
Source: Cancers (Basel). 2021 Sep 16;13(18):4649. doi: 10.3390/cancers13184649 (PMC8468667; doi:10.3390/cancers13184649)
Supplement: Supplementary file 1 [file cancers-13-04649-s001.zip › cancers-1256367-supplementary.pdf]

Supplementary Table S1. Tested microRNAs in the study cohorts

microRNA ID	Accession	TaqMan assay ID	Genomic location	Mature sequence
hsa-miR-204-5p	MIMAT0000265	000508	9q21.12	UUCCCUUUGUCAUCCUAUGCCU
hsa-miR-221-3p	MIMAT0000278	000524	Xp11.3	AGCUACAUUGUCUGCUGGGUUUC
hsa-miR-222-3p	MIMAT0000279	002276	Xp11.3	AGCUACAUCUGGCUACUGGGU

Data source: miRbase version 22.0 (http://www.mirbase.org/).

Supplementary Table S2. Enriched microRNAs in thyroid cancer KEGG pathway (hsa05216)

Rank	miRNA	p-value	#genes targeted
1	hsa-miR-34a-5p	1.87E-67	17
2	hsa-miR-27a-3p	1.27E-40	11
3	hsa-miR-181a-5p	1.27E-40	11
4	hsa-miR-92a-3p	1.27E-40	11
5	hsa-miR-16-5p	1.27E-40	11
6	hsa-miR-522-5p	1.89E-36	10
7	hsa-miR-183-5p	1.89E-36	10
8	hsa-miR-93-5p	1.89E-36	10
9	hsa-miR-17-5p	2.30E-32	9
10	hsa-let-7a-5p	2.30E-32	9
11	hsa-miR-106b-5p	2.29E-28	8
12	hsa-let-7g-5p	2.29E-28	8
13	hsa-miR-17-3p	2.29E-28	8
14	hsa-miR-30a-5p	2.29E-28	8
15	hsa-miR-15a-5p	2.29E-28	8
16	hsa-miR-21-5p	2.29E-28	8
17	hsa-miR-20a-5p	2.29E-28	8
18	hsa-let-7b-5p	2.29E-28	8
19	hsa-miR-106a-5p	2.29E-28	8
20	hsa-let-7i-5p	1.85E-24	7
21	hsa-miR-29b-3p	1.85E-24	7
22	hsa-miR-128	1.85E-24	7
23	hsa-miR-30c-5p	1.85E-24	7
24	hsa-miR-30e-5p	1.85E-24	7
25	hsa-miR-218-5p	1.85E-24	7
26	hsa-miR-182-5p	1.85E-24	7
27	hsa-let-7f-5p	1.85E-24	7
28	hsa-miR-130a-3p	1.85E-24	7
29	hsa-miR-424-5p	1.85E-24	7
30	hsa-miR-27b-3p	1.20E-20	6
31	hsa-miR-193b-3p	1.20E-20	6
32	hsa-let-7d-5p	1.20E-20	6
33	hsa-miR-196a-5p	1.20E-20	6
34	hsa-miR-145-5p	1.20E-20	6
35	hsa-miR-98	1.20E-20	6
36	hsa-miR-23a-3p	1.20E-20	6
37	hsa-miR-26b-5p	1.20E-20	6
38	hsa-miR-30d-5p	1.20E-20	6
39	hsa-miR-25-3p	1.20E-20	6
40	hsa-miR-148a-3p	1.20E-20	6
41	hsa-miR-148b-3p	1.20E-20	6

42	hsa-miR-423-3p	1.20E-20	6
43	hsa-miR-19a-3p	1.20E-20	6
44	hsa-miR-15b-5p	1.20E-20	6
45	hsa-miR-107	1.20E-20	6
46	hsa-miR-320b	6.09E-17	5
47	hsa-miR-320a	6.09E-17	5
48	hsa-let-7e-5p	6.09E-17	5
49	hsa-miR-374a-5p	6.09E-17	5
50	hsa-miR-340-5p	6.09E-17	5
51	hsa-miR-195-5p	6.09E-17	5
52	hsa-miR-766-3p	6.09E-17	5
53	hsa-miR-423-5p	6.09E-17	5
54	hsa-miR-221-5p	6.09E-17	5
55	hsa-miR-200c-3p	6.09E-17	5
56	hsa-miR-7-5p	6.09E-17	5
57	hsa-miR-23b-3p	6.09E-17	5
58	hsa-miR-205-5p	6.09E-17	5
59	hsa-miR-203	6.09E-17	5
60	hsa-miR-1291	6.09E-17	5
61	hsa-miR-19b-3p	6.09E-17	5
62	hsa-miR-103a-3p	6.09E-17	5
63	hsa-miR-143-3p	6.09E-17	5
64	hsa-miR-22-3p	6.09E-17	5
65	hsa-miR-26a-5p	6.09E-17	5
66	hsa-let-7c	6.09E-17	5
67	hsa-miR-9-5p	6.09E-17	5
68	hsa-miR-32-5p	2.38E-13	4
69	hsa-miR-221-3p	2.38E-13	4
70	hsa-let-7b-3p	2.38E-13	4
71	hsa-miR-196b-5p	2.38E-13	4
72	hsa-miR-590-3p	2.38E-13	4
73	hsa-miR-34b-3p	2.38E-13	4
74	hsa-miR-126-3p	2.38E-13	4
75	hsa-miR-125a-5p	2.38E-13	4
76	hsa-miR-877-3p	2.38E-13	4
77	hsa-miR-24-3p	2.38E-13	4
78	hsa-miR-96-5p	2.38E-13	4
79	hsa-miR-214-3p	2.38E-13	4
80	ebv-miR-BART17-5p	2.38E-13	4
81	hsa-miR-497-5p	2.38E-13	4
82	hsa-miR-192-5p	2.38E-13	4
83	hsa-miR-126-5p	2.38E-13	4
84	hsa-miR-200b-3p	2.38E-13	4

85	hsa-miR-27a-5p	2.38E-13	4
86	hsa-miR-199a-3p	2.38E-13	4
87	hsa-miR-92b-3p	2.38E-13	4
88	hsa-miR-513a-5p	2.38E-13	4
89	hsa-miR-29a-3p	2.38E-13	4
90	hsa-miR-200a-3p	2.38E-13	4
91	hsa-miR-138-5p	2.38E-13	4
92	hsa-miR-21-3p	2.38E-13	4
93	hsa-miR-301a-3p	2.38E-13	4
94	hsa-miR-20b-5p	2.38E-13	4
95	hsa-miR-16-2-3p	6.87E-10	3
96	hsa-miR-320d	6.87E-10	3
97	hsa-miR-320c	6.87E-10	3
98	hsa-miR-148a-5p	6.87E-10	3
99	hsa-miR-122-5p	6.87E-10	3
100	hsa-miR-20a-3p	6.87E-10	3
101	hsa-miR-29c-3p	6.87E-10	3
102	hsa-miR-224-5p	6.87E-10	3
103	hsa-miR-324-5p	6.87E-10	3
104	hsa-miR-744-5p	6.87E-10	3
105	hsa-miR-181c-5p	6.87E-10	3
106	ebv-miR-BART22	6.87E-10	3
107	hsa-miR-151a-5p	6.87E-10	3
108	hsa-miR-125a-3p	6.87E-10	3
109	hsa-miR-335-5p	6.87E-10	3
110	hsa-miR-374b-5p	6.87E-10	3
111	hsa-miR-93-3p	6.87E-10	3
112	hsa-miR-22-5p	6.87E-10	3
113	hsa-miR-142-5p	6.87E-10	3
114	hsa-miR-30b-5p	6.87E-10	3
115	hsa-miR-361-5p	6.87E-10	3
116	hsa-miR-125b-5p	6.87E-10	3
117	hsa-miR-615-3p	6.87E-10	3
118	hsa-miR-124-3p	6.87E-10	3
119	hsa-miR-105-5p	6.87E-10	3
120	hsa-miR-429	6.87E-10	3
121	hsa-miR-183-3p	6.87E-10	3
122	hsa-miR-141-3p	6.87E-10	3
123	hsa-miR-454-5p	6.87E-10	3
124	hsa-miR-375	6.87E-10	3
125	hsa-miR-139-5p	6.87E-10	3
126	hsa-miR-186-5p	6.87E-10	3
127	hsa-miR-132-3p	6.87E-10	3

128	hsa-miR-151a-3p	6.87E-10	3
129	hsa-miR-222-3p	6.87E-10	3
130	hsa-miR-142-3p	6.87E-10	3
131	hsa-miR-378a-3p	6.87E-10	3
132	hsa-miR-197-3p	6.87E-10	3
133	hsa-miR-330-5p	6.87E-10	3
134	hsa-miR-217	6.87E-10	3
135	hsa-miR-377-3p	6.87E-10	3
136	hsa-miR-181b-5p	6.87E-10	3
137	hsa-miR-548d-5p	1.38E-06	2
138	hsa-miR-548am-5p	1.38E-06	2
139	hsa-miR-548o-5p	1.38E-06	2
140	hsa-miR-33a-5p	1.38E-06	2
141	hsa-miR-34c-5p	1.38E-06	2
142	hsa-miR-4745-5p	1.38E-06	2
143	hsa-miR-23b-5p	1.38E-06	2
144	hsa-miR-101-3p	1.38E-06	2
145	hsa-miR-205-3p	1.38E-06	2
146	hsa-miR-30e-3p	1.38E-06	2
147	ebv-miR-BART21-3p	1.38E-06	2
148	hsa-miR-2682-5p	1.38E-06	2
149	hsa-miR-5010-3p	1.38E-06	2
150	hsa-miR-202-3p	1.38E-06	2
151	hsa-miR-484	1.38E-06	2
152	hsa-miR-1224-5p	1.38E-06	2
153	hsa-miR-148b-5p	1.38E-06	2
154	hsa-miR-7-1-3p	1.38E-06	2
155	hsa-miR-193a-3p	1.38E-06	2
156	hsa-miR-502-3p	1.38E-06	2
157	hsa-miR-130b-5p	1.38E-06	2
158	hsa-miR-548ak	1.38E-06	2
159	hsa-miR-532-5p	1.38E-06	2
160	hsa-miR-191-3p	1.38E-06	2
161	hsa-miR-301b	1.38E-06	2
162	hsa-miR-606	1.38E-06	2
163	hsa-miR-608	1.38E-06	2
164	hsa-miR-133a	1.38E-06	2
165	hsa-miR-363-3p	1.38E-06	2
166	hsa-miR-548d-3p	1.38E-06	2
167	hsa-miR-216a	1.38E-06	2
168	hsa-miR-1	1.38E-06	2
169	hsa-miR-449c-5p	1.38E-06	2
170	hsa-miR-876-3p	1.38E-06	2

171	hsa-miR-378a-5p	1.38E-06	2
172	hsa-miR-425-5p	1.38E-06	2
173	hsa-miR-3934	1.38E-06	2
174	hsa-miR-186-3p	1.38E-06	2
175	hsa-miR-641	1.38E-06	2
176	kshv-miR-K12-5*	1.38E-06	2
177	hsa-miR-29a-5p	1.38E-06	2
178	hsa-miR-19b-1-5p	1.38E-06	2
179	hsa-miR-494	1.38E-06	2
180	hsa-miR-625-5p	1.38E-06	2
181	hsa-miR-99a-5p	1.38E-06	2
182	hsa-miR-338-5p	1.38E-06	2
183	hsa-miR-769-5p	1.38E-06	2
184	hsa-miR-506-3p	1.38E-06	2
185	hsa-miR-330-3p	1.38E-06	2
186	hsa-miR-10b-5p	1.38E-06	2
187	hsa-miR-940	1.38E-06	2
188	hsa-miR-575	1.38E-06	2
189	hsa-miR-138-2-3p	1.38E-06	2
190	hsa-miR-449a	1.38E-06	2
191	hsa-miR-1224-3p	1.38E-06	2
192	hsa-miR-103a-2-5p	1.38E-06	2
193	hsa-miR-548c-5p	1.38E-06	2
194	hsa-miR-130b-3p	1.38E-06	2
195	hsa-miR-30a-3p	1.38E-06	2
196	hsa-miR-361-3p	1.38E-06	2
197	hsa-miR-181a-2-3p	1.38E-06	2
198	hsa-miR-92a-1-5p	1.38E-06	2
199	hsa-miR-582-5p	1.38E-06	2
200	hsa-miR-877-5p	1.38E-06	2
201	hsa-miR-181d	1.38E-06	2
202	hsa-miR-622	1.38E-06	2
203	hsa-miR-324-3p	1.38E-06	2
204	kshv-miR-K12-10b	1.38E-06	2
205	hsa-miR-551b-5p	1.38E-06	2
206	hsa-miR-28-5p	1.38E-06	2
207	hsa-miR-671-5p	1.38E-06	2
208	hsa-miR-548l	1.38E-06	2
209	hsa-miR-548k	1.38E-06	2
210	hsa-miR-548j	1.38E-06	2
211	hsa-miR-155-5p	1.38E-06	2
212	hsa-miR-548z	1.38E-06	2
213	hsa-miR-548w	1.38E-06	2

214	hsa-miR-191-5p	1.38E-06	2
215	hsa-miR-593-5p	1.38E-06	2
216	hsa-miR-665	1.38E-06	2
217	ebv-miR-BART6-5p	1.38E-06	2
218	hsa-miR-29b-2-5p	1.38E-06	2
219	hsa-miR-135a-5p	1.38E-06	2
220	hsa-miR-451a	1.38E-06	2
221	hsa-miR-378c	1.72E-03	1
222	hsa-miR-3065-3p	1.72E-03	1
223	hsa-miR-576-3p	1.72E-03	1
224	hsa-miR-15a-3p	1.72E-03	1
225	hsa-miR-5682	1.72E-03	1
226	ebv-miR-BHRF1-2*	1.72E-03	1
227	hsa-miR-373-3p	1.72E-03	1
228	hsa-miR-342-3p	1.72E-03	1
229	hsa-miR-486-5p	1.72E-03	1
230	hsa-miR-378i	1.72E-03	1
231	hsa-miR-532-3p	1.72E-03	1
232	hsa-miR-526b-3p	1.72E-03	1
233	hsa-miR-29b-1-5p	1.72E-03	1
234	hsa-miR-18a-3p	1.72E-03	1
235	hsa-miR-302c-3p	1.72E-03	1
236	hsa-miR-3691-5p	1.72E-03	1
237	hsa-miR-450b-5p	1.72E-03	1
238	hsa-miR-382-5p	1.72E-03	1
239	hsa-miR-542-5p	1.72E-03	1
240	hsa-miR-4482-5p	1.72E-03	1
241	hsa-miR-151b	1.72E-03	1
242	hsa-miR-4491	1.72E-03	1
243	hsa-miR-127-5p	1.72E-03	1
244	hsa-miR-4792	1.72E-03	1
245	hsa-miR-4708-3p	1.72E-03	1
246	hsa-miR-527	1.72E-03	1
247	hsa-miR-521	1.72E-03	1
248	hsa-miR-766-5p	1.72E-03	1
249	hsa-miR-888-3p	1.72E-03	1
250	hsa-miR-1914-3p	1.72E-03	1
251	hsa-miR-124-5p	1.72E-03	1
252	hsa-miR-631	1.72E-03	1
253	hsa-miR-638	1.72E-03	1
254	hsa-miR-4420	1.72E-03	1
255	hsa-miR-4421	1.72E-03	1
256	hsa-miR-4425	1.72E-03	1

257	hsa-miR-625-3p	1.72E-03	1
258	hsa-miR-18b-5p	1.72E-03	1
259	hsa-miR-146a-5p	1.72E-03	1
260	hsa-miR-550b-2-5p	1.72E-03	1
261	hsa-miR-31-5p	1.72E-03	1
262	hsa-miR-455-5p	1.72E-03	1
263	hsa-miR-493-3p	1.72E-03	1
264	hsa-miR-4742-3p	1.72E-03	1
265	hsa-miR-624-3p	1.72E-03	1
266	hsa-miR-501-3p	1.72E-03	1
267	hsa-miR-34a-3p	1.72E-03	1
268	ebv-miR-BART14	1.72E-03	1
269	ebv-miR-BART10	1.72E-03	1
270	kshv-miR-K12-4-3p	1.72E-03	1
271	hsa-miR-574-3p	1.72E-03	1
272	hsa-miR-4693-5p	1.72E-03	1
273	hsa-miR-769-3p	1.72E-03	1
274	hsa-miR-411-5p	1.72E-03	1
275	hsa-miR-518a-5p	1.72E-03	1
276	hsa-miR-485-5p	1.72E-03	1
277	ebv-miR-BART8*	1.72E-03	1
278	hsa-miR-708-5p	1.72E-03	1
279	hsa-miR-302d-3p	1.72E-03	1
280	hsa-miR-150-3p	1.72E-03	1
281	ebv-miR-BART11-5p	1.72E-03	1
282	hsa-miR-222-5p	1.72E-03	1
283	kshv-miR-K12-3*	1.72E-03	1
284	hsa-miR-628-5p	1.72E-03	1
285	hsa-miR-664-3p	1.72E-03	1
286	hsa-let-7f-1-3p	1.72E-03	1
287	hsa-miR-4473	1.72E-03	1
288	hsa-miR-4254	1.72E-03	1
289	hsa-miR-30d-3p	1.72E-03	1
290	hsa-miR-16-1-3p	1.72E-03	1
291	hsa-let-7a-3p	1.72E-03	1
292	hsa-miR-3679-5p	1.72E-03	1
293	hsa-miR-412	1.72E-03	1
294	hsa-miR-582-3p	1.72E-03	1
295	hsa-miR-616-5p	1.72E-03	1
296	hsa-miR-516a-5p	1.72E-03	1
297	hsa-miR-552	1.72E-03	1
298	ebv-miR-BART17-3p	1.72E-03	1
299	hsa-miR-9-3p	1.72E-03	1

300	hsa-miR-204-5p	1.72E-03	1
301	ebv-miR-BART20-3p	1.72E-03	1
302	hsa-miR-338-3p	1.72E-03	1
303	hsa-miR-4677-3p	1.72E-03	1
304	hsa-miR-1303	1.72E-03	1
305	hsa-miR-4690-5p	1.72E-03	1
306	hsa-miR-365b-5p	1.72E-03	1
307	hsa-miR-379-5p	1.72E-03	1
308	hsa-miR-129-5p	1.72E-03	1
309	hsa-miR-340-3p	1.72E-03	1
310	hsa-miR-483-5p	1.72E-03	1
311	hsa-miR-1246	1.72E-03	1
312	hsa-miR-1248	1.72E-03	1
313	hsa-miR-140-5p	1.72E-03	1
314	hsa-miR-5100	1.72E-03	1
315	kshv-miR-K12-6-3p	1.72E-03	1
316	hsa-miR-18a-5p	1.72E-03	1
317	hsa-miR-130a-5p	1.72E-03	1
318	hsa-miR-181c-3p	1.72E-03	1
319	hsa-miR-1234	1.72E-03	1
320	hsa-let-7f-2-3p	1.72E-03	1
321	hsa-miR-607	1.72E-03	1
322	hsa-miR-603	1.72E-03	1
323	hsa-miR-346	1.72E-03	1
324	hsa-miR-136-3p	1.72E-03	1
325	hsa-miR-1226-3p	1.72E-03	1
326	hsa-miR-181b-3p	1.72E-03	1
327	hsa-miR-323a-5p	1.72E-03	1
328	hsa-miR-511	1.72E-03	1
329	hsa-miR-3159	1.72E-03	1
330	kshv-miR-K12-10a*	1.72E-03	1
331	hsa-miR-548au-3p	1.72E-03	1
332	hsa-miR-875-3p	1.72E-03	1
333	hsa-miR-33a-3p	1.72E-03	1
334	hsa-miR-211-5p	1.72E-03	1
335	hsa-miR-199a-5p	1.72E-03	1
336	hsa-miR-486-3p	1.72E-03	1
337	hsa-miR-449b-5p	1.72E-03	1
338	hsa-miR-636	1.72E-03	1
339	hsa-miR-106b-3p	1.72E-03	1
340	kshv-miR-K12-2*	1.72E-03	1
341	hsa-miR-518c-5p	1.72E-03	1
342	hsa-miR-101-5p	1.72E-03	1

343	hsa-miR-378f	1.72E-03	1
344	hsa-miR-378g	1.72E-03	1
345	hsa-miR-378d	1.72E-03	1
346	kshv-miR-K12-1	1.72E-03	1
347	ebv-miR-BART2-3p	1.72E-03	1
348	hsa-miR-1207-5p	1.72E-03	1
349	hsa-miR-431-3p	1.72E-03	1
350	hsa-miR-643	1.72E-03	1
351	hsa-miR-647	1.72E-03	1
352	hsa-miR-331-5p	1.72E-03	1
353	hsa-miR-760	1.72E-03	1
354	hsa-miR-765	1.72E-03	1
355	hsa-miR-520b	1.72E-03	1
356	hsa-miR-767-5p	1.72E-03	1
357	hsa-miR-498	1.72E-03	1
358	hsa-miR-1301	1.72E-03	1
359	hsa-miR-495	1.72E-03	1
360	hsa-miR-181a-3p	1.72E-03	1
361	ebv-miR-BHRF1-1	1.72E-03	1
362	hsa-miR-545-3p	1.72E-03	1
363	hsa-miR-509-3-5p	1.72E-03	1
364	hsa-miR-1285-3p	1.72E-03	1
365	hsa-miR-137	1.72E-03	1
366	hsa-miR-421	1.72E-03	1
367	hsa-miR-196a-3p	1.72E-03	1
368	hsa-miR-944	1.72E-03	1
369	hsa-miR-520d-5p	1.72E-03	1
370	hsa-miR-941	1.72E-03	1
371	hsa-miR-629-3p	1.72E-03	1
372	hsa-miR-518a-3p	1.72E-03	1
373	hsa-miR-34b-5p	1.72E-03	1
374	hsa-miR-147a	1.72E-03	1
375	hsa-miR-141-5p	1.72E-03	1
376	hsa-miR-708-3p	1.72E-03	1
377	hsa-miR-520d-3p	1.72E-03	1
378	hsa-miR-365a-3p	1.72E-03	1
379	hsa-miR-654-3p	1.72E-03	1
380	hsa-miR-193b-5p	1.72E-03	1
381	kshv-miR-K12-11	1.72E-03	1
382	hsa-miR-487a	1.72E-03	1
383	hsa-miR-10a-5p	1.72E-03	1
384	hsa-miR-7-2-3p	1.72E-03	1
385	hsa-miR-595	1.72E-03	1

386	hsa-miR-612	1.72E-03	1
387	kshv-miR-K12-6-5p	1.72E-03	1
388	hsa-miR-28-3p	1.72E-03	1
389	hsa-miR-579	1.72E-03	1
390	hsa-miR-539-5p	1.72E-03	1
391	hsa-miR-373-5p	1.72E-03	1
392	hsa-miR-548h-3p	1.72E-03	1
393	ebv-miR-BART4	1.72E-03	1
394	hsa-miR-3615	1.72E-03	1
395	hsa-miR-3617	1.72E-03	1
396	hsa-miR-2278	1.72E-03	1
397	ebv-miR-BART20-5p	1.72E-03	1
398	hsa-miR-634	1.72E-03	1
399	hsa-miR-1290	1.72E-03	1
400	hsa-miR-26a-2-3p	1.72E-03	1
401	hsa-miR-503	1.72E-03	1
402	hsa-miR-1294	1.72E-03	1
403	hsa-miR-452-5p	1.72E-03	1
404	hsa-miR-509-5p	1.72E-03	1
405	hsa-miR-32-3p	1.72E-03	1
406	hsa-miR-450a-3p	1.72E-03	1
407	hsa-miR-580	1.72E-03	1
408	hsa-miR-3681-5p	1.72E-03	1
409	hsa-miR-513b	1.72E-03	1
410	hsa-miR-212-3p	1.72E-03	1
411	hsa-miR-1910	1.72E-03	1
412	hsa-miR-26a-1-3p	1.72E-03	1
413	hsa-miR-2355-3p	1.72E-03	1
414	hsa-miR-2110	1.72E-03	1
415	hsa-miR-150-5p	1.72E-03	1
416	hsa-miR-675-5p	1.72E-03	1
417	hsa-miR-874	1.72E-03	1
418	hsa-miR-3607-3p	1.72E-03	1
419	hsa-miR-626	1.72E-03	1
420	hsa-miR-628-3p	1.72E-03	1
421	hsa-miR-185-3p	1.72E-03	1
422	hsa-miR-548au-5p	1.72E-03	1
423	hsa-miR-30b-3p	1.72E-03	1
424	hsa-miR-140-3p	1.72E-03	1
425	hsa-miR-4484	1.72E-03	1
426	hsa-miR-657	1.72E-03	1
427	hsa-miR-655	1.72E-03	1
428	hsa-miR-876-5p	1.72E-03	1

429	hsa-miR-651	1.72E-03	1
430	hsa-miR-802	1.72E-03	1
431	hsa-miR-27b-5p	1.72E-03	1
432	hsa-miR-125b-2-3p	1.72E-03	1
433	hsa-miR-374c-5p	1.72E-03	1
434	hsa-miR-455-3p	1.72E-03	1
435	hsa-miR-1260a	1.72E-03	1
436	hsa-miR-1260b	1.72E-03	1
437	hsa-miR-153	1.72E-03	1
438	hsa-miR-296-5p	1.72E-03	1
439	hsa-miR-328	1.72E-03	1
440	hsa-miR-411-3p	1.72E-03	1
441	hsa-miR-146b-5p	1.72E-03	1
442	hsa-miR-589-5p	1.72E-03	1
443	hsa-miR-422a	1.72E-03	1
444	hsa-miR-323a-3p	1.72E-03	1
445	hsa-miR-502-5p	1.72E-03	1
446	kshv-miR-K12-9*	1.72E-03	1
447	hsa-miR-302a-3p	1.72E-03	1
448	hsa-miR-1304-5p	1.72E-03	1
449	hsa-miR-454-3p	1.72E-03	1
450	hsa-miR-136-5p	1.72E-03	1
451	hsa-miR-659-3p	1.72E-03	1
452	hsa-miR-302b-3p	1.72E-03	1
453	hsa-miR-548b-3p	1.72E-03	1
454	hsa-miR-187-5p	1.72E-03	1
455	hsa-miR-3157-5p	1.72E-03	1
456	hsa-miR-920	1.72E-03	1
457	hsa-miR-922	1.72E-03	1
458	hsa-miR-219-1-3p	1.72E-03	1
459	ebv-miR-BART5	1.72E-03	1
460	hsa-miR-374b-3p	1.72E-03	1
461	hsa-miR-182-3p	1.72E-03	1
462	hsa-miR-331-3p	1.72E-03	1
463	hsa-miR-4510	1.72E-03	1
464	hsa-miR-4511	1.72E-03	1
465	hsa-miR-135b-5p	1.72E-03	1
466	hsa-miR-3609	1.72E-03	1
467	hsa-miR-3174	1.72E-03	1
468	hsa-miR-1287	1.72E-03	1
469	hsa-miR-1281	1.72E-03	1

Data source: DIANA-miRPath v.3.0 (http://www.microrna.gr/miRPathv2).

Supplementary Table S3. MiRNA-seq experiments of thyroid carcinoma with different comparisons

Experiment ID	Sample Case	Sample Control	Up miRNA	Down miRNA	Sample size	Cases	Controls
EXP00396	Cancer	Normal tissue	193	174	558	501	57
EXP00397	Metastasis	Non-metastasis	2	0	509	8	501
EXP00398	Thyroid carcinoma 2	Thyroid carcinoma 1	72	40	339	52	287
EXP00399	Thyroid carcinoma 3	Thyroid carcinoma 1	18	13	400	113	287
EXP00400	Thyroid carcinoma 4	Thyroid carcinoma 1	33	14	342	55	287
EXP00401	Thyroid carcinoma 3	Thyroid carcinoma 2	79	109	165	113	52
EXP00402	Thyroid carcinoma 4	Thyroid carcinoma 2	78	107	107	55	52

Data source: dbDEMC (https://www.biosino.org/dbDEMC/index). A total of 621 microRNAs were analyzed.

Supplementary Table S4. Meta-profiling of thyroid cancer diagnostic and prognostic microRNAs

miRNA ID	GEO ID	Comparison	logFC	Expression Status	Experiment ID
hsa-miR-410	GSE40807	cancer *vs.* normal	6.43	UP	EXP00274
hsa-miR-129-5p	GSE40807	cancer *vs.* normal	5.59	UP	EXP00274
hsa-miR-409-3p	GSE40807	cancer *vs.* normal	5.37	UP	EXP00274
hsa-miR-592	GSE40807	cancer *vs.* normal	5.31	UP	EXP00274
hsa-miR-153	GSE40807	cancer *vs.* normal	5.31	UP	EXP00274
hsa-miR-375	GSE40807	cancer *vs.* normal	5.19	UP	EXP00274
hsa-miR-124	GSE40807	cancer *vs.* normal	5.01	UP	EXP00274
hsa-miR-146b-3p	TCGA_THCA	cancer *vs.* normal	4.93	UP	EXP00396
hsa-miR-146b-5p	TCGA_THCA	cancer *vs.* normal	4.8	UP	EXP00396
hsa-miR-323-3p	GSE40807	cancer *vs.* normal	4.7	UP	EXP00274
hsa-miR-136*	GSE40807	cancer *vs.* normal	4.58	UP	EXP00274
hsa-miR-154	GSE40807	cancer *vs.* normal	4.39	UP	EXP00274
hsa-miR-382	GSE40807	cancer *vs.* normal	4.29	UP	EXP00274
hsa-miR-129-3p	GSE40807	cancer *vs.* normal	4.27	UP	EXP00274
hsa-miR-539	GSE40807	cancer *vs.* normal	4.26	UP	EXP00274
hsa-miR-495	GSE40807	cancer *vs.* normal	4.25	UP	EXP00274
hsa-miR-129*	GSE40807	cancer *vs.* normal	4.2	UP	EXP00274
hsa-miR-379	GSE40807	cancer *vs.* normal	4.11	UP	EXP00274
hsa-miR-376a*	GSE40807	cancer *vs.* normal	4.07	UP	EXP00274
hsa-miR-432	GSE40807	cancer *vs.* normal	3.55	UP	EXP00274
hsa-miR-381	GSE40807	cancer *vs.* normal	3.53	UP	EXP00274
hsa-miR-433	GSE40807	cancer *vs.* normal	3.49	UP	EXP00274
hsa-miR-127-3p	GSE40807	cancer *vs.* normal	3.39	UP	EXP00274
hsa-miR-137	GSE40807	cancer *vs.* normal	3.38	UP	EXP00274
hsa-miR-136	GSE40807	cancer *vs.* normal	3.31	UP	EXP00274
hsa-miR-654-3p	GSE40807	cancer *vs.* normal	3.26	UP	EXP00274
hsa-miR-487b	GSE40807	cancer *vs.* normal	3.21	UP	EXP00274
hsa-miR-330-3p	GSE40807	cancer *vs.* normal	3.15	UP	EXP00274

hsa-miR-337-5p	GSE40807	cancer *vs.* normal	3.11	UP	EXP00274
hsa-miR-551b-3p	TCGA_THCA	cancer *vs.* normal	3.07	UP	EXP00396
hsa-miR-376c	GSE40807	cancer *vs.* normal	3.02	UP	EXP00274
hsa-miR-9	GSE40807	cancer *vs.* normal	2.97	UP	EXP00274
hsa-miR-221-3p	TCGA_THCA	cancer *vs.* normal	2.95	UP	EXP00396
hsa-miR-9*	GSE40807	cancer *vs.* normal	2.94	UP	EXP00274
hsa-miR-377	GSE40807	cancer *vs.* normal	2.87	UP	EXP00274
hsa-miR-335*	GSE40807	cancer *vs.* normal	2.81	UP	EXP00274
hsa-miR-183	GSE40807	cancer *vs.* normal	2.79	UP	EXP00274
hsa-miR-146b-3p	TCGA_THCA	high grade *vs.* low grade	2.79	UP	EXP00402
hsa-miR-146b-5p	TCGA_THCA	high grade *vs.* low grade	2.79	UP	EXP00402
hsa-miR-409-5p	GSE40807	cancer *vs.* normal	2.75	UP	EXP00274
hsa-miR-335	GSE40807	cancer *vs.* normal	2.73	UP	EXP00274
hsa-miR-758	GSE40807	cancer *vs.* normal	2.72	UP	EXP00274
hsa-miR-132*	GSE40807	cancer *vs.* normal	2.69	UP	EXP00274
hsa-miR-222-3p	TCGA_THCA	cancer *vs.* normal	2.69	UP	EXP00396
hsa-miR-369-5p	GSE40807	cancer *vs.* normal	2.58	UP	EXP00274
hsa-miR-182	GSE40807	cancer *vs.* normal	2.57	UP	EXP00274
hsa-miR-431	GSE40807	cancer *vs.* normal	2.54	UP	EXP00274
hsa-miR-598	GSE40807	cancer *vs.* normal	2.54	UP	EXP00274
hsa-miR-7	GSE40807	cancer *vs.* normal	2.53	UP	EXP00274
hsa-miR-200a*	GSE40807	cancer *vs.* normal	2.48	UP	EXP00274
hsa-miR-429	GSE40807	cancer *vs.* normal	2.47	UP	EXP00274
hsa-miR-376a	GSE40807	cancer *vs.* normal	2.45	UP	EXP00274
hsa-miR-375	TCGA_THCA	cancer *vs.* normal	2.37	UP	EXP00396
hsa-miR-96	GSE40807	cancer *vs.* normal	2.34	UP	EXP00274
hsa-miR-889	GSE40807	cancer *vs.* normal	2.32	UP	EXP00274
hsa-miR-375	TCGA_THCA	high grade *vs.* low grade	2.32	UP	EXP00402
hsa-miR-543	GSE40807	cancer *vs.* normal	2.31	UP	EXP00274
hsa-miR-221	GSE40807	cancer *vs.* normal	2.3	UP	EXP00274
hsa-miR-199b-5p	TCGA_THCA	high grade *vs.* low grade	2.28	UP	EXP00401
hsa-miR-199b-5p	TCGA_THCA	high grade *vs.* low grade	2.25	UP	EXP00402
hsa-miR-34a-5p	TCGA_THCA	cancer *vs.* normal	2.23	UP	EXP00396
hsa-miR-200b*	GSE40807	cancer *vs.* normal	2.21	UP	EXP00274
hsa-miR-10a	GSE40807	cancer *vs.* normal	2.17	UP	EXP00274
hsa-miR-326	GSE40807	cancer *vs.* normal	2.14	UP	EXP00274
hsa-miR-376b	GSE40807	cancer *vs.* normal	2.11	UP	EXP00274
hsa-miR-4709-3p	TCGA_THCA	cancer *vs.* normal	2.1	UP	EXP00396
hsa-miR-10a*	GSE40807	cancer *vs.* normal	2.08	UP	EXP00274
hsa-miR-411	GSE40807	cancer *vs.* normal	2.08	UP	EXP00274
hsa-miR-7-1*	GSE40807	cancer *vs.* normal	2.07	UP	EXP00274
hsa-miR-485-5p	GSE40807	cancer *vs.* normal	2.05	UP	EXP00274
hsa-miR-485-3p	GSE40807	cancer *vs.* normal	2.05	UP	EXP00274
hsa-miR-146b-5p	TCGA_THCA	high grade *vs.* low grade	2.04	UP	EXP00401
hsa-miR-132	GSE40807	cancer *vs.* normal	2.02	UP	EXP00274
hsa-miR-222	GSE40807	cancer *vs.* normal	1.97	UP	EXP00274
hsa-miR-146b-3p	TCGA_THCA	high grade *vs.* low grade	1.94	UP	EXP00401
hsa-miR-329	GSE40807	cancer *vs.* normal	1.89	UP	EXP00274
hsa-miR-375	TCGA_THCA	high grade *vs.* low grade	1.87	UP	EXP00401
hsa-miR-431*	GSE40807	cancer *vs.* normal	1.85	UP	EXP00274
hsa-miR-299-5p	GSE40807	cancer *vs.* normal	1.83	UP	EXP00274

hsa-miR-487a	GSE40807	cancer *vs.* normal	1.82	UP	EXP00274
hsa-miR-324-5p	GSE40807	cancer *vs.* normal	1.79	UP	EXP00274
hsa-miR-5683	TCGA_THCA	metastasis	1.75	UP	EXP00397
hsa-miR-31-3p	TCGA_THCA	cancer *vs.* normal	1.72	UP	EXP00396
hsa-miR-181c	GSE40807	cancer *vs.* normal	1.66	UP	EXP00274
hsa-miR-31-5p	TCGA_THCA	cancer *vs.* normal	1.63	UP	EXP00396
hsa-miR-154*	GSE40807	cancer *vs.* normal	1.62	UP	EXP00274
hsa-miR-196a-5p	TCGA_THCA	metastasis	1.56	UP	EXP00397
hsa-miR-200a	GSE40807	cancer *vs.* normal	1.53	UP	EXP00274
hsa-miR-301b	GSE40807	cancer *vs.* normal	1.52	UP	EXP00274
hsa-miR-29b-2*	GSE40807	cancer *vs.* normal	1.47	UP	EXP00274
hsa-miR-221-5p	TCGA_THCA	cancer *vs.* normal	1.45	UP	EXP00396
hsa-miR-301a	GSE40807	cancer *vs.* normal	1.42	UP	EXP00274
hsa-miR-181a-2-3p	TCGA_THCA	cancer *vs.* normal	1.42	UP	EXP00396
hsa-miR-127-5p	TCGA_THCA	high grade *vs.* low grade	1.42	UP	EXP00402
hsa-miR-181b-5p	TCGA_THCA	cancer *vs.* normal	1.41	UP	EXP00396
hsa-miR-21-5p	TCGA_THCA	cancer *vs.* normal	1.39	UP	EXP00396
hsa-miR-205-5p	TCGA_THCA	high grade *vs.* low grade	1.38	UP	EXP00401
hsa-miR-222-3p	TCGA_THCA	high grade *vs.* low grade	1.38	UP	EXP00402
hsa-miR-181b-2-3p	TCGA_THCA	cancer *vs.* normal	1.36	UP	EXP00396
hsa-miR-221-3p	TCGA_THCA	high grade *vs.* low grade	1.35	UP	EXP00402
hsa-miR-200b	GSE40807	cancer *vs.* normal	1.33	UP	EXP00274
hsa-miR-222-5p	TCGA_THCA	cancer *vs.* normal	1.33	UP	EXP00396
hsa-miR-493*	GSE40807	cancer *vs.* normal	1.32	UP	EXP00274
hsa-miR-338-3p	GSE40807	cancer *vs.* normal	1.32	UP	EXP00274
hsa-miR-337-3p	GSE40807	cancer *vs.* normal	1.32	UP	EXP00274
hsa-miR-31-5p	TCGA_THCA	high grade *vs.* low grade	1.29	UP	EXP00402
hsa-miR-183*	GSE40807	cancer *vs.* normal	1.28	UP	EXP00274
hsa-miR-508-3p	TCGA_THCA	cancer *vs.* normal	1.28	UP	EXP00396
hsa-miR-187-3p	TCGA_THCA	cancer *vs.* normal	1.27	UP	EXP00396
hsa-miR-205-5p	TCGA_THCA	high grade *vs.* low grade	1.25	UP	EXP00402
hsa-miR-146b-5p	TCGA_THCA	high grade *vs.* low grade	1.24	UP	EXP00400
hsa-miR-127-3p	TCGA_THCA	high grade *vs.* low grade	1.24	UP	EXP00402
hsa-miR-146b-3p	TCGA_THCA	high grade *vs.* low grade	1.23	UP	EXP00400
hsa-miR-31-3p	TCGA_THCA	high grade *vs.* low grade	1.19	UP	EXP00402
hsa-miR-369-3p	GSE40807	cancer *vs.* normal	1.18	UP	EXP00274
hsa-miR-503-5p	TCGA_THCA	cancer *vs.* normal	1.16	UP	EXP00396
hsa-miR-31-5p	TCGA_THCA	high grade *vs.* low grade	1.13	UP	EXP00401
hsa-miR-3065-3p	TCGA_THCA	cancer *vs.* normal	1.11	UP	EXP00396
hsa-miR-31-3p	TCGA_THCA	high grade *vs.* low grade	1.09	UP	EXP00401
hsa-miR-551b-3p	TCGA_THCA	high grade *vs.* low grade	1.09	UP	EXP00402
hsa-miR-127-5p	TCGA_THCA	high grade *vs.* low grade	1.08	UP	EXP00401
hsa-miR-221-3p	TCGA_THCA	high grade *vs.* low grade	1.05	UP	EXP00401
hsa-miR-134-5p	TCGA_THCA	high grade *vs.* low grade	1.05	UP	EXP00402
hsa-miR-379-5p	TCGA_THCA	high grade *vs.* low grade	1.05	UP	EXP00402
hsa-miR-377*	GSE40807	cancer *vs.* normal	1.04	UP	EXP00274
hsa-miR-199a-5p	TCGA_THCA	high grade *vs.* low grade	1.04	UP	EXP00401
hsa-miR-222-3p	TCGA_THCA	high grade *vs.* low grade	1.03	UP	EXP00401
hsa-miR-23b*	GSE40807	cancer *vs.* normal	1.01	UP	EXP00274
hsa-miR-181d-5p	TCGA_THCA	cancer *vs.* normal	1.01	UP	EXP00396
hsa-miR-136-5p	TCGA_THCA	high grade *vs.* low grade	1	UP	EXP00402

hsa-miR-199a-5p	TCGA_THCA	high grade *vs*. low grade	1	UP	EXP00402
hsa-miR-1307-5p	TCGA_THCA	cancer *vs*. normal	0.99	UP	EXP00396
hsa-miR-3065-5p	TCGA_THCA	cancer *vs*. normal	0.98	UP	EXP00396
hsa-miR-421	GSE40807	cancer *vs*. normal	0.97	UP	EXP00274
hsa-miR-181a-5p	TCGA_THCA	cancer *vs*. normal	0.97	UP	EXP00396
hsa-miR-181c-3p	TCGA_THCA	cancer *vs*. normal	0.96	UP	EXP00396
hsa-miR-744-5p	TCGA_THCA	cancer *vs*. normal	0.96	UP	EXP00396
hsa-miR-345-5p	TCGA_THCA	high grade *vs*. low grade	0.96	UP	EXP00398
hsa-miR-127-3p	TCGA_THCA	high grade *vs*. low grade	0.96	UP	EXP00401
hsa-miR-21-5p	TCGA_THCA	high grade *vs*. low grade	0.95	UP	EXP00402
hsa-miR-214-5p	TCGA_THCA	high grade *vs*. low grade	0.95	UP	EXP00402
hsa-miR-96-5p	TCGA_THCA	cancer *vs*. normal	0.94	UP	EXP00396
hsa-miR-675-3p	TCGA_THCA	high grade *vs*. low grade	0.94	UP	EXP00402
hsa-miR-29c*	GSE40807	cancer *vs*. normal	0.93	UP	EXP00274
hsa-miR-214-5p	TCGA_THCA	high grade *vs*. low grade	0.93	UP	EXP00401
hsa-miR-181b-3p	TCGA_THCA	cancer *vs*. normal	0.92	UP	EXP00396
hsa-miR-1247-3p	TCGA_THCA	high grade *vs*. low grade	0.92	UP	EXP00401
hsa-miR-514a-3p	TCGA_THCA	cancer *vs*. normal	0.91	UP	EXP00396
hsa-miR-199b-3p	TCGA_THCA	high grade *vs*. low grade	0.91	UP	EXP00401
hsa-miR-181a-3p	TCGA_THCA	cancer *vs*. normal	0.9	UP	EXP00396
hsa-miR-199a-3p	TCGA_THCA	high grade *vs*. low grade	0.9	UP	EXP00401
hsa-let-7e	GSE40807	cancer *vs*. normal	0.86	UP	EXP00274
hsa-miR-224-5p	TCGA_THCA	cancer *vs*. normal	0.86	UP	EXP00396
hsa-miR-424-3p	TCGA_THCA	cancer *vs*. normal	0.86	UP	EXP00396
hsa-miR-223-3p	TCGA_THCA	high grade *vs*. low grade	0.86	UP	EXP00402
hsa-miR-204-5p	TCGA_THCA	high grade *vs*. low grade	0.85	UP	EXP00398
hsa-miR-134-5p	TCGA_THCA	high grade *vs*. low grade	0.85	UP	EXP00401
hsa-miR-381-3p	TCGA_THCA	high grade *vs*. low grade	0.85	UP	EXP00402
hsa-miR-29b-3p	TCGA_THCA	cancer *vs*. normal	0.82	UP	EXP00396
hsa-miR-21-3p	TCGA_THCA	high grade *vs*. low grade	0.82	UP	EXP00402
hsa-miR-450b-5p	TCGA_THCA	cancer *vs*. normal	0.81	UP	EXP00396
hsa-miR-21-3p	TCGA_THCA	high grade *vs*. low grade	0.81	UP	EXP00401
hsa-miR-379-5p	TCGA_THCA	high grade *vs*. low grade	0.81	UP	EXP00401
hsa-miR-146a-5p	TCGA_THCA	high grade *vs*. low grade	0.81	UP	EXP00402
hsa-miR-199b-3p	TCGA_THCA	high grade *vs*. low grade	0.81	UP	EXP00402
hsa-miR-127-5p	TCGA_THCA	high grade *vs*. low grade	0.8	UP	EXP00400
hsa-miR-136-5p	TCGA_THCA	high grade *vs*. low grade	0.8	UP	EXP00401
hsa-miR-146a-5p	TCGA_THCA	high grade *vs*. low grade	0.8	UP	EXP00401
hsa-miR-142-3p	TCGA_THCA	high grade *vs*. low grade	0.79	UP	EXP00401
hsa-miR-199a-3p	TCGA_THCA	high grade *vs*. low grade	0.79	UP	EXP00402
hsa-miR-183-5p	TCGA_THCA	high grade *vs*. low grade	0.78	UP	EXP00398
hsa-miR-21-5p	TCGA_THCA	high grade *vs*. low grade	0.78	UP	EXP00401
hsa-miR-337-3p	TCGA_THCA	high grade *vs*. low grade	0.78	UP	EXP00402
hsa-miR-210-3p	TCGA_THCA	high grade *vs*. low grade	0.78	UP	EXP00402
hsa-miR-21	GSE40807	cancer *vs*. normal	0.77	UP	EXP00274
hsa-miR-656	GSE40807	cancer *vs*. normal	0.77	UP	EXP00274
hsa-miR-4787-3p	TCGA_THCA	cancer *vs*. normal	0.77	UP	EXP00396
hsa-miR-6842-3p	TCGA_THCA	cancer *vs*. normal	0.77	UP	EXP00396
hsa-miR-891a-5p	TCGA_THCA	cancer *vs*. normal	0.77	UP	EXP00396
hsa-miR-15a-5p	TCGA_THCA	cancer *vs*. normal	0.75	UP	EXP00396
hsa-miR-183-5p	TCGA_THCA	cancer *vs*. normal	0.75	UP	EXP00396

hsa-miR-382-5p	TCGA_THCA	high grade *vs.* low grade	0.75	UP	EXP00402
hsa-miR-199b-5p	TCGA_THCA	high grade *vs.* low grade	0.74	UP	EXP00399
hsa-miR-23b	GSE40807	cancer *vs.* normal	0.73	UP	EXP00274
hsa-miR-675-3p	TCGA_THCA	high grade *vs.* low grade	0.73	UP	EXP00401
hsa-miR-335-3p	TCGA_THCA	cancer *vs.* normal	0.72	UP	EXP00396
hsa-miR-127-3p	TCGA_THCA	high grade *vs.* low grade	0.72	UP	EXP00400
hsa-miR-508-3p	TCGA_THCA	high grade *vs.* low grade	0.72	UP	EXP00401
hsa-miR-3613-5p	TCGA_THCA	cancer *vs.* normal	0.71	UP	EXP00396
hsa-miR-99b	GSE40807	cancer *vs.* normal	0.7	UP	EXP00274
hsa-miR-509-3p	TCGA_THCA	cancer *vs.* normal	0.7	UP	EXP00396
hsa-miR-874-3p	TCGA_THCA	high grade *vs.* low grade	0.7	UP	EXP00398
hsa-miR-210-3p	TCGA_THCA	high grade *vs.* low grade	0.7	UP	EXP00401
hsa-miR-92b-3p	TCGA_THCA	cancer *vs.* normal	0.69	UP	EXP00396
hsa-miR-152-3p	TCGA_THCA	high grade *vs.* low grade	0.68	UP	EXP00398
hsa-miR-136-3p	TCGA_THCA	high grade *vs.* low grade	0.68	UP	EXP00402
hsa-let-7e-5p	TCGA_THCA	cancer *vs.* normal	0.67	UP	EXP00396
hsa-miR-1251-5p	TCGA_THCA	cancer *vs.* normal	0.67	UP	EXP00396
hsa-miR-16-1-3p	TCGA_THCA	cancer *vs.* normal	0.66	UP	EXP00396
hsa-miR-203a-3p	TCGA_THCA	high grade *vs.* low grade	0.66	UP	EXP00401
hsa-miR-150-5p	TCGA_THCA	high grade *vs.* low grade	0.66	UP	EXP00401
hsa-miR-222-5p	TCGA_THCA	high grade *vs.* low grade	0.66	UP	EXP00402
hsa-miR-654-3p	TCGA_THCA	high grade *vs.* low grade	0.66	UP	EXP00402
hsa-miR-382-5p	TCGA_THCA	high grade *vs.* low grade	0.65	UP	EXP00401
hsa-miR-514a-3p	TCGA_THCA	high grade *vs.* low grade	0.65	UP	EXP00402
hsa-miR-27b-5p	TCGA_THCA	cancer *vs.* normal	0.64	UP	EXP00396
hsa-miR-450a-5p	TCGA_THCA	cancer *vs.* normal	0.64	UP	EXP00396
hsa-miR-508-3p	TCGA_THCA	high grade *vs.* low grade	0.64	UP	EXP00402
hsa-let-7e-3p	TCGA_THCA	cancer *vs.* normal	0.63	UP	EXP00396
hsa-miR-142-5p	TCGA_THCA	high grade *vs.* low grade	0.63	UP	EXP00401
hsa-miR-141-3p	TCGA_THCA	cancer *vs.* normal	0.62	UP	EXP00396
hsa-miR-6715a-3p	TCGA_THCA	high grade *vs.* low grade	0.62	UP	EXP00398
hsa-miR-136-3p	TCGA_THCA	high grade *vs.* low grade	0.62	UP	EXP00401
hsa-miR-223-3p	TCGA_THCA	high grade *vs.* low grade	0.62	UP	EXP00401
hsa-miR-29b	GSE40807	cancer *vs.* normal	0.61	UP	EXP00274
hsa-miR-3200-3p	TCGA_THCA	cancer *vs.* normal	0.6	UP	EXP00396
hsa-miR-337-3p	TCGA_THCA	high grade *vs.* low grade	0.59	UP	EXP00401
hsa-miR-381-3p	TCGA_THCA	high grade *vs.* low grade	0.59	UP	EXP00401
hsa-miR-142-5p	TCGA_THCA	high grade *vs.* low grade	0.59	UP	EXP00402
hsa-miR-542-3p	TCGA_THCA	high grade *vs.* low grade	0.57	UP	EXP00398
hsa-miR-889-3p	TCGA_THCA	high grade *vs.* low grade	0.57	UP	EXP00402
hsa-miR-27b	GSE40807	cancer *vs.* normal	0.56	UP	EXP00274
hsa-miR-99b-5p	TCGA_THCA	cancer *vs.* normal	0.56	UP	EXP00396
hsa-miR-324-3p	TCGA_THCA	cancer *vs.* normal	0.56	UP	EXP00396
hsa-miR-345-3p	TCGA_THCA	high grade *vs.* low grade	0.56	UP	EXP00398
hsa-miR-139-3p	TCGA_THCA	high grade *vs.* low grade	0.56	UP	EXP00398
hsa-miR-1251-5p	TCGA_THCA	high grade *vs.* low grade	0.56	UP	EXP00398
hsa-miR-376c-3p	TCGA_THCA	high grade *vs.* low grade	0.56	UP	EXP00402
hsa-miR-758-3p	TCGA_THCA	high grade *vs.* low grade	0.56	UP	EXP00402
hsa-miR-141	GSE40807	cancer *vs.* normal	0.55	UP	EXP00274
hsa-miR-1307-3p	TCGA_THCA	cancer *vs.* normal	0.55	UP	EXP00396
hsa-miR-29a-5p	TCGA_THCA	cancer *vs.* normal	0.55	UP	EXP00396

hsa-miR-30d	GSE40807	cancer *vs.* normal	0.54	UP	EXP00274
hsa-miR-181a	GSE40807	cancer *vs.* normal	0.54	UP	EXP00274
hsa-miR-99b-3p	TCGA_THCA	cancer *vs.* normal	0.54	UP	EXP00396
hsa-miR-136-5p	TCGA_THCA	high grade *vs.* low grade	0.54	UP	EXP00400
hsa-miR-675-3p	TCGA_THCA	high grade *vs.* low grade	0.54	UP	EXP00400
hsa-miR-493-5p	TCGA_THCA	high grade *vs.* low grade	0.54	UP	EXP00402
hsa-miR-410-3p	TCGA_THCA	high grade *vs.* low grade	0.54	UP	EXP00402
hsa-miR-200c	GSE40807	cancer *vs.* normal	0.53	UP	EXP00274
hsa-miR-200c-3p	TCGA_THCA	cancer *vs.* normal	0.53	UP	EXP00396
hsa-miR-450b-5p	TCGA_THCA	high grade *vs.* low grade	0.53	UP	EXP00398
hsa-miR-1247-5p	TCGA_THCA	high grade *vs.* low grade	0.53	UP	EXP00401
hsa-miR-423-3p	TCGA_THCA	cancer *vs.* normal	0.52	UP	EXP00396
hsa-miR-125a-5p	TCGA_THCA	cancer *vs.* normal	0.52	UP	EXP00396
hsa-miR-429	TCGA_THCA	cancer *vs.* normal	0.52	UP	EXP00396
hsa-miR-511-5p	TCGA_THCA	high grade *vs.* low grade	0.52	UP	EXP00401
hsa-miR-155-5p	TCGA_THCA	high grade *vs.* low grade	0.52	UP	EXP00401
hsa-miR-19b-1-5p	TCGA_THCA	high grade *vs.* low grade	0.52	UP	EXP00402
hsa-miR-425-5p	TCGA_THCA	high grade *vs.* low grade	0.52	UP	EXP00402
hsa-miR-29b-3p	TCGA_THCA	high grade *vs.* low grade	0.52	UP	EXP00402
hsa-miR-511-5p	TCGA_THCA	high grade *vs.* low grade	0.52	UP	EXP00402
hsa-miR-203a-3p	TCGA_THCA	high grade *vs.* low grade	0.52	UP	EXP00402
hsa-let-7d-3p	TCGA_THCA	cancer *vs.* normal	0.51	UP	EXP00396
hsa-miR-744-3p	TCGA_THCA	cancer *vs.* normal	0.51	UP	EXP00396
hsa-miR-514a-3p	TCGA_THCA	high grade *vs.* low grade	0.51	UP	EXP00400
hsa-miR-9-5p	TCGA_THCA	high grade *vs.* low grade	0.51	UP	EXP00401
hsa-miR-409-5p	TCGA_THCA	high grade *vs.* low grade	0.51	UP	EXP00402
hsa-miR-182-5p	TCGA_THCA	cancer *vs.* normal	0.5	UP	EXP00396
hsa-miR-30c-2-3p	TCGA_THCA	high grade *vs.* low grade	0.5	UP	EXP00398
hsa-miR-381-3p	TCGA_THCA	high grade *vs.* low grade	0.5	UP	EXP00400
hsa-miR-514a-3p	TCGA_THCA	high grade *vs.* low grade	0.5	UP	EXP00401
hsa-miR-671-5p	TCGA_THCA	cancer *vs.* normal	0.49	UP	EXP00396
hsa-miR-508-5p	TCGA_THCA	cancer *vs.* normal	0.49	UP	EXP00396
hsa-miR-629-5p	TCGA_THCA	cancer *vs.* normal	0.49	UP	EXP00396
hsa-miR-182-5p	TCGA_THCA	high grade *vs.* low grade	0.49	UP	EXP00398
hsa-miR-139-5p	TCGA_THCA	high grade *vs.* low grade	0.49	UP	EXP00398
hsa-miR-654-3p	TCGA_THCA	high grade *vs.* low grade	0.48	UP	EXP00400
hsa-miR-19a-3p	TCGA_THCA	high grade *vs.* low grade	0.48	UP	EXP00401
hsa-miR-592	TCGA_THCA	cancer *vs.* normal	0.47	UP	EXP00396
hsa-miR-382-5p	TCGA_THCA	high grade *vs.* low grade	0.47	UP	EXP00400
hsa-miR-337-3p	TCGA_THCA	high grade *vs.* low grade	0.47	UP	EXP00400
hsa-miR-409-3p	TCGA_THCA	high grade *vs.* low grade	0.47	UP	EXP00402
hsa-miR-19a-3p	TCGA_THCA	high grade *vs.* low grade	0.47	UP	EXP00402
hsa-miR-24	GSE40807	cancer *vs.* normal	0.46	UP	EXP00274
hsa-miR-324-5p	TCGA_THCA	cancer *vs.* normal	0.46	UP	EXP00396
hsa-miR-132-3p	TCGA_THCA	cancer *vs.* normal	0.46	UP	EXP00396
hsa-miR-1180-3p	TCGA_THCA	high grade *vs.* low grade	0.46	UP	EXP00398
hsa-miR-154-5p	TCGA_THCA	high grade *vs.* low grade	0.46	UP	EXP00401
hsa-miR-369-3p	TCGA_THCA	high grade *vs.* low grade	0.46	UP	EXP00402
hsa-miR-3934-3p	TCGA_THCA	cancer *vs.* normal	0.45	UP	EXP00396
hsa-miR-19b-3p	TCGA_THCA	high grade *vs.* low grade	0.45	UP	EXP00401
hsa-miR-369-3p	TCGA_THCA	high grade *vs.* low grade	0.45	UP	EXP00401

hsa-miR-509-3p	TCGA_THCA	high grade *vs.* low grade	0.45	UP	EXP00401
hsa-miR-654-3p	TCGA_THCA	high grade *vs.* low grade	0.45	UP	EXP00401
hsa-miR-154-5p	TCGA_THCA	high grade *vs.* low grade	0.45	UP	EXP00402
hsa-miR-149-5p	TCGA_THCA	cancer *vs.* normal	0.44	UP	EXP00396
hsa-miR-493-5p	TCGA_THCA	high grade *vs.* low grade	0.44	UP	EXP00401
hsa-miR-29b-1-5p	TCGA_THCA	cancer *vs.* normal	0.43	UP	EXP00396
hsa-miR-508-3p	TCGA_THCA	high grade *vs.* low grade	0.43	UP	EXP00399
hsa-miR-5683	TCGA_THCA	high grade *vs.* low grade	0.43	UP	EXP00402
hsa-miR-509-3p	TCGA_THCA	high grade *vs.* low grade	0.43	UP	EXP00402
hsa-miR-758-5p	TCGA_THCA	high grade *vs.* low grade	0.43	UP	EXP00402
hsa-miR-5010-3p	TCGA_THCA	cancer *vs.* normal	0.42	UP	EXP00396
hsa-miR-542-3p	TCGA_THCA	cancer *vs.* normal	0.42	UP	EXP00396
hsa-miR-3200-3p	TCGA_THCA	high grade *vs.* low grade	0.42	UP	EXP00398
hsa-miR-96-5p	TCGA_THCA	high grade *vs.* low grade	0.42	UP	EXP00398
hsa-miR-425-5p	TCGA_THCA	high grade *vs.* low grade	0.42	UP	EXP00400
hsa-miR-574-3p	TCGA_THCA	high grade *vs.* low grade	0.42	UP	EXP00401
hsa-miR-20a-5p	TCGA_THCA	high grade *vs.* low grade	0.42	UP	EXP00401
hsa-miR-425-5p	TCGA_THCA	high grade *vs.* low grade	0.42	UP	EXP00401
hsa-miR-29a-5p	TCGA_THCA	high grade *vs.* low grade	0.42	UP	EXP00402
hsa-miR-30b-3p	TCGA_THCA	cancer *vs.* normal	0.41	UP	EXP00396
hsa-miR-33b-5p	TCGA_THCA	cancer *vs.* normal	0.41	UP	EXP00396
hsa-miR-331-3p	TCGA_THCA	cancer *vs.* normal	0.41	UP	EXP00396
hsa-miR-3074-5p	TCGA_THCA	high grade *vs.* low grade	0.41	UP	EXP00398
hsa-miR-5683	TCGA_THCA	high grade *vs.* low grade	0.41	UP	EXP00400
hsa-miR-758-3p	TCGA_THCA	high grade *vs.* low grade	0.41	UP	EXP00400
hsa-miR-455-5p	TCGA_THCA	high grade *vs.* low grade	0.41	UP	EXP00401
hsa-miR-20a-5p	TCGA_THCA	high grade *vs.* low grade	0.41	UP	EXP00402
hsa-miR-135b-3p	TCGA_THCA	cancer *vs.* normal	0.4	UP	EXP00396
hsa-miR-24-1-5p	TCGA_THCA	cancer *vs.* normal	0.4	UP	EXP00396
hsa-miR-328-3p	TCGA_THCA	cancer *vs.* normal	0.4	UP	EXP00396
hsa-miR-184	TCGA_THCA	cancer *vs.* normal	0.4	UP	EXP00396
hsa-miR-424-5p	TCGA_THCA	cancer *vs.* normal	0.4	UP	EXP00396
hsa-miR-664a-5p	TCGA_THCA	high grade *vs.* low grade	0.4	UP	EXP00398
hsa-miR-493-5p	TCGA_THCA	high grade *vs.* low grade	0.4	UP	EXP00400
hsa-miR-136-3p	TCGA_THCA	high grade *vs.* low grade	0.4	UP	EXP00400
hsa-miR-376c-3p	TCGA_THCA	high grade *vs.* low grade	0.4	UP	EXP00401
hsa-miR-889-3p	TCGA_THCA	high grade *vs.* low grade	0.4	UP	EXP00401
hsa-miR-29a-3p	TCGA_THCA	high grade *vs.* low grade	0.4	UP	EXP00402
hsa-miR-200b-5p	TCGA_THCA	cancer *vs.* normal	0.39	UP	EXP00396
hsa-let-7e-5p	TCGA_THCA	high grade *vs.* low grade	0.39	UP	EXP00398
hsa-miR-889-3p	TCGA_THCA	high grade *vs.* low grade	0.39	UP	EXP00400
hsa-miR-411-5p	TCGA_THCA	high grade *vs.* low grade	0.39	UP	EXP00402
hsa-miR-132-5p	TCGA_THCA	cancer *vs.* normal	0.38	UP	EXP00396
hsa-miR-126-5p	TCGA_THCA	high grade *vs.* low grade	0.38	UP	EXP00398
hsa-miR-542-5p	TCGA_THCA	high grade *vs.* low grade	0.38	UP	EXP00398
hsa-miR-214-5p	TCGA_THCA	high grade *vs.* low grade	0.38	UP	EXP00399
hsa-miR-409-3p	TCGA_THCA	high grade *vs.* low grade	0.38	UP	EXP00400
hsa-miR-5586-5p	TCGA_THCA	high grade *vs.* low grade	0.38	UP	EXP00401
hsa-miR-5010-3p	TCGA_THCA	high grade *vs.* low grade	0.38	UP	EXP00402
hsa-miR-6843-3p	TCGA_THCA	cancer *vs.* normal	0.37	UP	EXP00396
hsa-miR-509-3-5p	TCGA_THCA	cancer *vs.* normal	0.37	UP	EXP00396

hsa-miR-7-1-3p	TCGA_THCA	cancer *vs.* normal	0.37	UP	EXP00396
hsa-miR-542-5p	TCGA_THCA	cancer *vs.* normal	0.37	UP	EXP00396
hsa-miR-212-3p	TCGA_THCA	cancer *vs.* normal	0.37	UP	EXP00396
hsa-miR-382-5p	TCGA_THCA	high grade *vs.* low grade	0.37	UP	EXP00399
hsa-miR-19b-1-5p	TCGA_THCA	high grade *vs.* low grade	0.37	UP	EXP00401
hsa-miR-758-3p	TCGA_THCA	high grade *vs.* low grade	0.37	UP	EXP00401
hsa-miR-29b-3p	TCGA_THCA	high grade *vs.* low grade	0.37	UP	EXP00401
hsa-miR-539-5p	TCGA_THCA	high grade *vs.* low grade	0.37	UP	EXP00402
hsa-miR-19b-3p	TCGA_THCA	high grade *vs.* low grade	0.37	UP	EXP00402
hsa-miR-30d-5p	TCGA_THCA	cancer *vs.* normal	0.36	UP	EXP00396
hsa-miR-196a-5p	TCGA_THCA	cancer *vs.* normal	0.36	UP	EXP00396
hsa-miR-331-3p	TCGA_THCA	high grade *vs.* low grade	0.36	UP	EXP00398
hsa-miR-514a-3p	TCGA_THCA	high grade *vs.* low grade	0.36	UP	EXP00399
hsa-miR-409-5p	TCGA_THCA	high grade *vs.* low grade	0.36	UP	EXP00400
hsa-miR-135b-5p	TCGA_THCA	high grade *vs.* low grade	0.36	UP	EXP00400
hsa-miR-574-3p	TCGA_THCA	high grade *vs.* low grade	0.36	UP	EXP00402
hsa-miR-1251-3p	TCGA_THCA	cancer *vs.* normal	0.35	UP	EXP00396
hsa-miR-324-3p	TCGA_THCA	high grade *vs.* low grade	0.35	UP	EXP00398
hsa-miR-3912-3p	TCGA_THCA	high grade *vs.* low grade	0.35	UP	EXP00398
hsa-miR-98-5p	TCGA_THCA	high grade *vs.* low grade	0.35	UP	EXP00398
hsa-miR-200c-5p	TCGA_THCA	high grade *vs.* low grade	0.35	UP	EXP00398
hsa-miR-190a-5p	TCGA_THCA	high grade *vs.* low grade	0.35	UP	EXP00398
hsa-miR-30c-5p	TCGA_THCA	high grade *vs.* low grade	0.35	UP	EXP00398
hsa-miR-17-5p	TCGA_THCA	high grade *vs.* low grade	0.35	UP	EXP00401
hsa-miR-671-3p	TCGA_THCA	cancer *vs.* normal	0.34	UP	EXP00396
hsa-miR-598-3p	TCGA_THCA	cancer *vs.* normal	0.34	UP	EXP00396
hsa-miR-501-3p	TCGA_THCA	cancer *vs.* normal	0.34	UP	EXP00396
hsa-miR-361-5p	TCGA_THCA	high grade *vs.* low grade	0.34	UP	EXP00398
hsa-miR-22-3p	TCGA_THCA	high grade *vs.* low grade	0.34	UP	EXP00398
hsa-miR-744-5p	TCGA_THCA	high grade *vs.* low grade	0.34	UP	EXP00398
hsa-miR-136-3p	TCGA_THCA	high grade *vs.* low grade	0.34	UP	EXP00399
hsa-miR-129-5p	TCGA_THCA	high grade *vs.* low grade	0.34	UP	EXP00399
hsa-miR-376c-3p	TCGA_THCA	high grade *vs.* low grade	0.34	UP	EXP00400
hsa-miR-6854-5p	TCGA_THCA	cancer *vs.* normal	0.33	UP	EXP00396
hsa-miR-103a-3p	TCGA_THCA	cancer *vs.* normal	0.33	UP	EXP00396
hsa-miR-6892-5p	TCGA_THCA	cancer *vs.* normal	0.33	UP	EXP00396
hsa-miR-148b-3p	TCGA_THCA	high grade *vs.* low grade	0.33	UP	EXP00398
hsa-miR-3677-3p	TCGA_THCA	high grade *vs.* low grade	0.33	UP	EXP00398
hsa-miR-328-3p	TCGA_THCA	high grade *vs.* low grade	0.33	UP	EXP00398
hsa-miR-92b-3p	TCGA_THCA	high grade *vs.* low grade	0.33	UP	EXP00400
hsa-miR-758-5p	TCGA_THCA	high grade *vs.* low grade	0.33	UP	EXP00401
hsa-miR-342-3p	TCGA_THCA	high grade *vs.* low grade	0.33	UP	EXP00401
hsa-miR-33b-5p	TCGA_THCA	high grade *vs.* low grade	0.33	UP	EXP00402
hsa-miR-29c	GSE40807	cancer *vs.* normal	0.32	UP	EXP00274
hsa-miR-30e-3p	TCGA_THCA	high grade *vs.* low grade	0.32	UP	EXP00398
hsa-miR-425-5p	TCGA_THCA	high grade *vs.* low grade	0.32	UP	EXP00399
hsa-miR-539-5p	TCGA_THCA	high grade *vs.* low grade	0.32	UP	EXP00400
hsa-miR-409-3p	TCGA_THCA	high grade *vs.* low grade	0.32	UP	EXP00401
hsa-miR-214-3p	TCGA_THCA	high grade *vs.* low grade	0.32	UP	EXP00402
hsa-miR-493-3p	TCGA_THCA	high grade *vs.* low grade	0.32	UP	EXP00402
hsa-miR-485-3p	TCGA_THCA	high grade *vs.* low grade	0.32	UP	EXP00402

hsa-miR-491-3p	TCGA_THCA	cancer *vs.* normal	0.31	UP	EXP00396
hsa-miR-664b-3p	TCGA_THCA	cancer *vs.* normal	0.31	UP	EXP00396
hsa-miR-135b-5p	TCGA_THCA	cancer *vs.* normal	0.31	UP	EXP00396
hsa-miR-4662a-5p	TCGA_THCA	high grade *vs.* low grade	0.31	UP	EXP00398
hsa-miR-584-5p	TCGA_THCA	high grade *vs.* low grade	0.31	UP	EXP00398
hsa-miR-493-5p	TCGA_THCA	high grade *vs.* low grade	0.31	UP	EXP00399
hsa-miR-24-2-5p	TCGA_THCA	high grade *vs.* low grade	0.31	UP	EXP00402
hsa-miR-17-5p	TCGA_THCA	high grade *vs.* low grade	0.31	UP	EXP00402
hsa-miR-944	TCGA_THCA	cancer *vs.* normal	0.3	UP	EXP00396
hsa-miR-346	TCGA_THCA	cancer *vs.* normal	0.3	UP	EXP00396
hsa-miR-452-5p	TCGA_THCA	cancer *vs.* normal	0.3	UP	EXP00396
hsa-miR-628-5p	TCGA_THCA	high grade *vs.* low grade	0.3	UP	EXP00398
hsa-miR-95-3p	TCGA_THCA	high grade *vs.* low grade	0.3	UP	EXP00398
hsa-miR-15a-5p	TCGA_THCA	high grade *vs.* low grade	0.3	UP	EXP00402
hsa-miR-1277-3p	TCGA_THCA	cancer *vs.* normal	0.29	UP	EXP00396
hsa-miR-1226-3p	TCGA_THCA	cancer *vs.* normal	0.29	UP	EXP00396
hsa-miR-188-5p	TCGA_THCA	cancer *vs.* normal	0.29	UP	EXP00396
hsa-miR-181c-5p	TCGA_THCA	cancer *vs.* normal	0.29	UP	EXP00396
hsa-miR-940	TCGA_THCA	high grade *vs.* low grade	0.29	UP	EXP00398
hsa-miR-935	TCGA_THCA	high grade *vs.* low grade	0.29	UP	EXP00398
hsa-miR-185-5p	TCGA_THCA	high grade *vs.* low grade	0.29	UP	EXP00398
hsa-miR-671-3p	TCGA_THCA	high grade *vs.* low grade	0.29	UP	EXP00398
hsa-miR-151a-3p	TCGA_THCA	high grade *vs.* low grade	0.29	UP	EXP00398
hsa-miR-3613-5p	TCGA_THCA	high grade *vs.* low grade	0.29	UP	EXP00400
hsa-miR-214-3p	TCGA_THCA	high grade *vs.* low grade	0.29	UP	EXP00401
hsa-miR-411-5p	TCGA_THCA	high grade *vs.* low grade	0.29	UP	EXP00401
hsa-miR-92a-3p	TCGA_THCA	high grade *vs.* low grade	0.29	UP	EXP00402
hsa-miR-495-3p	TCGA_THCA	high grade *vs.* low grade	0.29	UP	EXP00402
hsa-miR-937-3p	TCGA_THCA	cancer *vs.* normal	0.28	UP	EXP00396
hsa-miR-4746-5p	TCGA_THCA	cancer *vs.* normal	0.28	UP	EXP00396
hsa-miR-340-3p	TCGA_THCA	cancer *vs.* normal	0.28	UP	EXP00396
hsa-miR-27b-3p	TCGA_THCA	cancer *vs.* normal	0.28	UP	EXP00396
hsa-miR-362-5p	TCGA_THCA	cancer *vs.* normal	0.28	UP	EXP00396
hsa-miR-585-3p	TCGA_THCA	high grade *vs.* low grade	0.28	UP	EXP00398
hsa-miR-23b-3p	TCGA_THCA	high grade *vs.* low grade	0.28	UP	EXP00398
hsa-miR-509-3p	TCGA_THCA	high grade *vs.* low grade	0.28	UP	EXP00399
hsa-miR-33b-5p	TCGA_THCA	high grade *vs.* low grade	0.28	UP	EXP00400
hsa-miR-369-3p	TCGA_THCA	high grade *vs.* low grade	0.28	UP	EXP00400
hsa-miR-5010-3p	TCGA_THCA	high grade *vs.* low grade	0.28	UP	EXP00401
hsa-miR-493-3p	TCGA_THCA	high grade *vs.* low grade	0.28	UP	EXP00401
hsa-miR-607	GSE62054	subtype1 *vs.* substype2	0.27	UP	EXP00298
hsa-miR-151a-5p	TCGA_THCA	cancer *vs.* normal	0.27	UP	EXP00396
hsa-miR-3170	TCGA_THCA	cancer *vs.* normal	0.27	UP	EXP00396
hsa-miR-4728-3p	TCGA_THCA	cancer *vs.* normal	0.27	UP	EXP00396
hsa-miR-503-3p	TCGA_THCA	cancer *vs.* normal	0.27	UP	EXP00396
hsa-miR-2355-5p	TCGA_THCA	cancer *vs.* normal	0.27	UP	EXP00396
hsa-miR-224-3p	TCGA_THCA	cancer *vs.* normal	0.27	UP	EXP00396
hsa-miR-30c-1-3p	TCGA_THCA	high grade *vs.* low grade	0.27	UP	EXP00398
hsa-miR-1275	TCGA_THCA	high grade *vs.* low grade	0.27	UP	EXP00398
hsa-miR-676-3p	TCGA_THCA	high grade *vs.* low grade	0.27	UP	EXP00398
hsa-miR-532-3p	TCGA_THCA	high grade *vs.* low grade	0.27	UP	EXP00398

hsa-miR-369-3p	TCGA_THCA	high grade *vs.* low grade	0.27	UP	EXP00399
hsa-miR-125b-5p	TCGA_THCA	high grade *vs.* low grade	0.27	UP	EXP00401
hsa-miR-23c	TCGA_THCA	cancer *vs.* normal	0.26	UP	EXP00396
hsa-miR-128-3p	TCGA_THCA	high grade *vs.* low grade	0.26	UP	EXP00398
hsa-miR-1287-5p	TCGA_THCA	high grade *vs.* low grade	0.26	UP	EXP00398
hsa-miR-1307-3p	TCGA_THCA	high grade *vs.* low grade	0.26	UP	EXP00398
hsa-miR-495-3p	TCGA_THCA	high grade *vs.* low grade	0.26	UP	EXP00401
hsa-miR-508-5p	TCGA_THCA	high grade *vs.* low grade	0.26	UP	EXP00401
hsa-miR-590-5p	TCGA_THCA	cancer *vs.* normal	0.25	UP	EXP00396
hsa-miR-23b-3p	TCGA_THCA	cancer *vs.* normal	0.25	UP	EXP00396
hsa-miR-629-3p	TCGA_THCA	cancer *vs.* normal	0.25	UP	EXP00396
hsa-let-7e-3p	TCGA_THCA	high grade *vs.* low grade	0.25	UP	EXP00398
hsa-miR-4668-3p	TCGA_THCA	high grade *vs.* low grade	0.25	UP	EXP00401
hsa-miR-625-3p	TCGA_THCA	high grade *vs.* low grade	0.25	UP	EXP00401
hsa-miR-16-2-3p	TCGA_THCA	high grade *vs.* low grade	0.25	UP	EXP00402
hsa-miR-34a-3p	TCGA_THCA	cancer *vs.* normal	0.24	UP	EXP00396
hsa-miR-659-5p	TCGA_THCA	cancer *vs.* normal	0.24	UP	EXP00396
hsa-miR-877-5p	TCGA_THCA	cancer *vs.* normal	0.24	UP	EXP00396
hsa-miR-30d-3p	TCGA_THCA	cancer *vs.* normal	0.24	UP	EXP00396
hsa-miR-1224-5p	TCGA_THCA	cancer *vs.* normal	0.24	UP	EXP00396
hsa-miR-3605-3p	TCGA_THCA	cancer *vs.* normal	0.24	UP	EXP00396
hsa-miR-505-5p	TCGA_THCA	cancer *vs.* normal	0.24	UP	EXP00396
hsa-miR-935	TCGA_THCA	cancer *vs.* normal	0.24	UP	EXP00396
hsa-miR-1306-5p	TCGA_THCA	cancer *vs.* normal	0.24	UP	EXP00396
hsa-miR-219a-1-3p	TCGA_THCA	high grade *vs.* low grade	0.24	UP	EXP00398
hsa-miR-874-5p	TCGA_THCA	high grade *vs.* low grade	0.24	UP	EXP00398
hsa-miR-508-5p	TCGA_THCA	high grade *vs.* low grade	0.24	UP	EXP00399
hsa-miR-369-5p	TCGA_THCA	high grade *vs.* low grade	0.24	UP	EXP00402
hsa-miR-103	GSE40807	cancer *vs.* normal	0.23	UP	EXP00274
hsa-miR-506-3p	TCGA_THCA	cancer *vs.* normal	0.23	UP	EXP00396
hsa-miR-99b-5p	TCGA_THCA	high grade *vs.* low grade	0.23	UP	EXP00400
hsa-miR-944	TCGA_THCA	high grade *vs.* low grade	0.23	UP	EXP00401
hsa-miR-4668-3p	TCGA_THCA	high grade *vs.* low grade	0.23	UP	EXP00402
hsa-miR-4677-3p	TCGA_THCA	cancer *vs.* normal	0.22	UP	EXP00396
hsa-miR-125b-5p	TCGA_THCA	cancer *vs.* normal	0.22	UP	EXP00396
hsa-miR-128-1-5p	TCGA_THCA	high grade *vs.* low grade	0.22	UP	EXP00398
hsa-miR-1296-5p	TCGA_THCA	high grade *vs.* low grade	0.22	UP	EXP00398
hsa-miR-154-5p	TCGA_THCA	high grade *vs.* low grade	0.22	UP	EXP00399
hsa-miR-655-3p	TCGA_THCA	high grade *vs.* low grade	0.22	UP	EXP00400
hsa-miR-24-2-5p	TCGA_THCA	high grade *vs.* low grade	0.22	UP	EXP00401
hsa-miR-323a-3p	TCGA_THCA	high grade *vs.* low grade	0.22	UP	EXP00402
hsa-miR-655-3p	TCGA_THCA	high grade *vs.* low grade	0.22	UP	EXP00402
hsa-miR-1301-3p	TCGA_THCA	cancer *vs.* normal	0.21	UP	EXP00396
hsa-miR-3934-5p	TCGA_THCA	cancer *vs.* normal	0.21	UP	EXP00396
hsa-miR-4668-3p	TCGA_THCA	cancer *vs.* normal	0.2	UP	EXP00396
hsa-miR-26a-2-3p	TCGA_THCA	cancer *vs.* normal	0.2	UP	EXP00396
hsa-miR-369-5p	TCGA_THCA	high grade *vs.* low grade	0.2	UP	EXP00400
hsa-miR-5010-3p	TCGA_THCA	high grade *vs.* low grade	0.2	UP	EXP00400
hsa-miR-92a-3p	TCGA_THCA	high grade *vs.* low grade	0.2	UP	EXP00401
hsa-miR-625-5p	TCGA_THCA	cancer *vs.* normal	0.19	UP	EXP00396
hsa-miR-361-5p	TCGA_THCA	cancer *vs.* normal	0.19	UP	EXP00396

hsa-miR-425-3p	TCGA_THCA	cancer *vs.* normal	0.19	UP	EXP00396
hsa-miR-301a-5p	TCGA_THCA	cancer *vs.* normal	0.19	UP	EXP00396
hsa-miR-216a-5p	TCGA_THCA	cancer *vs.* normal	0.19	UP	EXP00396
hsa-miR-103a-2-5p	TCGA_THCA	cancer *vs.* normal	0.19	UP	EXP00396
hsa-miR-3677-5p	TCGA_THCA	high grade *vs.* low grade	0.19	UP	EXP00398
hsa-miR-188-5p	TCGA_THCA	high grade *vs.* low grade	0.19	UP	EXP00398
hsa-miR-296-3p	TCGA_THCA	cancer *vs.* normal	0.18	UP	EXP00396
hsa-miR-421	TCGA_THCA	high grade *vs.* low grade	0.18	UP	EXP00398
hsa-miR-188-3p	TCGA_THCA	high grade *vs.* low grade	0.18	UP	EXP00398
hsa-miR-214-3p	TCGA_THCA	high grade *vs.* low grade	0.18	UP	EXP00399
hsa-miR-6854-5p	TCGA_THCA	high grade *vs.* low grade	0.18	UP	EXP00401
hsa-miR-299-5p	TCGA_THCA	high grade *vs.* low grade	0.18	UP	EXP00402
hsa-miR-128-3p	TCGA_THCA	cancer *vs.* normal	0.17	UP	EXP00396
hsa-miR-6806-3p	TCGA_THCA	high grade *vs.* low grade	0.17	UP	EXP00398
hsa-miR-323a-3p	TCGA_THCA	high grade *vs.* low grade	0.17	UP	EXP00401
hsa-miR-369-5p	TCGA_THCA	high grade *vs.* low grade	0.17	UP	EXP00401
hsa-miR-506-3p	TCGA_THCA	high grade *vs.* low grade	0.17	UP	EXP00401
hsa-miR-154-3p	TCGA_THCA	high grade *vs.* low grade	0.17	UP	EXP00402
hsa-miR-6854-5p	TCGA_THCA	high grade *vs.* low grade	0.17	UP	EXP00402
hsa-miR-616-3p	TCGA_THCA	cancer *vs.* normal	0.16	UP	EXP00396
hsa-miR-2355-3p	TCGA_THCA	cancer *vs.* normal	0.16	UP	EXP00396
hsa-miR-769-5p	TCGA_THCA	cancer *vs.* normal	0.16	UP	EXP00396
hsa-miR-6730-5p	TCGA_THCA	cancer *vs.* normal	0.15	UP	EXP00396
hsa-miR-7706	TCGA_THCA	cancer *vs.* normal	0.15	UP	EXP00396
hsa-miR-3913-5p	TCGA_THCA	cancer *vs.* normal	0.15	UP	EXP00396
hsa-miR-3677-5p	TCGA_THCA	cancer *vs.* normal	0.15	UP	EXP00396
hsa-miR-3622a-3p	TCGA_THCA	cancer *vs.* normal	0.15	UP	EXP00396
hsa-miR-3193	TCGA_THCA	high grade *vs.* low grade	0.15	UP	EXP00398
hsa-miR-506-3p	TCGA_THCA	high grade *vs.* low grade	0.15	UP	EXP00399
hsa-miR-299-5p	TCGA_THCA	high grade *vs.* low grade	0.15	UP	EXP00400
hsa-miR-188-3p	TCGA_THCA	cancer *vs.* normal	0.14	UP	EXP00396
hsa-miR-98-3p	TCGA_THCA	cancer *vs.* normal	0.14	UP	EXP00396
hsa-miR-655-3p	TCGA_THCA	high grade *vs.* low grade	0.14	UP	EXP00399
hsa-miR-4668-3p	TCGA_THCA	high grade *vs.* low grade	0.14	UP	EXP00399
hsa-miR-323a-3p	TCGA_THCA	high grade *vs.* low grade	0.14	UP	EXP00400
hsa-miR-154-3p	TCGA_THCA	high grade *vs.* low grade	0.14	UP	EXP00401
hsa-miR-4443	TCGA_THCA	cancer *vs.* normal	0.13	UP	EXP00396
hsa-miR-1288-3p	TCGA_THCA	high grade *vs.* low grade	0.13	UP	EXP00398
hsa-miR-152-5p	TCGA_THCA	high grade *vs.* low grade	0.13	UP	EXP00398
hsa-miR-5187-5p	TCGA_THCA	high grade *vs.* low grade	0.13	UP	EXP00398
hsa-miR-154-3p	TCGA_THCA	high grade *vs.* low grade	0.13	UP	EXP00400
hsa-miR-450a-1-3p	TCGA_THCA	cancer *vs.* normal	0.12	UP	EXP00396
hsa-miR-4742-3p	TCGA_THCA	cancer *vs.* normal	0.12	UP	EXP00396
hsa-miR-2277-5p	TCGA_THCA	cancer *vs.* normal	0.12	UP	EXP00396
hsa-miR-6808-3p	TCGA_THCA	cancer *vs.* normal	0.12	UP	EXP00396
hsa-miR-676-5p	TCGA_THCA	high grade *vs.* low grade	0.12	UP	EXP00398
hsa-miR-511-3p	TCGA_THCA	high grade *vs.* low grade	0.12	UP	EXP00401
hsa-miR-6730-3p	TCGA_THCA	cancer *vs.* normal	0.11	UP	EXP00396
hsa-miR-6874-3p	TCGA_THCA	cancer *vs.* normal	0.11	UP	EXP00396
hsa-miR-511-3p	TCGA_THCA	high grade *vs.* low grade	0.11	UP	EXP00402
hsa-miR-1228-3p	TCGA_THCA	cancer *vs.* normal	0.1	UP	EXP00396

hsa-miR-4797-3p	TCGA_THCA	cancer *vs.* normal	0.1	UP	EXP00396
hsa-miR-664b-5p	TCGA_THCA	cancer *vs.* normal	0.1	UP	EXP00396
hsa-miR-212-5p	TCGA_THCA	cancer *vs.* normal	0.1	UP	EXP00396
hsa-miR-548v	TCGA_THCA	cancer *vs.* normal	0.1	UP	EXP00396
hsa-miR-6716-3p	TCGA_THCA	cancer *vs.* normal	0.1	UP	EXP00396
hsa-miR-664b-5p	TCGA_THCA	high grade *vs.* low grade	0.1	UP	EXP00398
hsa-miR-154-3p	TCGA_THCA	high grade *vs.* low grade	0.1	UP	EXP00399
hsa-miR-183-3p	TCGA_THCA	cancer *vs.* normal	0.09	UP	EXP00396
hsa-miR-3922-3p	TCGA_THCA	cancer *vs.* normal	0.09	UP	EXP00396
hsa-miR-6514-5p	TCGA_THCA	cancer *vs.* normal	0.09	UP	EXP00396
hsa-miR-632	TCGA_THCA	cancer *vs.* normal	0.09	UP	EXP00396
hsa-miR-5684	TCGA_THCA	cancer *vs.* normal	0.09	UP	EXP00396
hsa-miR-1229-3p	TCGA_THCA	cancer *vs.* normal	0.09	UP	EXP00396
hsa-miR-6875-5p	TCGA_THCA	cancer *vs.* normal	0.09	UP	EXP00396
hsa-miR-6798-3p	TCGA_THCA	cancer *vs.* normal	0.09	UP	EXP00396
hsa-miR-618	TCGA_THCA	high grade *vs.* low grade	0.09	UP	EXP00398
hsa-miR-3610	TCGA_THCA	cancer *vs.* normal	0.08	UP	EXP00396
hsa-miR-1254	TCGA_THCA	cancer *vs.* normal	0.08	UP	EXP00396
hsa-miR-2116-3p	TCGA_THCA	cancer *vs.* normal	0.08	UP	EXP00396
hsa-miR-6514-3p	TCGA_THCA	cancer *vs.* normal	0.08	UP	EXP00396
hsa-miR-4423-5p	TCGA_THCA	cancer *vs.* normal	0.08	UP	EXP00396
hsa-miR-765	TCGA_THCA	cancer *vs.* normal	0.08	UP	EXP00396
hsa-miR-3690	TCGA_THCA	cancer *vs.* normal	0.08	UP	EXP00396
hsa-miR-449a	TCGA_THCA	cancer *vs.* normal	0.08	UP	EXP00396
hsa-miR-1306-3p	TCGA_THCA	cancer *vs.* normal	0.08	UP	EXP00396
hsa-miR-4660	TCGA_THCA	cancer *vs.* normal	0.07	UP	EXP00396
hsa-miR-760	TCGA_THCA	high grade *vs.* low grade	0.07	UP	EXP00398
hsa-miR-18a-3p	TCGA_THCA	high grade *vs.* low grade	0.07	UP	EXP00401
hsa-miR-581	TCGA_THCA	cancer *vs.* normal	0.06	UP	EXP00396
hsa-miR-6734-5p	TCGA_THCA	cancer *vs.* normal	0.06	UP	EXP00396
hsa-miR-7705	TCGA_THCA	cancer *vs.* normal	0.06	UP	EXP00396
hsa-miR-497-3p	TCGA_THCA	cancer *vs.* normal	0.06	UP	EXP00396
hsa-miR-939-5p	TCGA_THCA	cancer *vs.* normal	0.05	UP	EXP00396
hsa-miR-3157-3p	TCGA_THCA	cancer *vs.* normal	-0.05	DOWN	EXP00396
hsa-miR-320d	TCGA_THCA	high grade *vs.* low grade	-0.06	DOWN	EXP00399
hsa-miR-3199	TCGA_THCA	cancer *vs.* normal	-0.07	DOWN	EXP00396
hsa-miR-3157-3p	TCGA_THCA	high grade *vs.* low grade	-0.07	DOWN	EXP00402
hsa-miR-548j-5p	TCGA_THCA	cancer *vs.* normal	-0.08	DOWN	EXP00396
hsa-miR-4683	TCGA_THCA	cancer *vs.* normal	-0.08	DOWN	EXP00396
hsa-miR-760	TCGA_THCA	high grade *vs.* low grade	-0.09	DOWN	EXP00401
hsa-miR-618	TCGA_THCA	high grade *vs.* low grade	-0.09	DOWN	EXP00401
hsa-miR-4791	TCGA_THCA	high grade *vs.* low grade	-0.09	DOWN	EXP00401
hsa-miR-3682-3p	TCGA_THCA	high grade *vs.* low grade	-0.09	DOWN	EXP00401
hsa-miR-34b-3p	TCGA_THCA	cancer *vs.* normal	-0.1	DOWN	EXP00396
hsa-miR-4638-3p	TCGA_THCA	high grade *vs.* low grade	-0.1	DOWN	EXP00401
hsa-miR-618	TCGA_THCA	high grade *vs.* low grade	-0.1	DOWN	EXP00402
hsa-miR-6761-5p	TCGA_THCA	high grade *vs.* low grade	-0.1	DOWN	EXP00402
hsa-miR-1291	TCGA_THCA	cancer *vs.* normal	-0.11	DOWN	EXP00396
hsa-miR-100-3p	TCGA_THCA	cancer *vs.* normal	-0.11	DOWN	EXP00396
hsa-miR-1249-3p	TCGA_THCA	cancer *vs.* normal	-0.11	DOWN	EXP00396
hsa-miR-5000-3p	TCGA_THCA	high grade *vs.* low grade	-0.11	DOWN	EXP00399

hsa-miR-3920	TCGA_THCA	high grade *vs.* low grade	-0.11	DOWN	EXP00401
hsa-miR-676-5p	TCGA_THCA	high grade *vs.* low grade	-0.12	DOWN	EXP00402
hsa-miR-6806-3p	TCGA_THCA	cancer *vs.* normal	-0.13	DOWN	EXP00396
hsa-miR-664b-5p	TCGA_THCA	high grade *vs.* low grade	-0.13	DOWN	EXP00402
hsa-miR-100-3p	TCGA_THCA	high grade *vs.* low grade	-0.13	DOWN	EXP00402
hsa-miR-4638-3p	TCGA_THCA	high grade *vs.* low grade	-0.13	DOWN	EXP00402
hsa-miR-624-5p	TCGA_THCA	cancer *vs.* normal	-0.14	DOWN	EXP00396
hsa-miR-628-3p	TCGA_THCA	cancer *vs.* normal	-0.14	DOWN	EXP00396
hsa-miR-1288-3p	TCGA_THCA	high grade *vs.* low grade	-0.14	DOWN	EXP00401
hsa-miR-191-3p	TCGA_THCA	high grade *vs.* low grade	-0.14	DOWN	EXP00401
hsa-miR-331-5p	TCGA_THCA	high grade *vs.* low grade	-0.14	DOWN	EXP00401
hsa-miR-3920	TCGA_THCA	high grade *vs.* low grade	-0.14	DOWN	EXP00402
hsa-miR-152-5p	TCGA_THCA	high grade *vs.* low grade	-0.14	DOWN	EXP00402
hsa-miR-550a-3p	TCGA_THCA	cancer *vs.* normal	-0.15	DOWN	EXP00396
hsa-miR-499a-5p	TCGA_THCA	cancer *vs.* normal	-0.15	DOWN	EXP00396
hsa-miR-369-5p	TCGA_THCA	cancer *vs.* normal	-0.15	DOWN	EXP00396
hsa-miR-7706	TCGA_THCA	high grade *vs.* low grade	-0.15	DOWN	EXP00402
hsa-miR-6820-3p	TCGA_THCA	cancer *vs.* normal	-0.16	DOWN	EXP00396
hsa-miR-502-3p	TCGA_THCA	cancer *vs.* normal	-0.16	DOWN	EXP00396
hsa-miR-192-3p	TCGA_THCA	cancer *vs.* normal	-0.16	DOWN	EXP00396
hsa-miR-676-5p	TCGA_THCA	high grade *vs.* low grade	-0.16	DOWN	EXP00401
hsa-miR-3193	TCGA_THCA	high grade *vs.* low grade	-0.16	DOWN	EXP00401
hsa-miR-151a-5p	TCGA_THCA	high grade *vs.* low grade	-0.16	DOWN	EXP00401
hsa-miR-548b-3p	TCGA_THCA	cancer *vs.* normal	-0.17	DOWN	EXP00396
hsa-miR-1976	TCGA_THCA	cancer *vs.* normal	-0.17	DOWN	EXP00396
hsa-miR-5010-3p	TCGA_THCA	high grade *vs.* low grade	-0.17	DOWN	EXP00398
hsa-miR-320b	TCGA_THCA	high grade *vs.* low grade	-0.17	DOWN	EXP00399
hsa-miR-152-5p	TCGA_THCA	high grade *vs.* low grade	-0.17	DOWN	EXP00401
hsa-miR-4772-3p	TCGA_THCA	cancer *vs.* normal	-0.18	DOWN	EXP00396
hsa-miR-502-3p	TCGA_THCA	high grade *vs.* low grade	-0.18	DOWN	EXP00401
hsa-miR-3193	TCGA_THCA	high grade *vs.* low grade	-0.18	DOWN	EXP00402
hsa-miR-3651	TCGA_THCA	cancer *vs.* normal	-0.19	DOWN	EXP00396
hsa-miR-320b	TCGA_THCA	cancer *vs.* normal	-0.19	DOWN	EXP00396
hsa-miR-197-3p	TCGA_THCA	cancer *vs.* normal	-0.19	DOWN	EXP00396
hsa-miR-320b	TCGA_THCA	high grade *vs.* low grade	-0.19	DOWN	EXP00401
hsa-miR-550a-5p	TCGA_THCA	high grade *vs.* low grade	-0.19	DOWN	EXP00402
hsa-miR-6806-3p	TCGA_THCA	high grade *vs.* low grade	-0.19	DOWN	EXP00402
hsa-miR-153-3p	TCGA_THCA	cancer *vs.* normal	-0.2	DOWN	EXP00396
hsa-miR-550a-5p	TCGA_THCA	high grade *vs.* low grade	-0.2	DOWN	EXP00401
hsa-miR-361-5p	TCGA_THCA	high grade *vs.* low grade	-0.2	DOWN	EXP00401
hsa-miR-1287-5p	TCGA_THCA	cancer *vs.* normal	-0.21	DOWN	EXP00396
hsa-miR-1301-3p	TCGA_THCA	high grade *vs.* low grade	-0.21	DOWN	EXP00399
hsa-miR-188-3p	TCGA_THCA	high grade *vs.* low grade	-0.21	DOWN	EXP00401
hsa-miR-195-3p	TCGA_THCA	high grade *vs.* low grade	-0.21	DOWN	EXP00401
hsa-miR-3605-3p	TCGA_THCA	high grade *vs.* low grade	-0.21	DOWN	EXP00401
hsa-miR-331-5p	TCGA_THCA	high grade *vs.* low grade	-0.21	DOWN	EXP00402
hsa-miR-486-3p	TCGA_THCA	cancer *vs.* normal	-0.22	DOWN	EXP00396
hsa-miR-223-5p	TCGA_THCA	cancer *vs.* normal	-0.22	DOWN	EXP00396
hsa-miR-92a-1-5p	TCGA_THCA	cancer *vs.* normal	-0.22	DOWN	EXP00396
hsa-miR-335-5p	TCGA_THCA	cancer *vs.* normal	-0.22	DOWN	EXP00396
hsa-miR-1288-3p	TCGA_THCA	high grade *vs.* low grade	-0.22	DOWN	EXP00402

hsa-miR-3677-5p	TCGA_THCA	high grade *vs.* low grade	-0.22	DOWN	EXP00402
hsa-miR-106b-5p	TCGA_THCA	cancer *vs.* normal	-0.23	DOWN	EXP00396
hsa-miR-944	TCGA_THCA	high grade *vs.* low grade	-0.23	DOWN	EXP00398
hsa-miR-3677-5p	TCGA_THCA	high grade *vs.* low grade	-0.23	DOWN	EXP00401
hsa-miR-30d-3p	TCGA_THCA	high grade *vs.* low grade	-0.23	DOWN	EXP00401
hsa-miR-24-3p	TCGA_THCA	cancer *vs.* normal	-0.24	DOWN	EXP00396
hsa-miR-17-3p	TCGA_THCA	cancer *vs.* normal	-0.24	DOWN	EXP00396
hsa-miR-145-3p	TCGA_THCA	cancer *vs.* normal	-0.24	DOWN	EXP00396
hsa-miR-17-3p	TCGA_THCA	high grade *vs.* low grade	-0.24	DOWN	EXP00398
hsa-miR-154-5p	TCGA_THCA	high grade *vs.* low grade	-0.24	DOWN	EXP00398
hsa-miR-26b-3p	TCGA_THCA	high grade *vs.* low grade	-0.24	DOWN	EXP00398
hsa-miR-363-3p	TCGA_THCA	high grade *vs.* low grade	-0.24	DOWN	EXP00399
hsa-miR-6806-3p	TCGA_THCA	high grade *vs.* low grade	-0.24	DOWN	EXP00401
hsa-miR-421	TCGA_THCA	high grade *vs.* low grade	-0.24	DOWN	EXP00401
hsa-miR-1306-5p	TCGA_THCA	high grade *vs.* low grade	-0.24	DOWN	EXP00401
hsa-miR-30d-3p	TCGA_THCA	high grade *vs.* low grade	-0.24	DOWN	EXP00402
hsa-miR-5000-3p	TCGA_THCA	cancer *vs.* normal	-0.25	DOWN	EXP00396
hsa-miR-195-3p	TCGA_THCA	cancer *vs.* normal	-0.25	DOWN	EXP00396
hsa-miR-374a-5p	TCGA_THCA	cancer *vs.* normal	-0.25	DOWN	EXP00396
hsa-miR-376c-3p	TCGA_THCA	cancer *vs.* normal	-0.25	DOWN	EXP00396
hsa-miR-574-3p	TCGA_THCA	high grade *vs.* low grade	-0.25	DOWN	EXP00398
hsa-miR-3130-5p	TCGA_THCA	high grade *vs.* low grade	-0.25	DOWN	EXP00399
hsa-miR-148b-3p	TCGA_THCA	high grade *vs.* low grade	-0.25	DOWN	EXP00400
hsa-miR-320a	TCGA_THCA	high grade *vs.* low grade	-0.25	DOWN	EXP00401
hsa-miR-769-5p	TCGA_THCA	high grade *vs.* low grade	-0.25	DOWN	EXP00402
hsa-miR-140-5p	TCGA_THCA	cancer *vs.* normal	-0.26	DOWN	EXP00396
hsa-miR-320a	TCGA_THCA	cancer *vs.* normal	-0.26	DOWN	EXP00396
hsa-miR-106b-3p	TCGA_THCA	cancer *vs.* normal	-0.26	DOWN	EXP00396
hsa-miR-339-3p	TCGA_THCA	high grade *vs.* low grade	-0.26	DOWN	EXP00401
hsa-miR-1307-3p	TCGA_THCA	high grade *vs.* low grade	-0.26	DOWN	EXP00401
hsa-miR-361-5p	TCGA_THCA	high grade *vs.* low grade	-0.26	DOWN	EXP00402
hsa-miR-23a-3p	TCGA_THCA	cancer *vs.* normal	-0.27	DOWN	EXP00396
hsa-miR-4772-3p	TCGA_THCA	high grade *vs.* low grade	-0.27	DOWN	EXP00398
hsa-let-7b-5p	TCGA_THCA	high grade *vs.* low grade	-0.27	DOWN	EXP00399
hsa-miR-589-5p	TCGA_THCA	high grade *vs.* low grade	-0.27	DOWN	EXP00401
hsa-miR-532-3p	TCGA_THCA	high grade *vs.* low grade	-0.27	DOWN	EXP00401
hsa-miR-188-3p	TCGA_THCA	high grade *vs.* low grade	-0.27	DOWN	EXP00402
hsa-let-7d-5p	TCGA_THCA	high grade *vs.* low grade	-0.27	DOWN	EXP00402
hsa-miR-3605-3p	TCGA_THCA	high grade *vs.* low grade	-0.27	DOWN	EXP00402
hsa-miR-3615	TCGA_THCA	cancer *vs.* normal	-0.28	DOWN	EXP00396
hsa-miR-1275	TCGA_THCA	cancer *vs.* normal	-0.28	DOWN	EXP00396
hsa-miR-1301-3p	TCGA_THCA	high grade *vs.* low grade	-0.28	DOWN	EXP00402
hsa-let-7d-3p	TCGA_THCA	high grade *vs.* low grade	-0.28	DOWN	EXP00402
hsa-miR-874-5p	TCGA_THCA	high grade *vs.* low grade	-0.28	DOWN	EXP00402
hsa-miR-30b-5p	TCGA_THCA	cancer *vs.* normal	-0.29	DOWN	EXP00396
hsa-miR-192-5p	TCGA_THCA	cancer *vs.* normal	-0.29	DOWN	EXP00396
hsa-miR-128-1-5p	TCGA_THCA	high grade *vs.* low grade	-0.29	DOWN	EXP00401
hsa-miR-628-5p	TCGA_THCA	high grade *vs.* low grade	-0.29	DOWN	EXP00401
hsa-miR-744-3p	TCGA_THCA	high grade *vs.* low grade	-0.29	DOWN	EXP00401
hsa-miR-378c	TCGA_THCA	high grade *vs.* low grade	-0.29	DOWN	EXP00401
hsa-miR-192-5p	TCGA_THCA	high grade *vs.* low grade	-0.29	DOWN	EXP00401

hsa-miR-195-3p	TCGA_THCA	high grade *vs.* low grade	-0.29	DOWN	EXP00402
hsa-miR-339-3p	TCGA_THCA	high grade *vs.* low grade	-0.29	DOWN	EXP00402
hsa-miR-185-5p	TCGA_THCA	high grade *vs.* low grade	-0.29	DOWN	EXP00402
hsa-miR-194-3p	TCGA_THCA	cancer *vs.* normal	-0.3	DOWN	EXP00396
hsa-miR-193b-5p	TCGA_THCA	cancer *vs.* normal	-0.3	DOWN	EXP00396
hsa-miR-1270	TCGA_THCA	cancer *vs.* normal	-0.3	DOWN	EXP00396
hsa-miR-28-3p	TCGA_THCA	high grade *vs.* low grade	-0.3	DOWN	EXP00400
hsa-miR-185-5p	TCGA_THCA	high grade *vs.* low grade	-0.3	DOWN	EXP00401
hsa-miR-676-3p	TCGA_THCA	high grade *vs.* low grade	-0.3	DOWN	EXP00402
hsa-miR-532-5p	TCGA_THCA	high grade *vs.* low grade	-0.3	DOWN	EXP00402
hsa-miR-374b-5p	TCGA_THCA	high grade *vs.* low grade	-0.3	DOWN	EXP00402
hsa-miR-16-5p	TCGA_THCA	cancer *vs.* normal	-0.31	DOWN	EXP00396
hsa-miR-4662a-5p	TCGA_THCA	cancer *vs.* normal	-0.31	DOWN	EXP00396
hsa-miR-5586-5p	TCGA_THCA	high grade *vs.* low grade	-0.31	DOWN	EXP00398
hsa-miR-342-3p	TCGA_THCA	high grade *vs.* low grade	-0.31	DOWN	EXP00398
hsa-miR-652-3p	TCGA_THCA	high grade *vs.* low grade	-0.31	DOWN	EXP00399
hsa-miR-1301-3p	TCGA_THCA	high grade *vs.* low grade	-0.31	DOWN	EXP00401
hsa-miR-585-3p	TCGA_THCA	high grade *vs.* low grade	-0.31	DOWN	EXP00401
hsa-miR-324-3p	TCGA_THCA	high grade *vs.* low grade	-0.31	DOWN	EXP00401
hsa-miR-5091	TCGA_THCA	high grade *vs.* low grade	-0.31	DOWN	EXP00401
hsa-miR-28-5p	TCGA_THCA	high grade *vs.* low grade	-0.31	DOWN	EXP00402
hsa-miR-5091	TCGA_THCA	high grade *vs.* low grade	-0.31	DOWN	EXP00402
hsa-miR-26a	GSE40807	cancer *vs.* normal	-0.32	DOWN	EXP00274
hsa-miR-582-3p	TCGA_THCA	cancer *vs.* normal	-0.32	DOWN	EXP00396
hsa-miR-126-3p	TCGA_THCA	high grade *vs.* low grade	-0.32	DOWN	EXP00399
hsa-miR-28-5p	TCGA_THCA	high grade *vs.* low grade	-0.32	DOWN	EXP00401
hsa-miR-194-3p	TCGA_THCA	high grade *vs.* low grade	-0.32	DOWN	EXP00401
hsa-miR-625-3p	TCGA_THCA	high grade *vs.* low grade	-0.33	DOWN	EXP00398
hsa-miR-3074-5p	TCGA_THCA	high grade *vs.* low grade	-0.33	DOWN	EXP00400
hsa-miR-676-3p	TCGA_THCA	high grade *vs.* low grade	-0.33	DOWN	EXP00401
hsa-miR-30e-5p	TCGA_THCA	high grade *vs.* low grade	-0.33	DOWN	EXP00401
hsa-miR-3677-3p	TCGA_THCA	high grade *vs.* low grade	-0.33	DOWN	EXP00402
hsa-miR-651-5p	TCGA_THCA	high grade *vs.* low grade	-0.33	DOWN	EXP00402
hsa-miR-15b-3p	TCGA_THCA	cancer *vs.* normal	-0.34	DOWN	EXP00396
hsa-let-7g-5p	TCGA_THCA	cancer *vs.* normal	-0.34	DOWN	EXP00396
hsa-miR-19b-3p	TCGA_THCA	high grade *vs.* low grade	-0.34	DOWN	EXP00398
hsa-miR-212-3p	TCGA_THCA	high grade *vs.* low grade	-0.34	DOWN	EXP00399
hsa-miR-1251-3p	TCGA_THCA	high grade *vs.* low grade	-0.34	DOWN	EXP00401
hsa-miR-3130-5p	TCGA_THCA	high grade *vs.* low grade	-0.34	DOWN	EXP00401
hsa-miR-194-3p	TCGA_THCA	high grade *vs.* low grade	-0.34	DOWN	EXP00402
hsa-miR-664a-5p	TCGA_THCA	high grade *vs.* low grade	-0.34	DOWN	EXP00402
hsa-miR-509-3-5p	GSE40807	cancer *vs.* normal	-0.35	DOWN	EXP00274
hsa-miR-3653-3p	TCGA_THCA	cancer *vs.* normal	-0.35	DOWN	EXP00396
hsa-let-7a-2-3p	TCGA_THCA	cancer *vs.* normal	-0.35	DOWN	EXP00396
hsa-miR-19b-1-5p	TCGA_THCA	high grade *vs.* low grade	-0.35	DOWN	EXP00398
hsa-miR-1247-5p	TCGA_THCA	high grade *vs.* low grade	-0.35	DOWN	EXP00398
hsa-miR-126-5p	TCGA_THCA	high grade *vs.* low grade	-0.35	DOWN	EXP00399
hsa-miR-139-5p	TCGA_THCA	high grade *vs.* low grade	-0.35	DOWN	EXP00399
hsa-miR-30c-1-3p	TCGA_THCA	high grade *vs.* low grade	-0.35	DOWN	EXP00401
hsa-miR-940	TCGA_THCA	high grade *vs.* low grade	-0.35	DOWN	EXP00401
hsa-miR-30b-3p	TCGA_THCA	high grade *vs.* low grade	-0.35	DOWN	EXP00401

hsa-miR-140-3p	TCGA_THCA	high grade *vs.* low grade	-0.35	DOWN	EXP00401
hsa-miR-940	TCGA_THCA	high grade *vs.* low grade	-0.35	DOWN	EXP00402
hsa-miR-585-3p	TCGA_THCA	high grade *vs.* low grade	-0.35	DOWN	EXP00402
hsa-miR-128-3p	TCGA_THCA	high grade *vs.* low grade	-0.35	DOWN	EXP00402
hsa-miR-320a	TCGA_THCA	high grade *vs.* low grade	-0.35	DOWN	EXP00402
hsa-miR-378c	TCGA_THCA	high grade *vs.* low grade	-0.35	DOWN	EXP00402
hsa-miR-3200-3p	TCGA_THCA	high grade *vs.* low grade	-0.35	DOWN	EXP00402
hsa-miR-574-3p	TCGA_THCA	cancer *vs.* normal	-0.36	DOWN	EXP00396
hsa-miR-22-3p	TCGA_THCA	cancer *vs.* normal	-0.36	DOWN	EXP00396
hsa-miR-758-5p	TCGA_THCA	cancer *vs.* normal	-0.36	DOWN	EXP00396
hsa-miR-425-5p	TCGA_THCA	cancer *vs.* normal	-0.36	DOWN	EXP00396
hsa-miR-30c-5p	TCGA_THCA	cancer *vs.* normal	-0.36	DOWN	EXP00396
hsa-miR-219a-1-3p	TCGA_THCA	high grade *vs.* low grade	-0.36	DOWN	EXP00401
hsa-miR-1287-5p	TCGA_THCA	high grade *vs.* low grade	-0.36	DOWN	EXP00401
hsa-miR-138-1-3p	TCGA_THCA	high grade *vs.* low grade	-0.36	DOWN	EXP00401
hsa-miR-338-5p	TCGA_THCA	high grade *vs.* low grade	-0.36	DOWN	EXP00401
hsa-miR-22-3p	TCGA_THCA	high grade *vs.* low grade	-0.36	DOWN	EXP00402
hsa-miR-1976	TCGA_THCA	high grade *vs.* low grade	-0.36	DOWN	EXP00402
hsa-miR-598-3p	TCGA_THCA	high grade *vs.* low grade	-0.36	DOWN	EXP00402
hsa-miR-328-3p	TCGA_THCA	high grade *vs.* low grade	-0.36	DOWN	EXP00402
hsa-miR-128-1-5p	TCGA_THCA	cancer *vs.* normal	-0.37	DOWN	EXP00396
hsa-let-7b-3p	TCGA_THCA	cancer *vs.* normal	-0.37	DOWN	EXP00396
hsa-miR-409-5p	TCGA_THCA	cancer *vs.* normal	-0.37	DOWN	EXP00396
hsa-miR-19a-3p	TCGA_THCA	high grade *vs.* low grade	-0.37	DOWN	EXP00398
hsa-miR-200c-5p	TCGA_THCA	high grade *vs.* low grade	-0.37	DOWN	EXP00401
hsa-miR-598-3p	TCGA_THCA	high grade *vs.* low grade	-0.37	DOWN	EXP00401
hsa-miR-744-5p	TCGA_THCA	high grade *vs.* low grade	-0.37	DOWN	EXP00401
hsa-miR-30c-5p	TCGA_THCA	high grade *vs.* low grade	-0.37	DOWN	EXP00401
hsa-miR-96-5p	TCGA_THCA	high grade *vs.* low grade	-0.37	DOWN	EXP00401
hsa-miR-128-1-5p	TCGA_THCA	high grade *vs.* low grade	-0.37	DOWN	EXP00402
hsa-miR-935	TCGA_THCA	high grade *vs.* low grade	-0.37	DOWN	EXP00402
hsa-miR-30b-3p	TCGA_THCA	high grade *vs.* low grade	-0.37	DOWN	EXP00402
hsa-miR-128-3p	TCGA_THCA	high grade *vs.* low grade	-0.38	DOWN	EXP00401
hsa-miR-22-3p	TCGA_THCA	high grade *vs.* low grade	-0.38	DOWN	EXP00401
hsa-miR-3912-3p	TCGA_THCA	high grade *vs.* low grade	-0.38	DOWN	EXP00401
hsa-miR-7-2-3p	TCGA_THCA	high grade *vs.* low grade	-0.38	DOWN	EXP00401
hsa-miR-151a-3p	TCGA_THCA	high grade *vs.* low grade	-0.38	DOWN	EXP00402
hsa-miR-339-5p	GSE40807	cancer *vs.* normal	-0.39	DOWN	EXP00274
hsa-miR-140-3p	TCGA_THCA	cancer *vs.* normal	-0.39	DOWN	EXP00396
hsa-miR-30a-3p	TCGA_THCA	high grade *vs.* low grade	-0.39	DOWN	EXP00400
hsa-miR-1275	TCGA_THCA	high grade *vs.* low grade	-0.39	DOWN	EXP00401
hsa-miR-3677-3p	TCGA_THCA	high grade *vs.* low grade	-0.39	DOWN	EXP00401
hsa-miR-671-3p	TCGA_THCA	high grade *vs.* low grade	-0.39	DOWN	EXP00402
hsa-miR-140-3p	TCGA_THCA	high grade *vs.* low grade	-0.39	DOWN	EXP00402
hsa-miR-98-5p	TCGA_THCA	high grade *vs.* low grade	-0.39	DOWN	EXP00402
hsa-miR-95-3p	TCGA_THCA	high grade *vs.* low grade	-0.39	DOWN	EXP00402
hsa-miR-138-1-3p	TCGA_THCA	high grade *vs.* low grade	-0.39	DOWN	EXP00402
hsa-miR-93	GSE40807	cancer *vs.* normal	-0.4	DOWN	EXP00274
hsa-miR-497	GSE40807	cancer *vs.* normal	-0.4	DOWN	EXP00274
hsa-miR-133b	TCGA_THCA	cancer *vs.* normal	-0.4	DOWN	EXP00396
hsa-miR-3130-5p	TCGA_THCA	cancer *vs.* normal	-0.4	DOWN	EXP00396

hsa-let-7i-3p	TCGA_THCA	cancer *vs.* normal	-0.4	DOWN	EXP00396
hsa-miR-338-5p	TCGA_THCA	high grade *vs.* low grade	-0.4	DOWN	EXP00400
hsa-miR-664a-5p	TCGA_THCA	high grade *vs.* low grade	-0.4	DOWN	EXP00401
hsa-miR-328-3p	TCGA_THCA	high grade *vs.* low grade	-0.4	DOWN	EXP00401
hsa-miR-30c-1-3p	TCGA_THCA	high grade *vs.* low grade	-0.4	DOWN	EXP00402
hsa-miR-30e-5p	TCGA_THCA	high grade *vs.* low grade	-0.4	DOWN	EXP00402
hsa-miR-411-5p	TCGA_THCA	cancer *vs.* normal	-0.41	DOWN	EXP00396
hsa-miR-532-5p	TCGA_THCA	cancer *vs.* normal	-0.41	DOWN	EXP00396
hsa-miR-326	TCGA_THCA	cancer *vs.* normal	-0.41	DOWN	EXP00396
hsa-miR-874-3p	TCGA_THCA	cancer *vs.* normal	-0.41	DOWN	EXP00396
hsa-miR-30c-2-3p	TCGA_THCA	high grade *vs.* low grade	-0.41	DOWN	EXP00400
hsa-miR-935	TCGA_THCA	high grade *vs.* low grade	-0.41	DOWN	EXP00401
hsa-miR-185-3p	TCGA_THCA	high grade *vs.* low grade	-0.41	DOWN	EXP00401
hsa-miR-450a-5p	TCGA_THCA	high grade *vs.* low grade	-0.41	DOWN	EXP00401
hsa-miR-628-5p	TCGA_THCA	high grade *vs.* low grade	-0.41	DOWN	EXP00402
hsa-miR-23b-3p	TCGA_THCA	high grade *vs.* low grade	-0.41	DOWN	EXP00402
hsa-miR-200c-5p	TCGA_THCA	high grade *vs.* low grade	-0.41	DOWN	EXP00402
hsa-miR-331-3p	TCGA_THCA	high grade *vs.* low grade	-0.41	DOWN	EXP00402
hsa-miR-106a-5p	TCGA_THCA	cancer *vs.* normal	-0.42	DOWN	EXP00396
hsa-miR-154-5p	TCGA_THCA	cancer *vs.* normal	-0.42	DOWN	EXP00396
hsa-let-7i-5p	TCGA_THCA	cancer *vs.* normal	-0.42	DOWN	EXP00396
hsa-let-7c-5p	TCGA_THCA	cancer *vs.* normal	-0.42	DOWN	EXP00396
hsa-miR-130a-3p	TCGA_THCA	cancer *vs.* normal	-0.42	DOWN	EXP00396
hsa-miR-23b-3p	TCGA_THCA	high grade *vs.* low grade	-0.42	DOWN	EXP00401
hsa-miR-98-5p	TCGA_THCA	high grade *vs.* low grade	-0.42	DOWN	EXP00401
hsa-miR-92b*	GSE40807	cancer *vs.* normal	-0.43	DOWN	EXP00274
hsa-miR-186-5p	TCGA_THCA	cancer *vs.* normal	-0.43	DOWN	EXP00396
hsa-miR-361-3p	TCGA_THCA	cancer *vs.* normal	-0.43	DOWN	EXP00396
hsa-miR-455-5p	TCGA_THCA	high grade *vs.* low grade	-0.43	DOWN	EXP00398
hsa-miR-652-3p	TCGA_THCA	high grade *vs.* low grade	-0.43	DOWN	EXP00400
hsa-miR-671-3p	TCGA_THCA	high grade *vs.* low grade	-0.43	DOWN	EXP00401
hsa-miR-424-3p	TCGA_THCA	high grade *vs.* low grade	-0.43	DOWN	EXP00401
hsa-miR-1275	TCGA_THCA	high grade *vs.* low grade	-0.43	DOWN	EXP00402
hsa-miR-26b-3p	TCGA_THCA	cancer *vs.* normal	-0.44	DOWN	EXP00396
hsa-miR-30e-3p	TCGA_THCA	cancer *vs.* normal	-0.44	DOWN	EXP00396
hsa-miR-584-5p	TCGA_THCA	cancer *vs.* normal	-0.44	DOWN	EXP00396
hsa-miR-130b-3p	TCGA_THCA	cancer *vs.* normal	-0.44	DOWN	EXP00396
hsa-miR-136-3p	TCGA_THCA	cancer *vs.* normal	-0.44	DOWN	EXP00396
hsa-miR-210-3p	TCGA_THCA	cancer *vs.* normal	-0.44	DOWN	EXP00396
hsa-miR-139-3p	TCGA_THCA	high grade *vs.* low grade	-0.44	DOWN	EXP00399
hsa-miR-4662a-5p	TCGA_THCA	high grade *vs.* low grade	-0.44	DOWN	EXP00401
hsa-miR-182-5p	TCGA_THCA	high grade *vs.* low grade	-0.44	DOWN	EXP00401
hsa-miR-30e	GSE40807	cancer *vs.* normal	-0.45	DOWN	EXP00274
hsa-miR-193a-3p	TCGA_THCA	cancer *vs.* normal	-0.45	DOWN	EXP00396
hsa-miR-3607-3p	TCGA_THCA	cancer *vs.* normal	-0.45	DOWN	EXP00396
hsa-miR-363-3p	TCGA_THCA	high grade *vs.* low grade	-0.45	DOWN	EXP00401
hsa-miR-584-5p	TCGA_THCA	high grade *vs.* low grade	-0.45	DOWN	EXP00401
hsa-miR-30a-3p	TCGA_THCA	high grade *vs.* low grade	-0.45	DOWN	EXP00401
hsa-miR-145-3p	TCGA_THCA	high grade *vs.* low grade	-0.45	DOWN	EXP00401
hsa-miR-219a-1-3p	TCGA_THCA	high grade *vs.* low grade	-0.45	DOWN	EXP00402
hsa-miR-1287-5p	TCGA_THCA	high grade *vs.* low grade	-0.45	DOWN	EXP00402

hsa-miR-25-3p	TCGA_THCA	cancer *vs.* normal	-0.46	DOWN	EXP00396
hsa-miR-136-5p	TCGA_THCA	high grade *vs.* low grade	-0.46	DOWN	EXP00398
hsa-miR-30e-3p	TCGA_THCA	high grade *vs.* low grade	-0.46	DOWN	EXP00401
hsa-miR-3200-3p	TCGA_THCA	high grade *vs.* low grade	-0.46	DOWN	EXP00401
hsa-miR-452-5p	TCGA_THCA	high grade *vs.* low grade	-0.46	DOWN	EXP00401
hsa-miR-148a-3p	TCGA_THCA	high grade *vs.* low grade	-0.46	DOWN	EXP00402
hsa-miR-10b-5p	TCGA_THCA	cancer *vs.* normal	-0.47	DOWN	EXP00396
hsa-miR-126-3p	TCGA_THCA	high grade *vs.* low grade	-0.47	DOWN	EXP00400
hsa-miR-338-3p	TCGA_THCA	high grade *vs.* low grade	-0.47	DOWN	EXP00400
hsa-miR-28-3p	TCGA_THCA	high grade *vs.* low grade	-0.47	DOWN	EXP00402
hsa-let-7b	GSE40807	cancer *vs.* normal	-0.48	DOWN	EXP00274
hsa-miR-18a-5p	TCGA_THCA	cancer *vs.* normal	-0.48	DOWN	EXP00396
hsa-miR-3912-3p	TCGA_THCA	high grade *vs.* low grade	-0.48	DOWN	EXP00402
hsa-miR-363-3p	TCGA_THCA	high grade *vs.* low grade	-0.48	DOWN	EXP00402
hsa-miR-3912-3p	TCGA_THCA	cancer *vs.* normal	-0.49	DOWN	EXP00396
hsa-miR-26b-5p	TCGA_THCA	cancer *vs.* normal	-0.49	DOWN	EXP00396
hsa-miR-365a-3p	TCGA_THCA	cancer *vs.* normal	-0.49	DOWN	EXP00396
hsa-miR-365b-3p	TCGA_THCA	cancer *vs.* normal	-0.49	DOWN	EXP00396
hsa-miR-29c-3p	TCGA_THCA	cancer *vs.* normal	-0.49	DOWN	EXP00396
hsa-miR-148a-3p	TCGA_THCA	cancer *vs.* normal	-0.49	DOWN	EXP00396
hsa-miR-130b-5p	TCGA_THCA	cancer *vs.* normal	-0.49	DOWN	EXP00396
hsa-miR-223-3p	TCGA_THCA	high grade *vs.* low grade	-0.49	DOWN	EXP00398
hsa-miR-9-5p	TCGA_THCA	high grade *vs.* low grade	-0.49	DOWN	EXP00398
hsa-miR-151a-3p	TCGA_THCA	high grade *vs.* low grade	-0.49	DOWN	EXP00401
hsa-miR-30e-3p	TCGA_THCA	high grade *vs.* low grade	-0.49	DOWN	EXP00402
hsa-miR-148b-3p	TCGA_THCA	high grade *vs.* low grade	-0.5	DOWN	EXP00401
hsa-miR-190a-5p	TCGA_THCA	high grade *vs.* low grade	-0.5	DOWN	EXP00401
hsa-miR-20b-5p	TCGA_THCA	high grade *vs.* low grade	-0.5	DOWN	EXP00402
hsa-miR-942-5p	TCGA_THCA	cancer *vs.* normal	-0.51	DOWN	EXP00396
hsa-miR-126-5p	TCGA_THCA	high grade *vs.* low grade	-0.51	DOWN	EXP00400
hsa-miR-1180-3p	TCGA_THCA	high grade *vs.* low grade	-0.51	DOWN	EXP00401
hsa-miR-130b-5p	TCGA_THCA	high grade *vs.* low grade	-0.51	DOWN	EXP00402
hsa-miR-15b*	GSE40807	cancer *vs.* normal	-0.52	DOWN	EXP00274
hsa-miR-16-2-3p	TCGA_THCA	cancer *vs.* normal	-0.52	DOWN	EXP00396
hsa-miR-145-3p	TCGA_THCA	high grade *vs.* low grade	-0.52	DOWN	EXP00402
hsa-miR-338-5p	TCGA_THCA	high grade *vs.* low grade	-0.52	DOWN	EXP00402
hsa-miR-338-3p	TCGA_THCA	high grade *vs.* low grade	-0.52	DOWN	EXP00402
hsa-miR-196b-5p	TCGA_THCA	cancer *vs.* normal	-0.53	DOWN	EXP00396
hsa-miR-136-5p	TCGA_THCA	cancer *vs.* normal	-0.53	DOWN	EXP00396
hsa-miR-139-3p	TCGA_THCA	high grade *vs.* low grade	-0.53	DOWN	EXP00400
hsa-miR-139-5p	TCGA_THCA	high grade *vs.* low grade	-0.53	DOWN	EXP00400
hsa-miR-652-3p	TCGA_THCA	high grade *vs.* low grade	-0.53	DOWN	EXP00401
hsa-miR-4662a-5p	TCGA_THCA	high grade *vs.* low grade	-0.53	DOWN	EXP00402
hsa-miR-30c-5p	TCGA_THCA	high grade *vs.* low grade	-0.53	DOWN	EXP00402
hsa-miR-4732-3p	TCGA_THCA	cancer *vs.* normal	-0.54	DOWN	EXP00396
hsa-miR-214-5p	TCGA_THCA	high grade *vs.* low grade	-0.54	DOWN	EXP00398
hsa-miR-331-3p	TCGA_THCA	high grade *vs.* low grade	-0.54	DOWN	EXP00401
hsa-miR-7-2-3p	TCGA_THCA	high grade *vs.* low grade	-0.54	DOWN	EXP00402
hsa-miR-627	GSE40807	cancer *vs.* normal	-0.55	DOWN	EXP00274
hsa-miR-148b-3p	TCGA_THCA	cancer *vs.* normal	-0.55	DOWN	EXP00396
hsa-miR-199a-3p	TCGA_THCA	high grade *vs.* low grade	-0.55	DOWN	EXP00398

hsa-miR-148a-5p	TCGA_THCA	high grade *vs.* low grade	-0.55	DOWN	EXP00400
hsa-miR-3074-5p	TCGA_THCA	high grade *vs.* low grade	-0.55	DOWN	EXP00401
hsa-miR-145-5p	TCGA_THCA	high grade *vs.* low grade	-0.55	DOWN	EXP00402
hsa-miR-150-3p	TCGA_THCA	cancer *vs.* normal	-0.56	DOWN	EXP00396
hsa-miR-133a-3p	TCGA_THCA	cancer *vs.* normal	-0.56	DOWN	EXP00396
hsa-miR-584-5p	TCGA_THCA	high grade *vs.* low grade	-0.56	DOWN	EXP00402
hsa-miR-654-3p	TCGA_THCA	cancer *vs.* normal	-0.57	DOWN	EXP00396
hsa-miR-199b-3p	TCGA_THCA	high grade *vs.* low grade	-0.57	DOWN	EXP00398
hsa-miR-542-5p	TCGA_THCA	high grade *vs.* low grade	-0.57	DOWN	EXP00401
hsa-miR-148b-3p	TCGA_THCA	high grade *vs.* low grade	-0.57	DOWN	EXP00402
hsa-miR-509-5p	GSE40807	cancer *vs.* normal	-0.58	DOWN	EXP00274
hsa-miR-28-5p	TCGA_THCA	cancer *vs.* normal	-0.58	DOWN	EXP00396
hsa-miR-215-5p	TCGA_THCA	cancer *vs.* normal	-0.58	DOWN	EXP00396
hsa-miR-126-3p	TCGA_THCA	cancer *vs.* normal	-0.58	DOWN	EXP00396
hsa-miR-542-5p	TCGA_THCA	high grade *vs.* low grade	-0.58	DOWN	EXP00402
hsa-miR-143-3p	TCGA_THCA	high grade *vs.* low grade	-0.58	DOWN	EXP00402
hsa-miR-148a-5p	TCGA_THCA	high grade *vs.* low grade	-0.58	DOWN	EXP00402
hsa-miR-143-3p	TCGA_THCA	cancer *vs.* normal	-0.59	DOWN	EXP00396
hsa-miR-424-5p	TCGA_THCA	high grade *vs.* low grade	-0.59	DOWN	EXP00401
hsa-miR-374b-5p	TCGA_THCA	cancer *vs.* normal	-0.6	DOWN	EXP00396
hsa-miR-1180-3p	TCGA_THCA	high grade *vs.* low grade	-0.6	DOWN	EXP00402
hsa-miR-30a-3p	TCGA_THCA	cancer *vs.* normal	-0.61	DOWN	EXP00396
hsa-let-7b-5p	TCGA_THCA	cancer *vs.* normal	-0.61	DOWN	EXP00396
hsa-miR-30c-2-3p	TCGA_THCA	cancer *vs.* normal	-0.61	DOWN	EXP00396
hsa-miR-345-3p	TCGA_THCA	high grade *vs.* low grade	-0.61	DOWN	EXP00401
hsa-miR-126-3p	TCGA_THCA	high grade *vs.* low grade	-0.61	DOWN	EXP00401
hsa-miR-144-5p	TCGA_THCA	high grade *vs.* low grade	-0.61	DOWN	EXP00401
hsa-miR-144-5p	TCGA_THCA	high grade *vs.* low grade	-0.61	DOWN	EXP00402
hsa-miR-497-5p	TCGA_THCA	cancer *vs.* normal	-0.62	DOWN	EXP00396
hsa-miR-155-5p	TCGA_THCA	high grade *vs.* low grade	-0.62	DOWN	EXP00398
hsa-miR-19b	GSE40807	cancer *vs.* normal	-0.63	DOWN	EXP00274
hsa-miR-342-3p	TCGA_THCA	cancer *vs.* normal	-0.63	DOWN	EXP00396
hsa-miR-345-5p	TCGA_THCA	cancer *vs.* normal	-0.63	DOWN	EXP00396
hsa-miR-577	TCGA_THCA	high grade *vs.* low grade	-0.63	DOWN	EXP00402
hsa-miR-450b-5p	TCGA_THCA	high grade *vs.* low grade	-0.63	DOWN	EXP00402
hsa-miR-15b	GSE40807	cancer *vs.* normal	-0.64	DOWN	EXP00274
hsa-miR-551b-3p	TCGA_THCA	high grade *vs.* low grade	-0.65	DOWN	EXP00398
hsa-miR-652-3p	TCGA_THCA	high grade *vs.* low grade	-0.65	DOWN	EXP00402
hsa-miR-1179	TCGA_THCA	high grade *vs.* low grade	-0.65	DOWN	EXP00402
hsa-miR-577	TCGA_THCA	cancer *vs.* normal	-0.66	DOWN	EXP00396
hsa-miR-214-5p	TCGA_THCA	cancer *vs.* normal	-0.67	DOWN	EXP00396
hsa-miR-30c-2-3p	TCGA_THCA	high grade *vs.* low grade	-0.67	DOWN	EXP00401
hsa-miR-3607-3p	TCGA_THCA	high grade *vs.* low grade	-0.67	DOWN	EXP00401
hsa-miR-345-3p	TCGA_THCA	high grade *vs.* low grade	-0.67	DOWN	EXP00402
hsa-miR-30a-3p	TCGA_THCA	high grade *vs.* low grade	-0.67	DOWN	EXP00402
hsa-let-7f	GSE40807	cancer *vs.* normal	-0.68	DOWN	EXP00274
hsa-miR-19b-1*	GSE40807	cancer *vs.* normal	-0.68	DOWN	EXP00274
hsa-miR-21-5p	TCGA_THCA	high grade *vs.* low grade	-0.68	DOWN	EXP00398
hsa-miR-21-3p	TCGA_THCA	high grade *vs.* low grade	-0.68	DOWN	EXP00398
hsa-miR-450b-5p	TCGA_THCA	high grade *vs.* low grade	-0.68	DOWN	EXP00401
hsa-miR-7-5p	TCGA_THCA	high grade *vs.* low grade	-0.68	DOWN	EXP00402

hsa-miR-455-5p	TCGA_THCA	cancer *vs.* normal	-0.69	DOWN	EXP00396
hsa-miR-146a-5p	TCGA_THCA	high grade *vs.* low grade	-0.69	DOWN	EXP00398
hsa-miR-874-3p	TCGA_THCA	high grade *vs.* low grade	-0.69	DOWN	EXP00401
hsa-miR-30a-5p	TCGA_THCA	cancer *vs.* normal	-0.7	DOWN	EXP00396
hsa-miR-139-5p	TCGA_THCA	cancer *vs.* normal	-0.7	DOWN	EXP00396
hsa-miR-1247-3p	TCGA_THCA	high grade *vs.* low grade	-0.7	DOWN	EXP00398
hsa-miR-126-5p	TCGA_THCA	cancer *vs.* normal	-0.71	DOWN	EXP00396
hsa-miR-199a-5p	TCGA_THCA	high grade *vs.* low grade	-0.71	DOWN	EXP00398
hsa-miR-193a-5p	TCGA_THCA	cancer *vs.* normal	-0.72	DOWN	EXP00396
hsa-miR-145-5p	TCGA_THCA	cancer *vs.* normal	-0.72	DOWN	EXP00396
hsa-miR-152-3p	TCGA_THCA	high grade *vs.* low grade	-0.72	DOWN	EXP00401
hsa-miR-708	GSE40807	cancer *vs.* normal	-0.73	DOWN	EXP00274
hsa-miR-99a-5p	TCGA_THCA	cancer *vs.* normal	-0.73	DOWN	EXP00396
hsa-miR-183-5p	TCGA_THCA	high grade *vs.* low grade	-0.73	DOWN	EXP00401
hsa-miR-1251-5p	TCGA_THCA	high grade *vs.* low grade	-0.73	DOWN	EXP00402
hsa-miR-1-3p	TCGA_THCA	cancer *vs.* normal	-0.74	DOWN	EXP00396
hsa-miR-126-5p	TCGA_THCA	high grade *vs.* low grade	-0.74	DOWN	EXP00401
hsa-miR-3074-5p	TCGA_THCA	high grade *vs.* low grade	-0.74	DOWN	EXP00402
hsa-miR-92a	GSE40807	cancer *vs.* normal	-0.75	DOWN	EXP00274
hsa-miR-455-3p	TCGA_THCA	cancer *vs.* normal	-0.75	DOWN	EXP00396
hsa-miR-142-5p	TCGA_THCA	high grade *vs.* low grade	-0.75	DOWN	EXP00398
hsa-miR-142-5p	TCGA_THCA	cancer *vs.* normal	-0.76	DOWN	EXP00396
hsa-miR-1251-5p	TCGA_THCA	high grade *vs.* low grade	-0.76	DOWN	EXP00401
hsa-miR-126-3p	TCGA_THCA	high grade *vs.* low grade	-0.76	DOWN	EXP00402
hsa-miR-139-3p	TCGA_THCA	cancer *vs.* normal	-0.77	DOWN	EXP00396
hsa-miR-193b-3p	TCGA_THCA	cancer *vs.* normal	-0.78	DOWN	EXP00396
hsa-miR-708-3p	TCGA_THCA	cancer *vs.* normal	-0.78	DOWN	EXP00396
hsa-miR-150-5p	TCGA_THCA	high grade *vs.* low grade	-0.78	DOWN	EXP00398
hsa-miR-214-3p	TCGA_THCA	cancer *vs.* normal	-0.8	DOWN	EXP00396
hsa-miR-345-3p	TCGA_THCA	cancer *vs.* normal	-0.8	DOWN	EXP00396
hsa-miR-218-5p	TCGA_THCA	cancer *vs.* normal	-0.8	DOWN	EXP00396
hsa-miR-10a-5p	TCGA_THCA	cancer *vs.* normal	-0.8	DOWN	EXP00396
hsa-miR-337-3p	TCGA_THCA	cancer *vs.* normal	-0.82	DOWN	EXP00396
hsa-miR-381-3p	TCGA_THCA	cancer *vs.* normal	-0.82	DOWN	EXP00396
hsa-miR-542-3p	TCGA_THCA	high grade *vs.* low grade	-0.82	DOWN	EXP00401
hsa-miR-874-3p	TCGA_THCA	high grade *vs.* low grade	-0.82	DOWN	EXP00402
hsa-miR-183-5p	TCGA_THCA	high grade *vs.* low grade	-0.82	DOWN	EXP00402
hsa-miR-298	GSE40807	cancer *vs.* normal	-0.83	DOWN	EXP00274
hsa-miR-139-5p	TCGA_THCA	high grade *vs.* low grade	-0.83	DOWN	EXP00401
hsa-miR-6715a-3p	TCGA_THCA	high grade *vs.* low grade	-0.83	DOWN	EXP00401
hsa-miR-26a-5p	TCGA_THCA	cancer *vs.* normal	-0.84	DOWN	EXP00396
hsa-miR-142-3p	TCGA_THCA	high grade *vs.* low grade	-0.84	DOWN	EXP00398
hsa-miR-629	GSE40807	cancer *vs.* normal	-0.85	DOWN	EXP00274
hsa-miR-199a-5p	TCGA_THCA	cancer *vs.* normal	-0.85	DOWN	EXP00396
hsa-miR-31-3p	TCGA_THCA	high grade *vs.* low grade	-0.85	DOWN	EXP00398
hsa-miR-7-2-3p	TCGA_THCA	cancer *vs.* normal	-0.86	DOWN	EXP00396
hsa-miR-100-5p	TCGA_THCA	cancer *vs.* normal	-0.86	DOWN	EXP00396
hsa-miR-134-5p	TCGA_THCA	cancer *vs.* normal	-0.86	DOWN	EXP00396
hsa-miR-542-3p	TCGA_THCA	high grade *vs.* low grade	-0.87	DOWN	EXP00402
hsa-let-7g	GSE40807	cancer *vs.* normal	-0.88	DOWN	EXP00274
hsa-miR-152-3p	TCGA_THCA	high grade *vs.* low grade	-0.88	DOWN	EXP00402

hsa-miR-222-3p	TCGA_THCA	high grade *vs.* low grade	-0.89	DOWN	EXP00398
hsa-miR-126-5p	TCGA_THCA	high grade *vs.* low grade	-0.89	DOWN	EXP00402
hsa-miR-153-5p	TCGA_THCA	cancer *vs.* normal	-0.9	DOWN	EXP00396
hsa-miR-30c-2-3p	TCGA_THCA	high grade *vs.* low grade	-0.9	DOWN	EXP00402
hsa-miR-199a-5p	GSE40807	cancer *vs.* normal	-0.92	DOWN	EXP00274
hsa-miR-195-5p	TCGA_THCA	cancer *vs.* normal	-0.92	DOWN	EXP00396
hsa-miR-221-3p	TCGA_THCA	high grade *vs.* low grade	-0.92	DOWN	EXP00398
hsa-miR-28-3p	TCGA_THCA	cancer *vs.* normal	-0.93	DOWN	EXP00396
hsa-miR-190a-5p	TCGA_THCA	cancer *vs.* normal	-0.94	DOWN	EXP00396
hsa-miR-223-3p	TCGA_THCA	cancer *vs.* normal	-0.94	DOWN	EXP00396
hsa-miR-3074-5p	TCGA_THCA	cancer *vs.* normal	-0.95	DOWN	EXP00396
hsa-miR-138-1-3p	TCGA_THCA	cancer *vs.* normal	-0.95	DOWN	EXP00396
hsa-miR-20b-5p	TCGA_THCA	cancer *vs.* normal	-0.96	DOWN	EXP00396
hsa-miR-363-3p	TCGA_THCA	cancer *vs.* normal	-0.98	DOWN	EXP00396
hsa-miR-138-5p	TCGA_THCA	cancer *vs.* normal	-0.98	DOWN	EXP00396
hsa-miR-204-5p	TCGA_THCA	high grade *vs.* low grade	-0.98	DOWN	EXP00400
hsa-miR-27a-5p	TCGA_THCA	cancer *vs.* normal	-0.99	DOWN	EXP00396
hsa-miR-7-5p	TCGA_THCA	cancer *vs.* normal	-1	DOWN	EXP00396
hsa-miR-708-5p	TCGA_THCA	cancer *vs.* normal	-1	DOWN	EXP00396
hsa-miR-142-3p	TCGA_THCA	cancer *vs.* normal	-1.01	DOWN	EXP00396
hsa-miR-139-3p	TCGA_THCA	high grade *vs.* low grade	-1.01	DOWN	EXP00401
hsa-miR-126	GSE40807	cancer *vs.* normal	-1.02	DOWN	EXP00274
hsa-miR-139-5p	TCGA_THCA	high grade *vs.* low grade	-1.02	DOWN	EXP00402
hsa-miR-345-5p	TCGA_THCA	high grade *vs.* low grade	-1.03	DOWN	EXP00401
hsa-miR-6715a-3p	TCGA_THCA	high grade *vs.* low grade	-1.04	DOWN	EXP00402
hsa-miR-31-5p	TCGA_THCA	high grade *vs.* low grade	-1.05	DOWN	EXP00398
hsa-miR-152-3p	TCGA_THCA	cancer *vs.* normal	-1.08	DOWN	EXP00396
hsa-miR-139-3p	TCGA_THCA	high grade *vs.* low grade	-1.09	DOWN	EXP00402
hsa-miR-17	GSE40807	cancer *vs.* normal	-1.1	DOWN	EXP00274
hsa-miR-127-3p	TCGA_THCA	cancer *vs.* normal	-1.1	DOWN	EXP00396
hsa-miR-30a	GSE40807	cancer *vs.* normal	-1.13	DOWN	EXP00274
hsa-miR-886-3p	GSE40807	cancer *vs.* normal	-1.14	DOWN	EXP00274
hsa-miR-652-3p	TCGA_THCA	cancer *vs.* normal	-1.15	DOWN	EXP00396
hsa-miR-20a	GSE40807	cancer *vs.* normal	-1.16	DOWN	EXP00274
hsa-miR-345-5p	TCGA_THCA	high grade *vs.* low grade	-1.16	DOWN	EXP00402
hsa-let-7i	GSE40807	cancer *vs.* normal	-1.17	DOWN	EXP00274
hsa-miR-101*	GSE40807	cancer *vs.* normal	-1.21	DOWN	EXP00274
hsa-miR-204-5p	TCGA_THCA	high grade *vs.* low grade	-1.22	DOWN	EXP00401
hsa-miR-508-5p	GSE40807	cancer *vs.* normal	-1.25	DOWN	EXP00274
hsa-miR-1247-5p	TCGA_THCA	cancer *vs.* normal	-1.3	DOWN	EXP00396
hsa-miR-205-5p	TCGA_THCA	high grade *vs.* low grade	-1.31	DOWN	EXP00398
hsa-miR-675-3p	TCGA_THCA	cancer *vs.* normal	-1.34	DOWN	EXP00396
hsa-miR-142-3p	GSE40807	cancer *vs.* normal	-1.36	DOWN	EXP00274
hsa-miR-193b	GSE40807	cancer *vs.* normal	-1.37	DOWN	EXP00274
hsa-miR-150-5p	TCGA_THCA	cancer *vs.* normal	-1.4	DOWN	EXP00396
hsa-miR-193a-3p	GSE40807	cancer *vs.* normal	-1.41	DOWN	EXP00274
hsa-miR-199a-3p	GSE40807	cancer *vs.* normal	-1.41	DOWN	EXP00274
hsa-miR-199a-3p	TCGA_THCA	cancer *vs.* normal	-1.42	DOWN	EXP00396
hsa-miR-375	TCGA_THCA	high grade *vs.* low grade	-1.44	DOWN	EXP00398
hsa-miR-130a	GSE40807	cancer *vs.* normal	-1.48	DOWN	EXP00274
hsa-miR-199b-3p	TCGA_THCA	cancer *vs.* normal	-1.5	DOWN	EXP00396

hsa-miR-1179	TCGA_THCA	cancer *vs.* normal	-1.53	DOWN	EXP00396
hsa-miR-199b-5p	TCGA_THCA	high grade *vs.* low grade	-1.53	DOWN	EXP00398
hsa-miR-499-5p	GSE40807	cancer *vs.* normal	-1.55	DOWN	EXP00274
hsa-miR-146b-5p	TCGA_THCA	high grade *vs.* low grade	-1.55	DOWN	EXP00398
hsa-miR-1247-3p	TCGA_THCA	cancer *vs.* normal	-1.56	DOWN	EXP00396
hsa-miR-146b-3p	TCGA_THCA	high grade *vs.* low grade	-1.56	DOWN	EXP00398
hsa-miR-146a	GSE40807	cancer *vs.* normal	-1.57	DOWN	EXP00274
hsa-miR-223	GSE40807	cancer *vs.* normal	-1.58	DOWN	EXP00274
hsa-miR-379-5p	TCGA_THCA	cancer *vs.* normal	-1.58	DOWN	EXP00396
hsa-miR-144-3p	TCGA_THCA	cancer *vs.* normal	-1.6	DOWN	EXP00396
hsa-miR-152	GSE40807	cancer *vs.* normal	-1.74	DOWN	EXP00274
hsa-miR-218	GSE40807	cancer *vs.* normal	-1.77	DOWN	EXP00274
hsa-miR-486-5p	GSE40807	cancer *vs.* normal	-1.78	DOWN	EXP00274
hsa-miR-584	GSE40807	cancer *vs.* normal	-1.79	DOWN	EXP00274
hsa-miR-20a*	GSE40807	cancer *vs.* normal	-1.79	DOWN	EXP00274
hsa-miR-32*	GSE40807	cancer *vs.* normal	-1.82	DOWN	EXP00274
hsa-miR-204-5p	TCGA_THCA	high grade *vs.* low grade	-1.83	DOWN	EXP00402
hsa-miR-181a-2*	GSE40807	cancer *vs.* normal	-1.84	DOWN	EXP00274
hsa-miR-193b*	GSE40807	cancer *vs.* normal	-1.84	DOWN	EXP00274
hsa-miR-629*	GSE40807	cancer *vs.* normal	-1.85	DOWN	EXP00274
hsa-miR-30e*	GSE40807	cancer *vs.* normal	-1.87	DOWN	EXP00274
hsa-miR-20b	GSE40807	cancer *vs.* normal	-1.91	DOWN	EXP00274
hsa-miR-144	GSE40807	cancer *vs.* normal	-1.93	DOWN	EXP00274
hsa-miR-9-5p	TCGA_THCA	cancer *vs.* normal	-1.93	DOWN	EXP00396
hsa-miR-219-5p	GSE40807	cancer *vs.* normal	-1.94	DOWN	EXP00274
hsa-miR-10b*	GSE40807	cancer *vs.* normal	-1.94	DOWN	EXP00274
hsa-miR-30c-2*	GSE40807	cancer *vs.* normal	-1.97	DOWN	EXP00274
hsa-miR-887	GSE40807	cancer *vs.* normal	-1.99	DOWN	EXP00274
hsa-miR-214*	GSE40807	cancer *vs.* normal	-2	DOWN	EXP00274
hsa-miR-204-5p	TCGA_THCA	cancer *vs.* normal	-2.13	DOWN	EXP00396
hsa-miR-199b-5p	TCGA_THCA	cancer *vs.* normal	-2.18	DOWN	EXP00396
hsa-miR-451	GSE40807	cancer *vs.* normal	-2.21	DOWN	EXP00274
hsa-miR-30a*	GSE40807	cancer *vs.* normal	-2.24	DOWN	EXP00274
hsa-miR-18b	GSE40807	cancer *vs.* normal	-2.26	DOWN	EXP00274
hsa-miR-133b	GSE40807	cancer *vs.* normal	-2.27	DOWN	EXP00274
hsa-let-7i*	GSE40807	cancer *vs.* normal	-2.37	DOWN	EXP00274
hsa-miR-133a	GSE40807	cancer *vs.* normal	-2.38	DOWN	EXP00274
hsa-miR-18a	GSE40807	cancer *vs.* normal	-2.41	DOWN	EXP00274
hsa-miR-424*	GSE40807	cancer *vs.* normal	-2.42	DOWN	EXP00274
hsa-miR-138	GSE40807	cancer *vs.* normal	-2.53	DOWN	EXP00274
hsa-miR-7-2*	GSE40807	cancer *vs.* normal	-2.54	DOWN	EXP00274
hsa-miR-486-5p	TCGA_THCA	cancer *vs.* normal	-2.55	DOWN	EXP00396
hsa-miR-345	GSE40807	cancer *vs.* normal	-2.56	DOWN	EXP00274
hsa-miR-451a	TCGA_THCA	cancer *vs.* normal	-2.57	DOWN	EXP00396
hsa-miR-144-5p	TCGA_THCA	cancer *vs.* normal	-2.61	DOWN	EXP00396
hsa-miR-455-5p	GSE40807	cancer *vs.* normal	-2.65	DOWN	EXP00274
hsa-miR-625	GSE40807	cancer *vs.* normal	-2.67	DOWN	EXP00274
hsa-miR-455-3p	GSE40807	cancer *vs.* normal	-2.68	DOWN	EXP00274
hsa-miR-551b	GSE40807	cancer *vs.* normal	-2.78	DOWN	EXP00274
hsa-miR-31	GSE40807	cancer *vs.* normal	-2.8	DOWN	EXP00274
hsa-miR-190	GSE40807	cancer *vs.* normal	-2.82	DOWN	EXP00274

hsa-miR-16-2*	GSE40807	cancer *vs*. normal	-2.85	DOWN	EXP00274
hsa-miR-150	GSE40807	cancer *vs*. normal	-2.96	DOWN	EXP00274
hsa-miR-363	GSE40807	cancer *vs*. normal	-3.06	DOWN	EXP00274
hsa-miR-204	GSE40807	cancer *vs*. normal	-3.2	DOWN	EXP00274
hsa-miR-205	GSE40807	cancer *vs*. normal	-3.24	DOWN	EXP00274
hsa-miR-203	GSE40807	cancer *vs*. normal	-3.25	DOWN	EXP00274
hsa-miR-199b-5p	GSE40807	cancer *vs*. normal	-3.32	DOWN	EXP00274
hsa-miR-144*	GSE40807	cancer *vs*. normal	-3.34	DOWN	EXP00274
hsa-miR-31*	GSE40807	cancer *vs*. normal	-3.52	DOWN	EXP00274

Data source: dbDEMC (https://www.biosino.org/dbDEMC/index).

Supplementary Table S5. Pathway enrichment analysis of 104 deregulated microRNAs in thyroid cancer

KEGG pathway	KEGG ID	p-value	# target genes
Pathways in cancer	hsa05200	<1e-325	178
Proteoglycans in cancer	hsa05205	<1e-325	168
Viral carcinogenesis	hsa05203	<1e-325	160
Hippo signaling pathway	hsa04390	<1e-325	111
Cell cycle	hsa04110	<1e-325	101
Hepatitis B	hsa05161	<1e-325	92
Focal adhesion	hsa04510	1.44E-15	87
Thyroid hormone signaling pathway	hsa04919	<1e-325	72
Transcriptional misregulation in cancer	hsa05202	1.76E-11	69
Signaling pathways regulating pluripotency of stem cells	hsa04550	<1e-325	64
Oocyte meiosis	hsa04114	8.88E-16	63
Adherens junction	hsa04520	<1e-325	62
FoxO signaling pathway	hsa04068	<1e-325	60
ECM-receptor interaction	hsa04512	<1e-325	56
TGF-beta signaling pathway	hsa04350	<1e-325	53
PI3K-Akt signaling pathway	hsa04151	1.05E-05	53
Prostate cancer	hsa05215	<1e-325	48
Bacterial invasion of epithelial cells	hsa05100	<1e-325	46
Ubiquitin mediated proteolysis	hsa04120	6.70E-08	43
Neurotrophin signaling pathway	hsa04722	0.00052	43
Chronic myeloid leukemia	hsa05220	<1e-325	42
Protein processing in endoplasmic reticulum	hsa04141	<1e-325	39
Glioma	hsa05214	<1e-325	38
Lysine degradation	hsa00310	<1e-325	35
Renal cell carcinoma	hsa05211	<1e-325	35
Fatty acid metabolism	hsa01212	<1e-325	33
Prolactin signaling pathway	hsa04917	7.72E-06	33
Colorectal cancer	hsa05210	<1e-325	31
Endometrial cancer	hsa05213	<1e-325	24
Pancreatic cancer	hsa05212	2.11E-15	24
Non-small cell lung cancer	hsa05223	1.58E-13	24
Prion diseases	hsa05020	<1e-325	20
Estrogen signaling pathway	hsa04915	1.01E-07	20
Bladder cancer	hsa05219	<1e-325	19
Melanoma	hsa05218	1.34E-13	19
Thyroid cancer	hsa05216	2.22E-16	18
p53 signaling pathway	hsa04115	<1e-325	15
Fatty acid elongation	hsa00062	0.0331	13
Endocytosis	hsa04144	1.45E-05	8
Fatty acid biosynthesis	hsa00061	<1e-325	7
Steroid biosynthesis	hsa00100	<1e-325	7

Data source: DIANA-miRPath v.3.0 (http://www.microrna.gr/miRPathv2).

Supplementary Table S6. Downregulated miR-204-5p in various cancer types

GEO ID	Cancer Type	Design	logFC	Experiment ID
GSE40355	bladder cancer	cancer *vs.* normal	-8.42	EXP00231
GSE11016	kidney cancer	cancer *vs.* normal	-7.59	EXP00058
TCGA_KICH	kidney cancer	cancer *vs.* normal	-5.44	EXP00368
GSE57370	kidney cancer	cancer *vs.* normal	-5.42	EXP00293
GSE45666	breast cancer	cancer *vs.* normal	-4.75	EXP00238
GSE33332	kidney cancer	cancer *vs.* normal	-4.65	EXP00204
TCGA_CESC	cervical cancer	cancer *vs.* normal	-4.39	EXP00411
GSE24996	melanoma	cancer *vs.* normal	-4.03	EXP00112
GSE18509	melanoma	metastasis	-3.9	EXP00089
GSE45264	lymphoma	cancer *vs.* normal	-3.84	EXP00216
GSE11016	kidney cancer	cancer *vs.* normal	-3.31	EXP00055
GSE40807	thyroid cancer	cancer *vs.* normal	-3.2	EXP00274
GSE50505	kidney cancer	cancer *vs.* normal	-3.15	EXP00261
TCGA_UCEC	endometrial cancer	cancer *vs.* normal	-3.03	EXP00386
TCGA_BLCA	bladder cancer	cancer *vs.* normal	-2.81	EXP00430
GSE6636	prostate cancer	cancer *vs.* normal	-2.77	EXP00032
GSE54397	gastric cancer	cancer *vs.* normal	-2.71	EXP00270
TCGA_ESCA	esophageal cancer	cancer *vs.* normal	-2.68	EXP00423
GSE26595	gastric cancer	cancer *vs.* normal	-2.43	EXP00230
TCGA_KICH	kidney cancer	high grade *vs.* low grade	-2.41	EXP00370
GSE2564	kidney cancer	cancer *vs.* normal	-2.3	EXP00019
GSE25820	pancreatic cancer	cancer *vs.* normal	-2.24	EXP00135
GSE28700	gastric cancer	cancer *vs.* normal	-2.19	EXP00118
GSE47841	ovarian cancer	cancer *vs.* normal	-2.17	EXP00259
TCGA_KIRC	kidney cancer	cancer *vs.* normal	-2.16	EXP00362
GSE21036	prostate cancer	metastasis	-2.16	EXP00102
TCGA_THCA	thyroid cancer	cancer *vs.* normal	-2.13	EXP00396
GSE20077	hepatocellular carcinoma	cancer *vs.* normal	-2.07	EXP00115
GSE54397	gastric cancer	cancer *vs.* normal	-2.04	EXP00268
GSE38167	breast cancer	cancer *vs.* normal	-1.95	EXP00273
GSE47582	kidney cancer	cancer *vs.* normal	-1.92	EXP00233
GSE12105	kidney cancer	cancer *vs.* normal	-1.85	EXP00062
TCGA_STAD	gastric cancer	cancer *vs.* normal	-1.83	EXP00404
TCGA_THCA	thyroid cancer	high grade *vs.* low grade	-1.83	EXP00402
TCGA_HNSC	head and neck cancer	cancer *vs.* normal	-1.77	EXP00393
GSE31277	head and neck cancer	cancer *vs.* normal	-1.39	EXP00272
GSE36802	prostate cancer	cancer *vs.* normal	-1.25	EXP00212
TCGA_THCA	thyroid cancer	high grade *vs.* low grade	-1.22	EXP00401
GSE28955	pancreatic cancer	cancer *vs.* normal	-1.12	EXP00160
GSE40525	breast cancer	cancer *vs.* normal	-1.08	EXP00197
TCGA_COAD	colon cancer	cancer *vs.* normal	-1.02	EXP00387
GSE26245	prostate cancer	cancer *vs.* normal	-1.02	EXP00120
GSE30454	colorectal cancer	cancer *vs.* normal	-1.01	EXP00141
TCGA_BRCA	breast cancer	cancer *vs.* normal	-1	EXP00352

GSE21036	prostate cancer	cancer *vs.* normal	-1	EXP00101
TCGA_PRAD	prostate cancer	cancer *vs.* normal	-0.99	EXP00403
GSE6188	esophageal cancer	cancer *vs.* normal	-0.98	EXP00031
TCGA_THCA	thyroid cancer	high grade *vs.* low grade	-0.98	EXP00400
GSE40525	breast cancer	cancer *vs.* normal	-0.96	EXP00190
TCGA_KIRC	kidney cancer	high grade *vs.* low grade	-0.95	EXP00365
TCGA_KIRP	kidney cancer	cancer *vs.* normal	-0.93	EXP00356
GSE36915	hepatocellular carcinoma	cancer *vs.* normal	-0.9	EXP00221
TCGA_KIRP	kidney cancer	high grade *vs.* low grade	-0.9	EXP00358
TCGA_LUAD	lung cancer	cancer *vs.* normal	-0.9	EXP00374
TCGA_PAAD	pancreatic cancer	high grade *vs.* low grade	-0.88	EXP00417
GSE44124	breast cancer	cancer *vs.* normal	-0.85	EXP00258
GSE5244	uterus cancer	cancer *vs.* normal	-0.79	EXP00030
GSE23739	gastric cancer	cancer *vs.* normal	-0.75	EXP00131
GSE22216	breast cancer	high grade *vs.* low grade	-0.69	EXP00126
GSE18546	sarcoma	cancer *vs.* normal	-0.67	EXP00111
TCGA_KIRC	kidney cancer	high grade *vs.* low grade	-0.64	EXP00364
GSE18392	colon cancer	cancer *vs.* normal	-0.51	EXP00091
GSE38781	pancreatic cancer	poor outcome *vs.* good outcome	-0.51	EXP00202
GSE45604	prostate cancer	cancer *vs.* normal	-0.49	EXP00217
GSE36681	lung cancer	cancer *vs.* normal	-0.43	EXP00176
GSE48137	kidney cancer	blood	-0.4	EXP00332
GSE36682	nasopharyngeal cancer	cancer *vs.* normal	-0.4	EXP00163
GSE32232	lymphoma	cancer *vs.* normal	-0.33	EXP00133
GSE49246	colon cancer	cancer *vs.* normal	-0.3	EXP00246
GSE22058	hepatocellular carcinoma	cancer *vs.* normal	-0.16	EXP00100
GSE6857	hepatocellular carcinoma	cancer *vs.* normal	-0.13	EXP00081
GSE19387	melanoma	metastasis	-0.11	EXP00099
GSE38389	colorectal cancer	cancer *vs.* normal	-0.09	EXP00184

Supplementary Table S7. Upregulated miR-221-3p in various cancer types

GEO ID	Cancer Type (cell line)	Design	logFC	Experiment ID
GSE20077	hepatocellular carcinoma	cancer *vs*. normal	4.85	EXP00115
GSE65819	ovarian cancer	cancer *vs*. normal	4.3	EXP00301
TCGA_KICH	kidney cancer	cancer *vs*. normal	4	EXP00368
GSE11016	kidney cancer	cancer *vs*. normal	3.62	EXP00058
GSE28955	pancreatic cancer	cancer *vs*. normal	3.18	EXP00160
GSE13030	brain cancer	cancer *vs*. normal	3.16	EXP00066
GSE11016	kidney cancer	cancer *vs*. normal	3.15	EXP00057
TCGA_THCA	thyroid cancer	cancer *vs*. normal	2.95	EXP00396
GSE53992	biliary tract cancer	cancer *vs*. normal	2.3	EXP00290
GSE40807	thyroid cancer	cancer *vs*. normal	2.3	EXP00274
GSE13030	brain cancer	cancer *vs*. normal	2.21	EXP00065
GSE32957	liver cancer	cancer *vs*. normal	2.2	EXP00181
GSE41369	pancreatic cancer	cancer *vs*. normal	2.14	EXP00248
GSE32957	liver cancer	cancer *vs*. normal	2.01	EXP00179
GSE25820	pancreatic cancer	cancer *vs*. normal	1.9	EXP00135
TCGA_CHOL	biliary tract cancer	cancer *vs*. normal	1.83	EXP00425
GSE2564	lymphoma	subtype1 *vs*. substype2	1.8	EXP00028
GSE74190	lung cancer	cancer *vs*. normal	1.7	EXP00310
GSE37053	lymphoma	subtype1 *vs*. substype2	1.68	EXP00225
TCGA_LIHC	hepatocellular carcinoma	cancer *vs*. normal	1.66	EXP00409
GSE2564	colon cancer	cancer *vs*. normal	1.65	EXP00021
GSE10694	hepatocellular carcinoma	cancer *vs*. normal	1.65	EXP00051
GSE40744	hepatocellular carcinoma	cancer *vs*. normal	1.59	EXP00213
GSE10694	hepatocellular carcinoma	cancer *vs*. normal	1.47	EXP00052
GSE39678	hepatocellular carcinoma	cancer *vs*. normal	1.44	EXP00183
GSE25820	pancreatic cancer	cancer *vs*. normal	1.4	EXP00136
TCGA_THCA	thyroid cancer	high grade *vs*. low grade	1.35	EXP00402
GSE2564	leukemia	subtype1 *vs*. substype2	1.33	EXP00026
GSE31568	melanoma	blood	1.24	EXP00323
GSE54397	gastric cancer	cancer *vs*. normal	1.23	EXP00270
GSE6857	hepatocellular carcinoma	cancer *vs*. normal	1.21	EXP00081
GSE51853	lung cancer	cancer *vs*. normal	1.2	EXP00286
GSE21362	hepatocellular carcinoma	cancer *vs*. normal	1.17	EXP00117
GSE47841	ovarian cancer	cancer *vs*. normal	1.13	EXP00260
GSE56183	chordoma	cancer *vs*. normal	1.11	EXP00291
GSE35982	colorectal cancer	cancer *vs*. normal	1.1	EXP00207
TCGA_THCA	thyroid cancer	high grade *vs*. low grade	1.05	EXP00401
GSE51908	leukemia	cancer *vs*. normal	1.02	EXP00265
GSE11163	head and neck cancer	cancer *vs*. normal	1	EXP00061
GSE2399	Leukemia (Jurkat)	subtype1 *vs*. substype2	0.89	EXP00017
GSE60978	pancreatic cancer	cancer *vs*. normal	0.85	EXP00296
GSE51853	lung cancer	cancer *vs*. normal	0.84	EXP00288
GSE49246	colon cancer	cancer *vs*. normal	0.81	EXP00246

GSE40525	breast cancer	cancer *vs.* normal	0.77	EXP00191
GSE28700	gastric cancer	cancer *vs.* normal	0.7	EXP00118
TCGA_STAD	gastric cancer	cancer *vs.* normal	0.67	EXP00404
GSE36915	hepatocellular carcinoma	cancer *vs.* normal	0.66	EXP00221
GSE10259	colorectal cancer	cancer *vs.* normal	0.65	EXP00050
GSE23739	gastric cancer	cancer *vs.* normal	0.62	EXP00131
GSE40525	breast cancer	cancer *vs.* normal	0.51	EXP00195
GSE35834	colon cancer	cancer *vs.* normal	0.47	EXP00253
GSE33232	head and neck cancer	cancer *vs.* normal	0.39	EXP00206
GSE22058	hepatocellular carcinoma	cancer *vs.* normal	0.34	EXP00100
GSE45238	oral squamous cell carcinoma	cancer *vs.* normal	0.31	EXP00281
GSE22216	breast cancer	high grade *vs.* low grade	0.3	EXP00126
GSE7828	colon cancer	cancer *vs.* normal	0.28	EXP00084
GSE16025	lung cancer	cancer *vs.* normal	0.12	EXP00074
GSE38389	colorectal cancer	cancer *vs.* normal	0.11	EXP00184
GSE38389	colorectal cancer	cancer *vs.* normal	0.11	EXP00184

Supplementary Table S8. Upregulated miR-222-3p in various cancer types

GEO ID	Cancer Type	Design	logFC	Experiment ID
GSE12933	lymphoma	subtype1 *vs.* substype2	5.44	EXP00064
GSE11016	kidney cancer	cancer *vs.* normal	4.13	EXP00058
GSE28955	pancreatic cancer	cancer *vs.* normal	3.82	EXP00160
GSE11016	kidney cancer	cancer *vs.* normal	3.77	EXP00057
GSE25820	pancreatic cancer	cancer *vs.* normal	3.61	EXP00135
GSE53992	biliary tract cancer	cancer *vs.* normal	3.3	EXP00290
TCGA_KICH	kidney cancer	cancer *vs.* normal	3.2	EXP00368
GSE37053	lymphoma	subtype1 *vs.* substype2	2.88	EXP00225
TCGA_THCA	thyroid cancer	cancer *vs.* normal	2.69	EXP00396
GSE25820	pancreatic cancer	cancer *vs.* normal	2.16	EXP00136
TCGA_CHOL	biliary tract cancer	cancer *vs.* normal	1.98	EXP00425
GSE40807	thyroid cancer	cancer *vs.* normal	1.97	EXP00274
GSE32678	pancreatic cancer	cancer *vs.* normal	1.9	EXP00137
GSE32957	liver cancer	cancer *vs.* normal	1.78	EXP00180
GSE56183	chordoma	cancer *vs.* normal	1.72	EXP00291
GSE2564	leukemia	subtype1 *vs.* substype2	1.66	EXP00026
GSE39678	hepatocellular carcinoma	cancer *vs.* normal	1.66	EXP00183
GSE25820	pancreatic cancer	cancer *vs.* normal	1.59	EXP00134
GSE65071	sarcoma	blood	1.57	EXP00340
GSE41369	pancreatic cancer	cancer *vs.* normal	1.53	EXP00248
GSE10694	hepatocellular carcinoma	cancer *vs.* normal	1.52	EXP00051
GSE40744	hepatocellular carcinoma	cancer *vs.* normal	1.49	EXP00213
TCGA_THCA	thyroid cancer	high grade *vs.* low grade	1.38	EXP00402
GSE10694	hepatocellular carcinoma	cancer *vs.* normal	1.31	EXP00052
GSE41321	retinoblastoma	blood	1.23	EXP00330
GSE41032	brain cancer	cancer *vs.* normal	1.2	EXP00276
GSE51908	leukemia	cancer *vs.* normal	1.15	EXP00265
TCGA_LIHC	hepatocellular carcinoma	cancer *vs.* normal	1.07	EXP00409
TCGA_THCA	thyroid cancer	high grade *vs.* low grade	1.03	EXP00401
GSE36915	hepatocellular carcinoma	cancer *vs.* normal	0.94	EXP00221
TCGA_STAD	gastric cancer	cancer *vs.* normal	0.93	EXP00404
GSE2399	Leukemia (Jurkat)	subtype1 *vs.* substype2	0.85	EXP00017
GSE21362	hepatocellular carcinoma	cancer *vs.* normal	0.82	EXP00117
TCGA_KIRC	kidney cancer	cancer *vs.* normal	0.8	EXP00362
GSE2399	Lymphoma (HG-1125)	cancer *vs.* normal	0.76	EXP00008
GSE47841	ovarian cancer	cancer *vs.* normal	0.75	EXP00260
GSE40345	mesothelioma	cancer *vs.* normal	0.73	EXP00234
TCGA_HNSC	head and neck cancer	cancer *vs.* normal	0.73	EXP00393
GSE6857	hepatocellular carcinoma	cancer *vs.* normal	0.71	EXP00081
TCGA_KIRP	kidney cancer	cancer *vs.* normal	0.69	EXP00356
TCGA_KIRP	kidney cancer	high grade *vs.* low grade	0.68	EXP00360
GSE28700	gastric cancer	cancer *vs.* normal	0.63	EXP00118

GSE45238	oral squamous cell carcinoma	cancer *vs.* normal	0.57	EXP00281
GSE22216	breast cancer	high grade *vs.* low grade	0.47	EXP00126
GSE39486	brain cancer	cancer *vs.* normal	0.44	EXP00205
GSE49246	colon cancer	cancer *vs.* normal	0.43	EXP00246
TCGA_KIRP	kidney cancer	high grade *vs.* low grade	0.4	EXP00358
GSE7828	colon cancer	cancer *vs.* normal	0.35	EXP00084
GSE22058	hepatocellular carcinoma	cancer *vs.* normal	0.28	EXP00100
GSE74562	pancreatic cancer (PAN-1)	treatment	0.28	EXP00351
GSE5244	uterus cancer	cancer *vs.* normal	0.17	EXP00030

Table 9. Summary of molecular pathways regulating the study microRNAs in cancer

Cancer	Target genes	Pathways	Mechanism involved	Ref.
**miR-204-5p**				
Prostate cancer	ACSL4	NEAT1, miR-34a-5p and miR-204-5p axis	Drug resistance	[58]
Cholangiocarcinoma	HMGB1	miR-204-5p/HMGB1 axis	Apoptosis, autophagy, and proliferation	[59]
Esophageal squamous cell carcinoma		IL-11 axis	Proliferation, invasion, and apoptosis	[60]
Esophageal squamous cell carcinoma	bcl-2, bax, and cleaved caspase-3	linc-ROR/miR-204-5p/MDM2	Proliferation and apoptosis	[61]
Metastatic melanoma		MEK pathway	Progression and drug resistance	[62]
Gastric cancer	SLAMF6	miR-204-5p/MYH9 axis	Glycolysis, migration, and invasion	[63]
Cervical cancer	TFAP2A		Proliferation, migration, invasion and EMT process	[64]
Gastric cancer	DLX6-AS1	DLX6-AS1/miR-204-5p/OCT1 axis	Proliferation	[65]
Breast cancer	RRM2	DSCAM-AS1/miR-204-5p/RRM2 axis	Proliferation and invasion	[66]
Osteosarcoma	EBF2	Wnt signaling	Apoptosis, and migration	[67]
Gastric cancer	ZNF521		Proliferation and Apoptosis	[68]
Endometrial carcinoma	SF3B1, FBXW7, and BRD4		Progression	[69]
Breast cancer	PIK3CB	PI3K/Akt signaling	Growth, Metastasis, and Immune Microenvironment	[70]
Ovarian cancer	USP47	miR-204-5p/USP4 pathway	Drug resistance	[71]
Gastric cancer	CBFB	LINC01234/MiR-204-5p axis	Proliferation and apoptosis	[72]
Gastric cancer	EGFR	post-transcriptional pathway	Migration and proliferation	[73]
Melanoma	TRPM3	MAPK-ERK pathway	Drug resistance	[74]
Breast cancer	AP1S3, RACGAP1, ELOVL6, and LRRC59		Progression	[75]

Prostate cancer	BCL2	mitochondria-dependent apoptotic pathway	Apoptosis	[76]
Hepatocellular carcinoma	CCND1,BCL2, NOTCH1, and HDAC1		Progression	[77]
Hepatocellular carcinoma	SIRT1		Proliferation	[78]
Breast cancer	Six1		Proliferation, migration, invasion and EMT process	[79]
Papillary thyroid carcinoma	IGFBP5		Proliferation, and apoptosis	[28]
Colorectal cancer	RAB22A		Proliferation and invasion	[80]
Endometrial carcinoma	TrkB	JAK/STAT pathway	Growth, migration and invasion	[81]
**miR-221-3p**				
Hepatocellular carcinoma	C1QTNF1-AS1	JAK/STAT signaling pathway	Proliferation, invasion, migration and apoptosis	[82]
Medulloblastoma cells	EIF5A2, CDK4, Cyclin D1, Bcl-2 and Bad		Cell cycle arrest and apoptosis	[83]
Cervical squamous cell carcinoma	THBS2		Angiogenesis	[84]
Non-small cell lung cancer	p27		Cell cycle progression	[85]
Cervical squamous cell carcinoma	VASH1		Lymphatic metastasis	[86]
Epithelial ovarian cancer	ARF4		Proliferation and migration	[87]
Pancreatic cancer			Proliferation and apoptosis	[88]
Gastric carcinoma	PTEN	PTEN signaling	Proliferation, invasion, and migration	[89]
Cervical cancer	THBS2		Metastasis	[90]
Breast cancer	PAK1		Drug resistance	[91]
**miR-222-3p**				
Non-small cell lung cancer	PUMA	BBC3 pathway	Proliferation and Apoptosis	[92]
Renal clear cell carcinoma	TMP2	ERK pathway	Invasion	[93]

Oral squamous cell carcinoma	CDKN1B		Proliferation, migration, and invasion	[94]
Renal cell carcinoma	SNX4, MYLIP, PAIP2, PLEKHA2		Migration, invasion, and apoptosis	[95]
Diffuse large b-cell lymphoma	PPP2R2A		Proliferation, and apoptosis	[96]
Non-small cell lung cancer	SOCS3	JAK/STAT signaling pathway	Drug resistance	[97]
Epithelial ovarian cancer	GNAI2	Akt signaling pathway	Proliferation and migration	[98]
Epithelial ovarian cancer	SOCS3	SOCS3/STAT3 pathway	Progression	[99]
Endometrial carcinoma	Erα		Proliferation and invasion	[100]
